# Multiplex Amplicon Quantification (MAQ), a fast and efficient method for the simultaneous detection of copy number alterations in neuroblastoma

**DOI:** 10.1186/1471-2164-11-298

**Published:** 2010-05-12

**Authors:** Candy Kumps, Nadine Van Roy, Lien Heyrman, Dirk Goossens, Jurgen Del-Favero, Rosa Noguera, Jo Vandesompele, Frank Speleman, Katleen De Preter

**Affiliations:** 1 Center for Medical Genetics, Ghent University Hospital, Ghent, Belgium; 2 Applied Molecular Genomics Group, Department of Molecular Genetics, VIB, Belgium; 3 University of Antwerp (UA), Antwerp, Belgium; 4 Department of Pathology, Medical School of Valencia, University of Valencia, Valencia, Spain

## Abstract

**Background:**

Cancer genomes display characteristic patterns of chromosomal imbalances, often with diagnostic and prognostic relevance. Therefore assays for genome-wide copy number screening and simultaneous detection of copy number alterations in specific chromosomal regions are of increasing importance in the diagnostic work-up of tumors.

**Results:**

We tested the performance of Multiplex Amplicon Quantification, a newly developed low-cost, closed-tube and high-throughput PCR-based technique for detection of copy number alterations in regions with prognostic relevance for neuroblastoma. Comparison with array CGH and the established Multiplex Ligation-dependent Probe Amplification method on 52 neuroblastoma tumors showed that Multiplex Amplicon Quantification can reliably detect the important genomic aberrations.

**Conclusion:**

Multiplex Amplicon Quantification is a low-cost and high-throughput PCR-based technique that can reliably detect copy number alterations in regions with prognostic relevance for neuroblastoma.

## Background

Recurrent alterations in DNA copy number are a common feature in many cancers and typically target biological pathways and processes that contribute to cancer pathogenesis. The diagnostic and prognostic relevance of these chromosomal imbalances (gains, losses and amplifications) has been demonstrated in an increasing number of tumor entities [1]. For the detection of unbalanced chromosomal aberrations different profiling platforms are commercially available. Array CGH is a commonly applied high-resolution genome-wide screening method for research and diagnostic purposes [2] but currently remains relatively expensive and labor-intensive. Alternatively, PCR-based methods for the simultaneous detection of copy number changes in selected chromosomal regions such as Multiplex Ligation-dependent Probe Amplification (MLPA) [3] are well established and suited to be applied on a routine basis [4].

In this study, we evaluated the performance of a new method for relative quantification of specific DNA sequences in routine laboratory practice, called Multiplex Amplicon Quantification (MAQ) [5-9]. For this purpose, a MAQ assay was specifically designed for neuroblastoma (NB), the most common solid extra-cranial pediatric malignancy [10]. This is a good model for MAQ validation as intensive research of genomic imbalances has revealed insights into the clinical and biological heterogeneity of this tumor [11]. More specifically, prognostic relevant, critical regions of loss and gain in NB have been delineated, the most important being 1p deletion, *MYCN *amplification, 3p deletion, 11q deletion and 17q gain [12-16]. Of further importance, new therapeutic protocols based on the presence or absence of these segmental abnormalities are in progress [16]. The MAQ-NB assay allows the detection of CNAs in the prognostic relevant regions in NB. In this study, MAQ results were compared to copy number changes detected with MLPA on a series of 52 NB for which detailed array CGH profiles were available.

## Methods

### Neuroblastoma tumor samples and cell lines

A representative series of NB tumor samples was collected prior to therapy (34 cases from the Ghent University Hospital, Belgium and 14 from the Medical School of Valencia, Spain) (see Additional File 1). Of these 48 NB samples, 17 samples present with only numerical and 31 with segmental aberrations (sometimes in a numerical background). *MYCN *amplification occurs in 19 cases. Additionally, 4 NB cell lines with segmental aberrations of which 3 with *MYCN *amplification were included in this study (NB-19, GI-CI-N, SJ-NB-10, LAN-6). DNA was isolated from NB tumors, cell lines and control samples using the Qiagen DNA isolation kit (QIAgen) according to the manufacturer's instructions.

### Multiplex Ligation Probe Amplification (MLPA)

**Figure 1:**
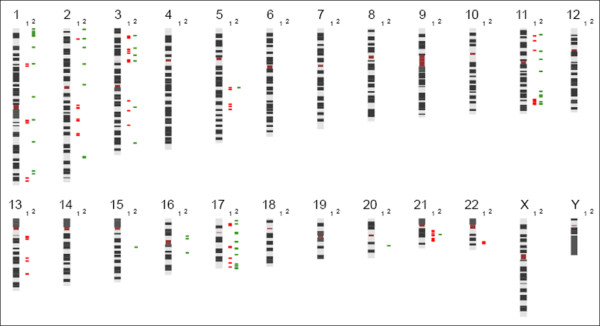
**Karyo view indicating regions covered by MAQ (1) and MLPA amplicons (2)**.

For MLPA analysis, the SALSA NB kits P251 (targeting chromosome 1, 3 and 11) and P252 (targeting chromosome 2 and 17) (MRC-Holland) were used to investigate DNA aberrations in specific chromosome regions of interest for NB [17]. The third NB kit P253, targeting chromosome 4, 7, 9, 12 and 14, was not used for this study. In the case of *MYCN *amplified NB samples, a *MYCN *silencing solution was added. Target regions are indicated in Figure 1. 100 ng of tumor DNA was used for each MLPA reaction. In each experiment three control samples were analysed, allowing accurate normalisation, including an EBV cell line, constitutional blood from a healthy individual and human genomic DNA (Roche). MLPA was performed according to the manufacturer's protocol with minor adaptations in the PCR step. Briefly, PCR was performed using 10 μl of the ligase-product and 40 μl of PCR-mix containing 2 μl of SALSA primers, 2 μl SALSA enzyme dilution buffer, 4 μl SALSA PCR buffer and 0.5 μl polymerase, diluted in HPLC water, prepared on ice.

### Multiplex Amplicon Quantification (MAQ)

For MAQ analysis, three kits were designed, MAQ1, MAQ2 and MAQ3 as described in the results section. 50 ng of tumor DNA was needed for each reaction of the MAQ analysis. For accurate normalisation, two control samples were included in each experiment, i.e. constitutional DNA from a healthy individual and human genomic DNA (Roche). After addition of 10 μl of PCR mix and 0.075 μl of Taq polymerase, samples were heated in a thermocycler with a heated lid (PTC200, Bio-Rad) for 2 minutes at 98°C for activation of the polymerase. Further PCR conditions required 22 cycles, including denaturation at 95°C for 45 seconds, annealing at 60°C for 45 seconds and extension for 2 minutes at 68°C. This was followed by a final extension step at 72°C for 10 minutes.

### Capillary electrophoresis and data analysis

For both MLPA and MAQ, resulting PCR products were analysed by capillary electrophoresis. A mixture of 8.5 μl formamide (HiDi, Applied Biosystems) and 0.5 μl internal lane standard (Rox500, Applied Biosystems) was prepared and 1 μl of the PCR-product was added. After a 2 minute denaturation-step at 95°C, the samples were analyzed on the ABI3130XL (Applied Biosystems) capillary electrophoresis system. The raw data generated by fragment analysis were analysed in a specially designed software program Coffalyser [18] and MAQ-S [5] for MLPA and MAQ, respectively. Both programs were designed to calculate and visualise the normalised peak area or dosage quotient which reflects copy number of each target amplicon. Dosage Quotients (DQ) are calculated using the reference amplicons (in regions with low occurrence of aberrations) and the control samples (without genomic aberrations). For one reference amplicon (RefA) and one control sample (ctr), the DQ of a target amplicon (TarA) in sample (s) is as follows: DQ = [TarA(s)/RefA(s)]/[TarA(ctr)/RefA(ctr)]. Based on the data of all reference amplicons and control samples, a final DQ is calculated as the mean of the individual DQs and the standard deviation of the DQs (visualized as error bars) is a measure of stability.

### Scoring rules for MLPA and MAQ

Optimal scoring thresholds were applied for MLPA and MAQ. For MLPA scoring (linear scale) we applied the following criteria. Regions were scored as segmental deletions or gains if the dosage quotients of at least 2 consecutive loci is 0.25 below or above 1, respectively [19]*or *when more than 75% of the probes show a decrease or increase of at least 0.15, respectively. When no segmental aberrations were detected in the tumor, the presence of numerical changes was evaluated. Regions are scored as whole chromosomal losses or gains if at least 75% of the dosage quotients of both p and q arm have a value below or above 1, respectively *and *more than half of the dosage quotients show an decrease or a increase of at least 0.1. MYCN amplification is scored if the 2 dosage quotients for the MYCN locus increase with at least 3. For MAQ scoring (linear scale) we applied the following criteria. Regions were scored as segmental deletions or gains if the dosage quotients of at least 2 consecutive loci is 0.2 below or above 1, respectively (according to the manufacturer's instructions). When no segmental aberrations were detected in the tumor, the presence of numerical changes was evaluated. Regions are scored as whole chromosomal losses or gains if at least 75% of the dosage quotients of both p and q arm have a value below or above 1, respectively and more than half of the dosage quotients show an decrease or an increase of at least 0.1. For scoring of MYCN amplification the 3 dosage quotients for the MYCN locus should increase with at least 3.

### Array Comparative Genomic Hybridisation (array CGH)

Samples were profiled on an in-house developed 1 Mb resolution BAC array as previously described [13] (18 samples) or on a custom designed 44K array (Agilent Technologies) (34 samples) enriched for critical regions in NB (e.g. 1p, 2p, 3p, 11q, 17). Utilizing random prime labeling (BioPrime ArrayCGH Genomic Labeling System, Invitrogen), 400 ng of tumor and control DNA (DNA from an EBV cell line or male control DNA, Promega) was labeled with Cy3 and Cy5 dyes (GE healthcare). Further processing was then performed according to the manufacturer's instructions (Agilent Technologies). Slides were scanned using an Agilent scanner (Agilent Technologies), features were extracted using the feature extraction v10.1.1.1 software program and further processed with an in-house developed visualisation software program arrayCGHbase [20,21]. Array CGH profiles were evaluated manually after application of the circular binary segmentation (CBS) algorithm, which is an algorithm to dissect genomic array data into regions of equal copy number by applying a maximal t-statistic with a permutation reference distribution to determine the change-points [22]. Gains are indicated in green (CBS value > 0.3); losses are indicated in red (CBS value < -0.3).

## Results

### Construction of the MAQ neuroblastoma assay

The MAQ technique consists of the quantification of fluorescently labelled test and control amplicons, obtained by a single multiplex PCR (mPCR) amplification. As a first step in the construction of the MAQ neuroblastoma assay, primers were designed in the critical regions of loss (1p, 3p and 11q) and gain (2p -more specifically the *MYCN *locus- and 17q) for NB and their respective opposite chromosome arm [12,13,15]. Specialised software [5] was used for the development of PCR primer sets within the chromosome regions of interest with a very high multiplexing degree, targeting up to 40 sequences each with a unique length. In this way, the NB-MAQ assay was created consisting of three kits. MAQ1 contains a total of 21 primer pairs with target regions on chromosome 3 and 17 and MAQ2 contains 19 primer pairs targeting chromosome 1 and 11. For the detection of *MYCN *(2p) status, a separate kit (MAQ3) was designed, containing 9 primer pairs for chromosome 2. This was necessary since the high levels of (*MYCN*) amplification interfered with reliable analysis of other amplicons generated in the multiplex PCR. Additionally, each kit includes 8 or 9 primer sets, covering chromosomal regions that show the lowest frequency of copy number changes in NB (chromosome 5q, 13q, 21q, 22q) and serve as reference sequences used for accurate normalisation. These regions were carefully selected through screening of the chromosomal status on array CGH profiles of 1000 NB cases (De Preter et al., in preparation). The MAQ target regions are indicated in Figure 1. More detailed information on the target regions of the MAQ kits can be found in Additional File 2.

### Evaluation of the MAQ assay by comparison with MLPA and array CGH

**Figure 2:**
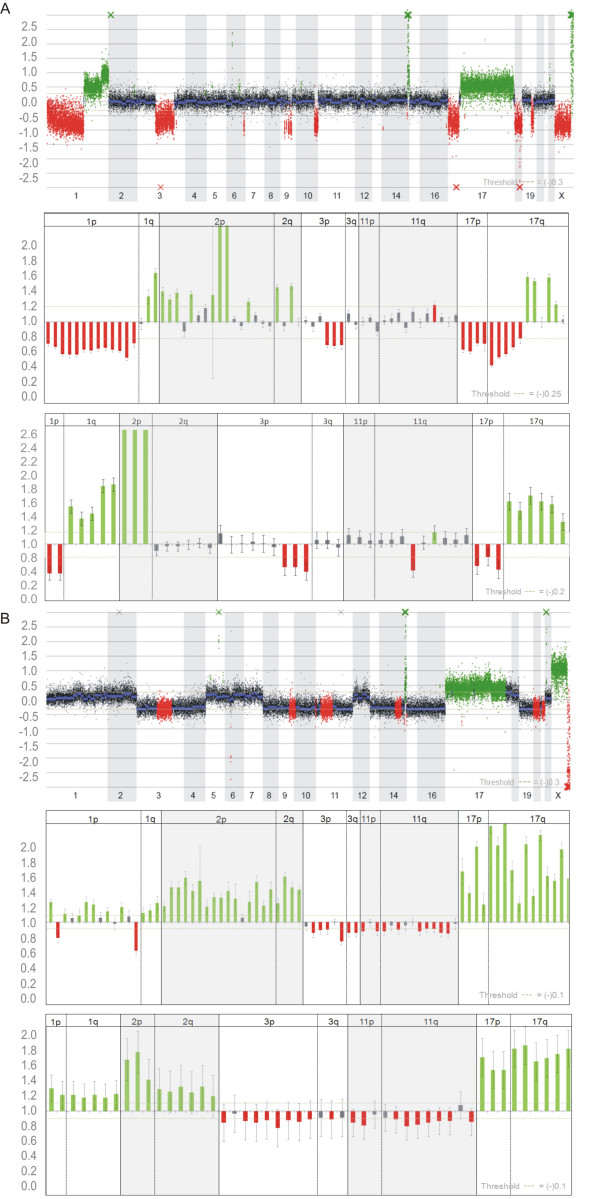
**Two representative examples of aCGH, MLPA and MAQ NB tumor profiles A**. NB cell line SJNB-10 with segmental aberrations, including 1p deletion, MYCN amplification, 3p deletion, 17q gain. **B**. NB tumor with numerical aberrations including whole chromosome 1, 2 and 17 gain, and whole chromosome 3 and 11 loss. Gray = normal; Red = loss; Green = gain (according to scoring thresholds). Horizontal bars in array profiles indicate CBS values. Red and green cross marks point at CBS values above or below 0.3 or -0.3, respectively. In Figure 2B all crosses indicate known copy number variations. Error bars of each dosage quotient (DQ) are based on the standard deviation after summarizing the DQ values using different reference amplicons and 3 (MLPA) or 2 (MAQ) control samples.

The performance of the MAQ assay for the detection of CNAs in prognostic relevant regions was evaluated by comparing results of 48 NB tumor samples and 4 NB cell lines with corresponding MLPA and array CGH profiles (Additional File 3). For this analysis, each sample was screened once and scoring was applied as described in the Methods section. Based on array CGH results (Additional File 4), 2 groups were defined. One group consists of 17 tumor samples which presented exclusively with numerical aberrations (whole chromosome gains and losses), whereas the second group of 35 samples was characterised by the presence of segmental aberrations, sometimes in a numerical background (15/35). Two NB tumor profiles representative for each subgroup are shown in Figure 2, illustrating high concordance between array CGH, MLPA and MAQ results.

Performance of MLPA and MAQ for the detection of aberrations in each of the prognostic relevant regions is summarised in Table 1. The area under the curve (AUC) of the receiver operating curve (ROC) was used as a measure for accuracy, summarizing the sensitivity and specificity for a given test. High-quality tests with almost perfect performance will have an AUC approaching 100%. ROC curves are shown in Additional File 5. Array CGH was used as golden standard for these analyses. For the subset of tumors with segmental aberrations, performance for detecting 1p, *MYCN*, 3p, 11q and 17q status was very high for both techniques with average AUC of 97.2% and 98.9% for MLPA and MAQ, respectively. The false positive rate for MLPA and MAQ was 4.7% and 0.9%, respectively. For MLPA, no false negatives were detected while for MAQ one false negative was detected at chromosome 1p (false negative rate of 1.1%). It should be noted that the reference amplicons in this individual were all located in regions with a low level loss, therefore the lost 1p region appeared as normal.

**Table 1:** Performance of MLPA and MAQ

	Chromosome 1p		*MYCN *status		Chromosome 3p		Chromosome 11q		Chromosome 17q	
**Segmental (n = 35)**	**MLPA**	**MAQ**	**MLPA**	**MAQ**	**MLPA**	**MAQ**	**MLPA**	**MAQ**	**MLPA**	**MAQ**

**AUC**	94.4%	97.1%	100.0%	100.0%	94.3%	100.0%	97.1%	97.1%	100.0%	100.0%
**Sensitivity**	100.0%	94.4%	100.0%	100.0%	100.0%	100.0%	100.0%	100.0%	100.0%	100.0%
**Specificity**	88.9%	100.0%	100.0%	100.0%	92.0%	100.0%	95.5%	95.5%	100.0%	100.0%
**PPV**	90.0%	100.0%	100.0%	100.0%	83.3%	100.0%	92.9%	92.9%	100.0%	100.0%
**NPV**	100.0%	94.4%	100.0%	100.0%	100.0%	100.0%	100.0%	100.0%	100.0%	100.0%

	***95% Confidence Interval AUC(MLPA): 94%-100%***						***95% Confidence Interval AUC(MAQ): 97%-100%***			

	**Chromosome 1**		**Chromosome 2**		**Chromosome 3**		**Chromosome 11**		**Chromosome 17**	

**Numerical (n = 17)**	**MLPA**	**MAQ**	**MLPA**	**MAQ**	**MLPA**	**MAQ**	**MLPA**	**MAQ**	**MLPA**	**MAQ**

**AUC**	76.5%	88.2%	92.3%	76.9%	58.8%	76.5%	70.6%	76.5%	94.1%	94.1%
**Sensitivity**	77.8%	77.8%	90.0%	70.0%	46.2%	69.2%	71.4%	71.4%	94.1%	94.1%
**Specificity**	75.0%	100.0%	100.0%	100.0%	100.0%	100.0%	66.7%	100.0%	100.0%	100.0%
**PPV**	77.8%	100.0%	100.0%	100.0%	100.0%	100.0%	90.9%	100.0%	100.0%	100.0%
**NPV**	75.0%	80.0%	75.0%	50.0%	36.4%	50.0%	33.3%	42.9%	100.0%	100.0%

	***95% Confidence Interval AUC(MLPA): 60%-97%***						***95% Confidence Interval AUC(MAQ): 72%-93%***			

Performance in tumors with segmental (35) and numerical (17) aberrations for the recurrent NB copy number changes at chromosome 1(p), 2 (*MYCN *status), 3(p), 11(q) and 17(q). This evaluation includes area under curve (AUC), positive predictive value (PPV) and negative predictive value (NPV).

For the 17 cases presenting with only numerical aberrations, performance was evaluated for whole chromosome changes. Overall, performance rates were less optimal for these tumors with average AUC of 78.5% and 82.5% for MLPA and MAQ, respectively. When looking more into detail, the AUC remains high for chromosome 17 for both techniques (AUC is 0.941), demonstrating that high level gains are easily detected. While lower performance is seen for chromosome 1, 2, 3 and 11. As well for array CGH as for MLPA and MAQ, detection of whole chromosome gains and losses in near-triploid tumors is challenging. Nevertheless, these aberrations can be detected rather unambiguously with array CGH due to the high amount of oligos used on these platforms (as can be seen in Figure 2B). This problem is inherent to characteristics in NB tumors with numerical aneuploidy where many chromosomes are implicated, including those used as reference for normalisation. No false positives were detected with MAQ and the false positive rate for MLPA was 11.7%, while the false negative rate was 23.5% and 24.1%, respectively. Importantly, for these near-triploid tumors the only clinically relevant question is whether any segmental aberrations are detected and this is not the case when MAQ is used.

Furthermore, repeatability of MAQ measurements was tested by performing Bland-Altman analysis [23]. From this method the coefficient of repeatability was derived by calculating the average difference ± 1.96 standard deviation of the difference. MAQ1 measurements of 6 samples (4 tumors with segmental aberrations and 2 with numerical aberrations) were found reliably repeatable. The coefficient of repeatability was 0.121 for the tumors with segmental and 0.0987 for the tumors with numerical imbalances and therefore fall within the threshold boundaries used in the scoring procedure.

## Discussion

MAQ is a new PCR-based method that allows to determine the copy number status of multiple loci in a single assay. Similar to MLPA, this technique fills the gap between more expensive genome-wide screening assays and cheaper methods that only provide information on a single locus. We validated MAQ in a model for NB as an alternative for MLPA, using array CGH data as a reference point. This study shows that MAQ is a robust and repeatable method for detection of prognostic relevant CNAs in recurrently affected regions in NB tumors and cell lines. For all investigated samples, *MYCN *amplification status, which is one of the strongest prognostic parameters, was accurately determined. In addition, MAQ reliably differentiates between tumors with segmental aberrations (which are correlated to poor prognosis) and tumors with numerical aberrations (correlated to good prognosis) [15]. For the detection of segmental gains and losses of one or more copies in 35 NB samples, performance was very high even when measured in a numerical background (Table 1). Of notice, the few discrepancies measured (both for MLPA and MAQ) could represent very small aberrations, which we anticipate to be very rare. The validity of such putative small aberrations might be examined by MLPA or MAQ assays enriched for probes in this region, ultra-high resolution arrays with dense coverage in this region or even next generation sequencing but this lies beyond the scope of our study. Detection of whole chromosome imbalances in near-triploid tumors is more challenging for both array CGH and MLPA and MAQ. In general the presence of multiple gains and losses in these tumors hampers normalisation [24]. In our study, 16 out of 17 tumors with numerical aberrations (and even in 15/35 with segmental), one or more reference chromosomes show CNAs, thus further complicating accurate normalisation for MAQ. In the case of NB, the pattern of these tumors is clearly recognisable [13]. In addition, for prognostic purposes, it is not of importance to know which chromosomes are numerically aberrant but whether a distinction can be made between tumors with segmental and numerical aberrations. Importantly, not a single false positive segmental aberration was detected by MAQ in near-triploid tumors with only numerical aberrations. In addition, whole chromosome 17 gain detection which is one of the typical characteristics of these tumors, was almost perfectly detected, thus allowing assignment of these tumors to the prognostic favorable category.

## Conclusion

We demonstrated that the newly developed MAQ method can be used as a valuable diagnostic tool for reliable detection of copy number changes with prognostic relevance in NB. Overall, PCR-based techniques harbor advantages in comparison to array CGH such as reduced expenses and sample-handling while maintaining high performance. Moreover the equipment needed, i.e. a thermal cycler and a capillary electrophoresis system, is present in the majority of molecular biology laboratories performing routine diagnostics. As MAQ allows to measure copy number status of as much as 40 targets in one reaction, this is sufficient for many routine tests for which only a well defined and specific number of imbalances needs to be analysed. When comparing MLPA to MAQ, the latter method may have certain advantages (see Table 2). It requires less DNA, reduced handling, experiment time and costs, while it occurs in a single closed-tube reaction, maximally limiting contamination problems. Moreover, this test appears slightly more robust in our hands. At present, guidelines for the diagnostic work-up of NB recommend two independent molecular methods for the assessment of genetic alterations in NB [16]. In this context MAQ can be very well suited as complementary method to array CGH or FISH.

**Table 2:** Overview of parameters important for aCGH, MLPA and MAQ

	Array CGH (44 K)	MLPA	MAQ
**Number of sequences investigated per experiment**	44000 DNA sequences	up to 45 DNA sequences (5 control probes)	up to 40 DNA sequences (9-10 control amplicons)

**Number of reactions**	1	2	3

**Input DNA**	150-400 ng DNA/reaction	100-200 ng DNA/reaction	50 ng DNA/reaction

**Throughput**	intermediate	high	very high

**Experiment time**	results within 72 h	results within 24 h	results within 6 h

**Hands-on time**	high	medium	low

**Detection of numerical aberrations**	+	+/-	+/-

**Material**	oven, array scanner, software	thermocycler and capillary electophoresis system	thermocycler and capillary electophoresis system

Consumables* (patient and reference samples included)	205 € (1 control sample included)	88 € (3 control** samples included)	66 € (2 control** samples included)

*For the price calculation we envisioned an experiment in which only 1 NB sample is screened which is reasonable as NB is a very infrequent tumor (approximately 20 new cases in Belgium every year)

**The MLPA protocol recommends to use at least 3 control samples while 2 control samples are sufficient for MAQ according to the manufacturers

## List of abbreviations

(AUC): Area under the curve; (CBS): Circulary Binary Segmentation; (MAQ): Multiplex Amplicon Quantification; (MLPA): Multiplex ligation-dependent probe amplification; (mPCR): Multiplex PCR; (NPV): Negative predictive value; (NB): Neuroblastoma; (PPV): Positive predictive value; (ROC): Receiver Operating Characteristic.

## Authors' contributions

CK carried out the experimental work and data analysis, for the array, MLPA and MAQ experiments. NVR participated in the design of the study and helped to draft the manuscript. LH, DG and JDF were involved in the design of the MAQ method. KDP provided the NB regions of interest necessary for the development of the MAQ NB assay. JVDS, FS and KDP conceived of the study and participated in its design and coordination and helped to draft the manuscript. All authors read and approved the final manuscript.

## Supplementary Material

Additional file 1 **Patient data on 48 neuroblastoma tumors**. Patient data on 48 neuroblastoma tumors including INSS stage, age at diagnosis, MYCN amplification status and survival status. Click here for file

Additional file 2 **Overview primer pools of MAQ1 (A), MAQ2 (B) and MAQ3 (C)**. Overview of primer pools used for MAQ1 (A), MAQ2 (B) and MAQ3 (C) including details of chromosomal location Click here for file

Additional file 3 **Scoring table of 48 tumors and 4 cell lines with segmental (A) and numerical (B) aberrations**. Scoring table of 31 tumors and 4 cell lines with segmental aberrations (A). Thirty-five tumors display with segmental aberrations. Scoring was performed at the regions of interest at 1p, MYCN amplification (MNA) status, 3p, 11q and 17q. Scoring table of 17 tumors with exclusively numerical aberrations (B). Seventeen tumors display with only numerical aberrations. Scoring was performed at the regions of interest at for both chromosome arms for chromosome 1, 2, 3, 11 and 17. Red indicates clear loss, light red indicates a loss where the threshold is barely reached, light green indicates a gain where the threshold is barely reached, green indicates clear gain. Black indicates that there was no data obtained. Click here for file

Additional file 4 **Array CGH results of 48 NB samples and 4 cell lines**. Array CGH results of 48 NB samples and 4 cell lines that were profiled on 44 K oligoarray or BAC array Click here for file

Additional file 5 **Overview of ROC curves of MLPA and MAQ assays versus array CGH**. Overview of ROC curves of MLPA and MAQ assays versus array CGH at 1p, 2p, 3p, 11q and 17q for tumors with segmental and numerical aberrations. Click here for file
