# Supplementary material for: Multiplex Amplicon Quantification (MAQ), a fast and efficient method for the simultaneous detection of copy number alterations in neuroblastoma
Source: BMC Genomics. 2010 May 12;11:298. doi: 10.1186/1471-2164-11-298 (PMC2879279; doi:10.1186/1471-2164-11-298)
Supplement: Additional file 1 — Patient data on 48 neuroblastoma tumors. Patient data on 48 neuroblastoma tumors including INSS stage, age at diagnosis, MYCN amplification status and survival status. [file 1471-2164-11-298-S1.DOC]

**Additional File 1. Patient data on 48 neuroblastoma tumors**
