# Supplementary material for: Multiplex Amplicon Quantification (MAQ), a fast and efficient method for the simultaneous detection of copy number alterations in neuroblastoma
Source: BMC Genomics. 2010 May 12;11:298. doi: 10.1186/1471-2164-11-298 (PMC2879279; doi:10.1186/1471-2164-11-298)
Supplement: Additional file 2 — Overview primer pools of MAQ1 (A), MAQ2 (B) and MAQ3 (C). Overview of primer pools used for MAQ1 (A), MAQ2 (B) and MAQ3 (C) including details of chromosomal location [file 1471-2164-11-298-S2.DOC]

**Additional File 2. Overview primer pools of MAQ1 (A), MAQ2 (B) and MAQ3 (C)**
