# Supplementary material for: Multiplex Amplicon Quantification (MAQ), a fast and efficient method for the simultaneous detection of copy number alterations in neuroblastoma
Source: BMC Genomics. 2010 May 12;11:298. doi: 10.1186/1471-2164-11-298 (PMC2879279; doi:10.1186/1471-2164-11-298)
Supplement: Additional file 3 — Scoring table of 48 tumors and 4 cell lines with segmental (A) and numerical (B) aberrations. Scoring table of 31 tumors and 4 cell lines with segmental aberrations (A). Thirty-five tumors display with segmental aberrations. Scoring was performed at the regions of interest at 1p, MYCN amplification (MNA) status, 3p, 11q and 17q. Scoring table of 17 tumors with exclusively numerical aberrations (B). Seventeen tumors display with only numerical aberrations. Scoring was performed at the regions of interest at for both chromosome arms for chromosome 1, 2, 3, 11 and 17. Red indicates clear loss, light red indicates a loss where the threshold is barely reached, light green indicates a gain where the threshold is barely reached, green indicates clear gain. Black indicates that there was no data obtained. [file 1471-2164-11-298-S3.DOC]

**Additional File 3. Scoring tables** **of 48 tumors and 4 cell lines with segmental and numerical aberrations.
A. Scoring table of 31 tumors and 4 cell lines with segmental aberrations.**

Thirty-five tumors display with segmental aberrations. Scoring was performed at the regions of interest at 1p, *MYCN* amplification (MNA) status, 3p, 11q and 17q. Red indicates clear loss, light red indicates a loss where the threshold is barely reached, light green indicates a gain where the threshold is barely reached, green indicates clear gain.

**B. Scoring table of 17 tumors with exclusively numerical aberrations.**

Seventeen tumors display with only numerical aberrations. Scoring was performed at the regions of interest at for both chromosome arms for chromosome 1, 2, 3, 11 and 17. Red indicates clear loss, light red indicates a loss where the threshold is barely reached, light green indicates a gain where the threshold is barely reached, green indicates clear gain. Black indicates that there was no data obtained.
