# Supplementary material for: Multiplex Amplicon Quantification (MAQ), a fast and efficient method for the simultaneous detection of copy number alterations in neuroblastoma
Source: BMC Genomics. 2010 May 12;11:298. doi: 10.1186/1471-2164-11-298 (PMC2879279; doi:10.1186/1471-2164-11-298)
Supplement: Additional file 4 — Array CGH results of 48 NB samples and 4 cell lines. Array CGH results of 48 NB samples and 4 cell lines that were profiled on 44 K oligoarray or BAC array [file 1471-2164-11-298-S4.DOC]

**Additional File 4. Array CGH results of 48 NB samples and 4 cell lines**


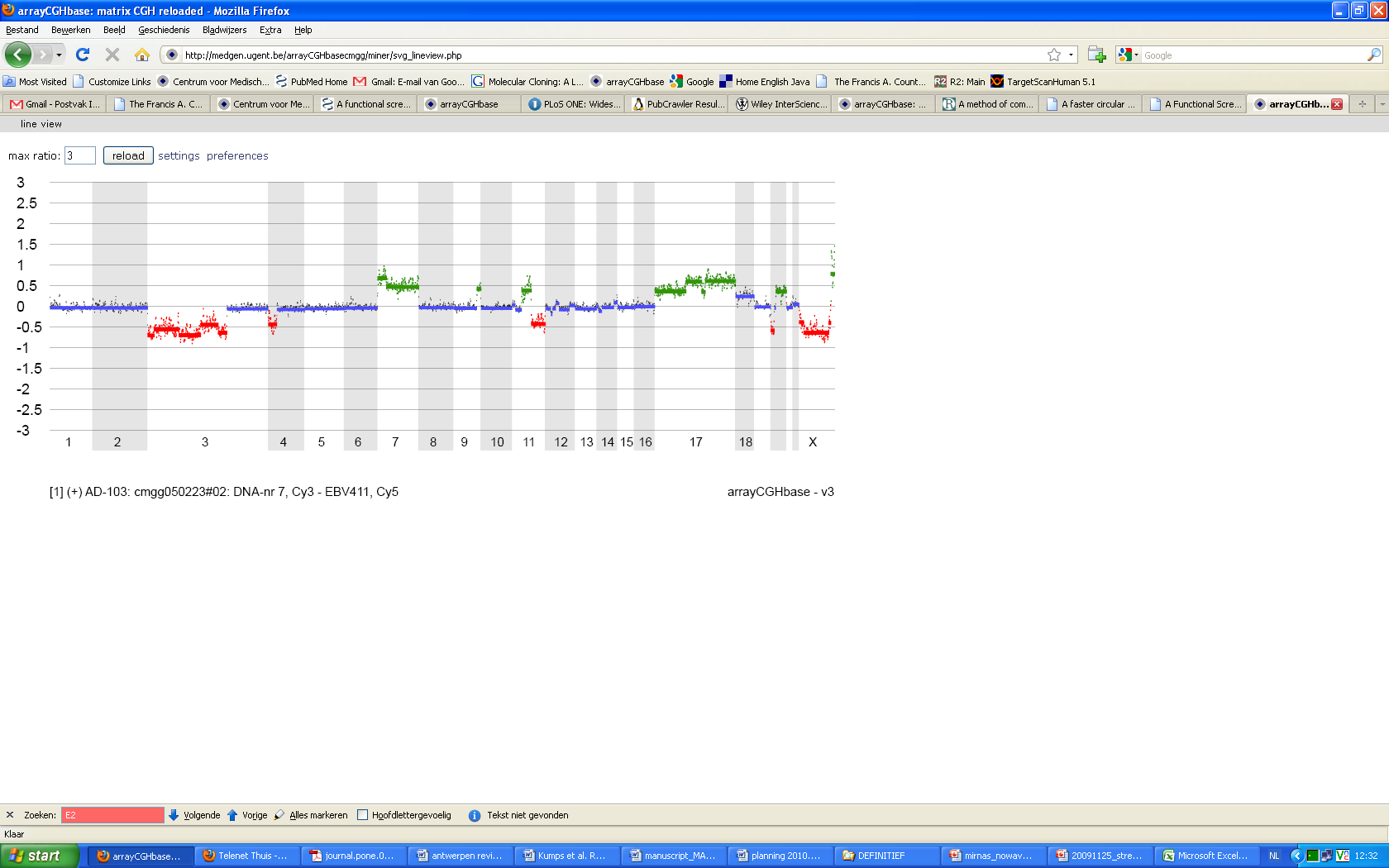
NB_9


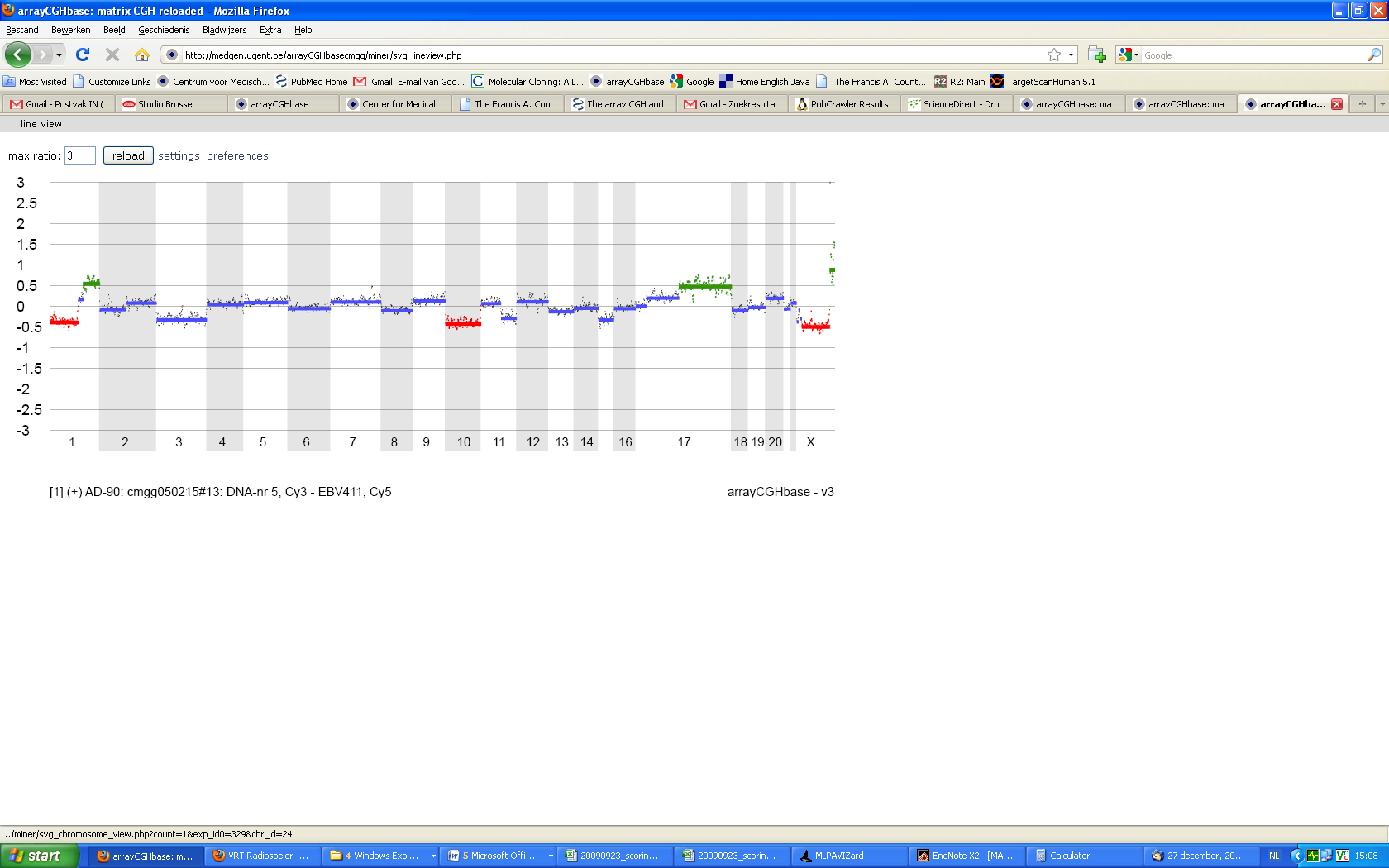
 NB_135


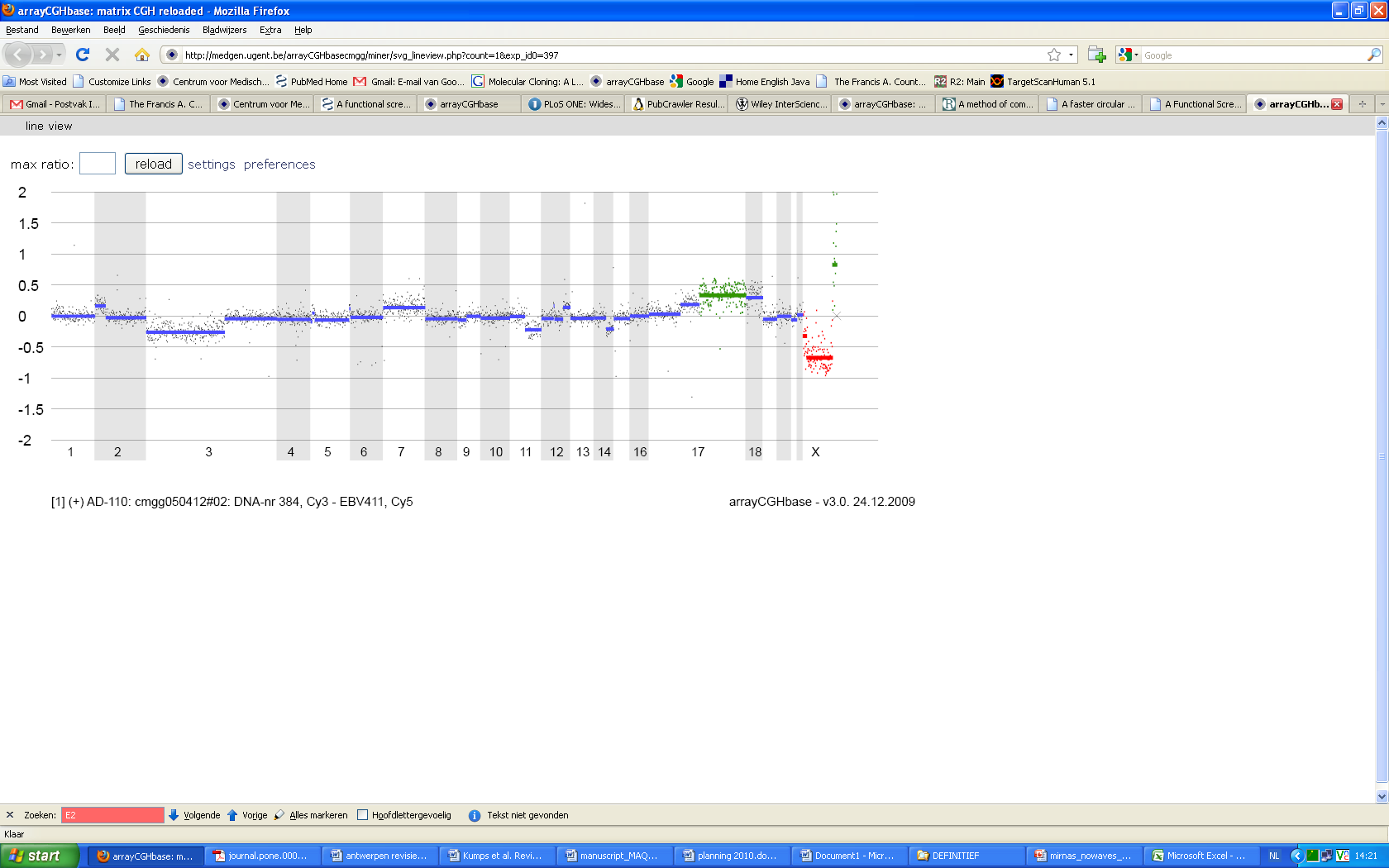
 NB_49
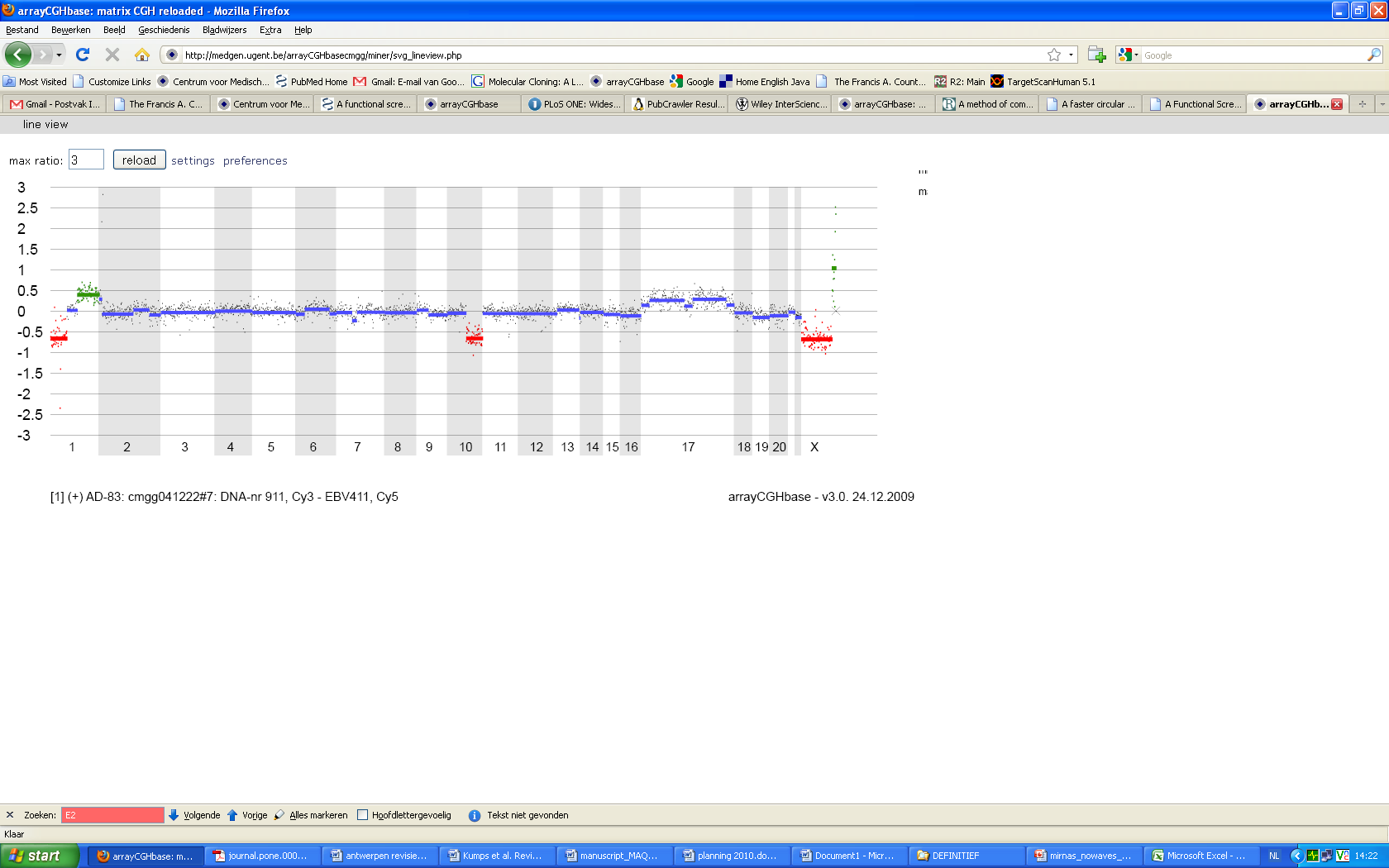
 NB_72


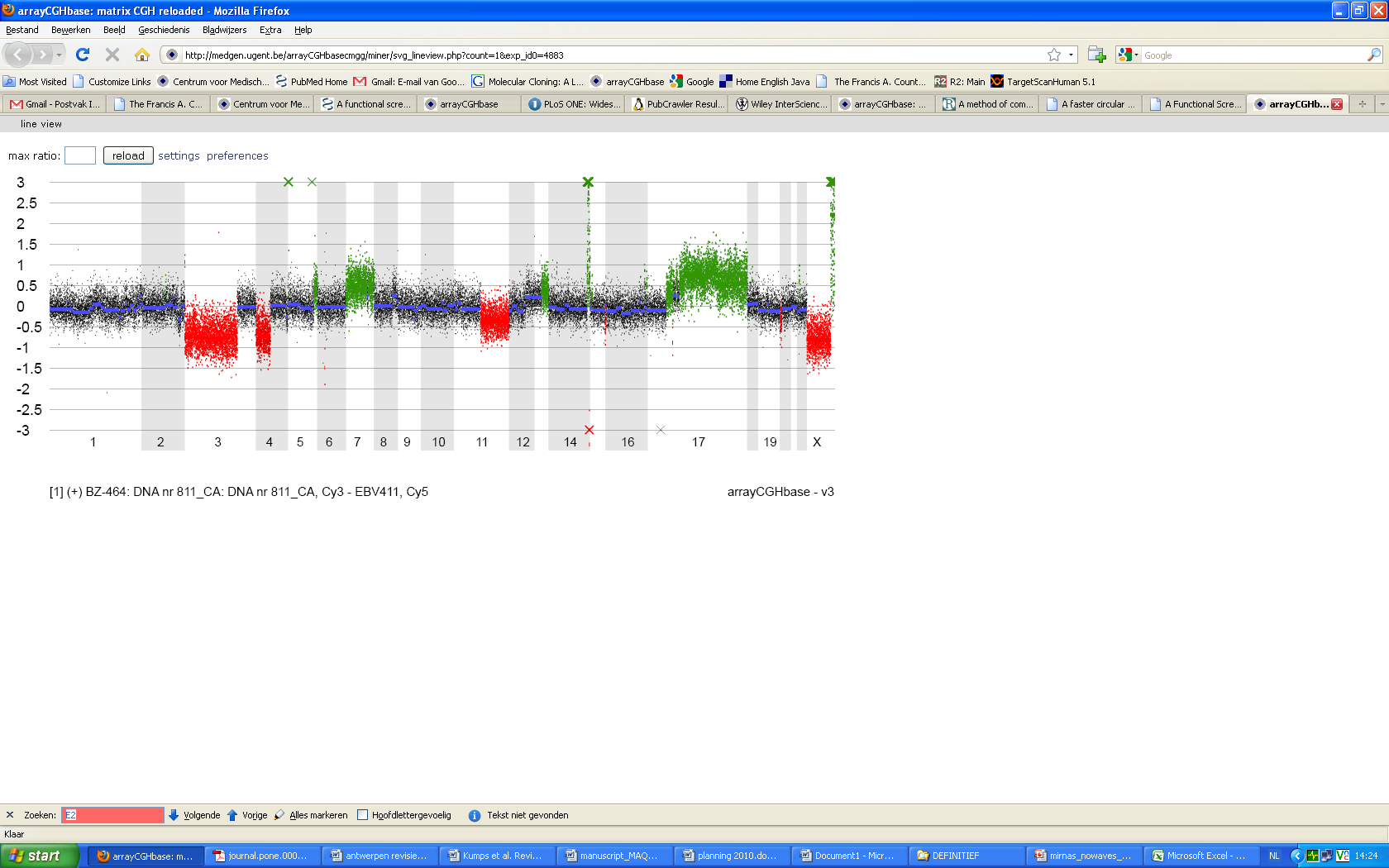
NB_79
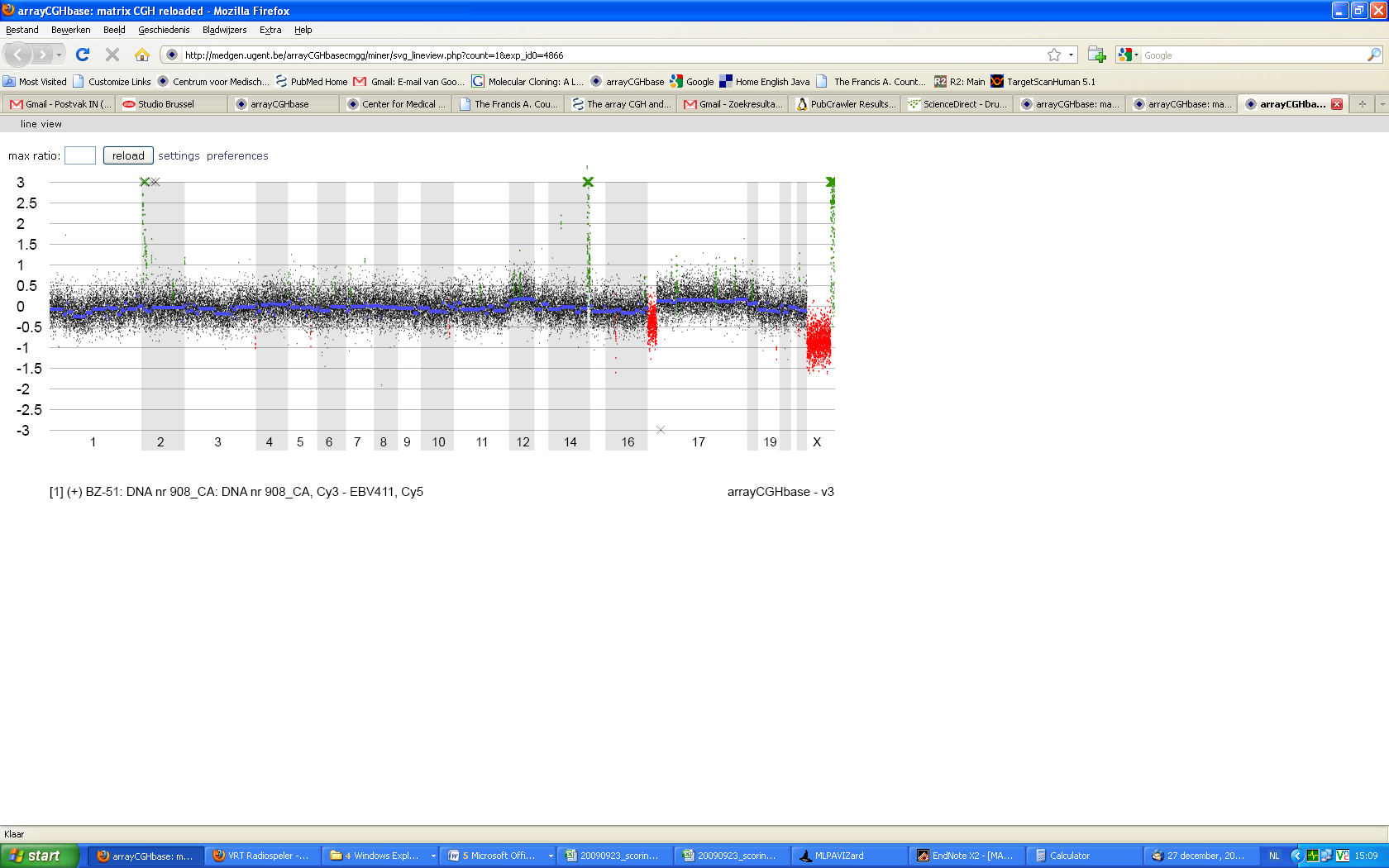
NB_105


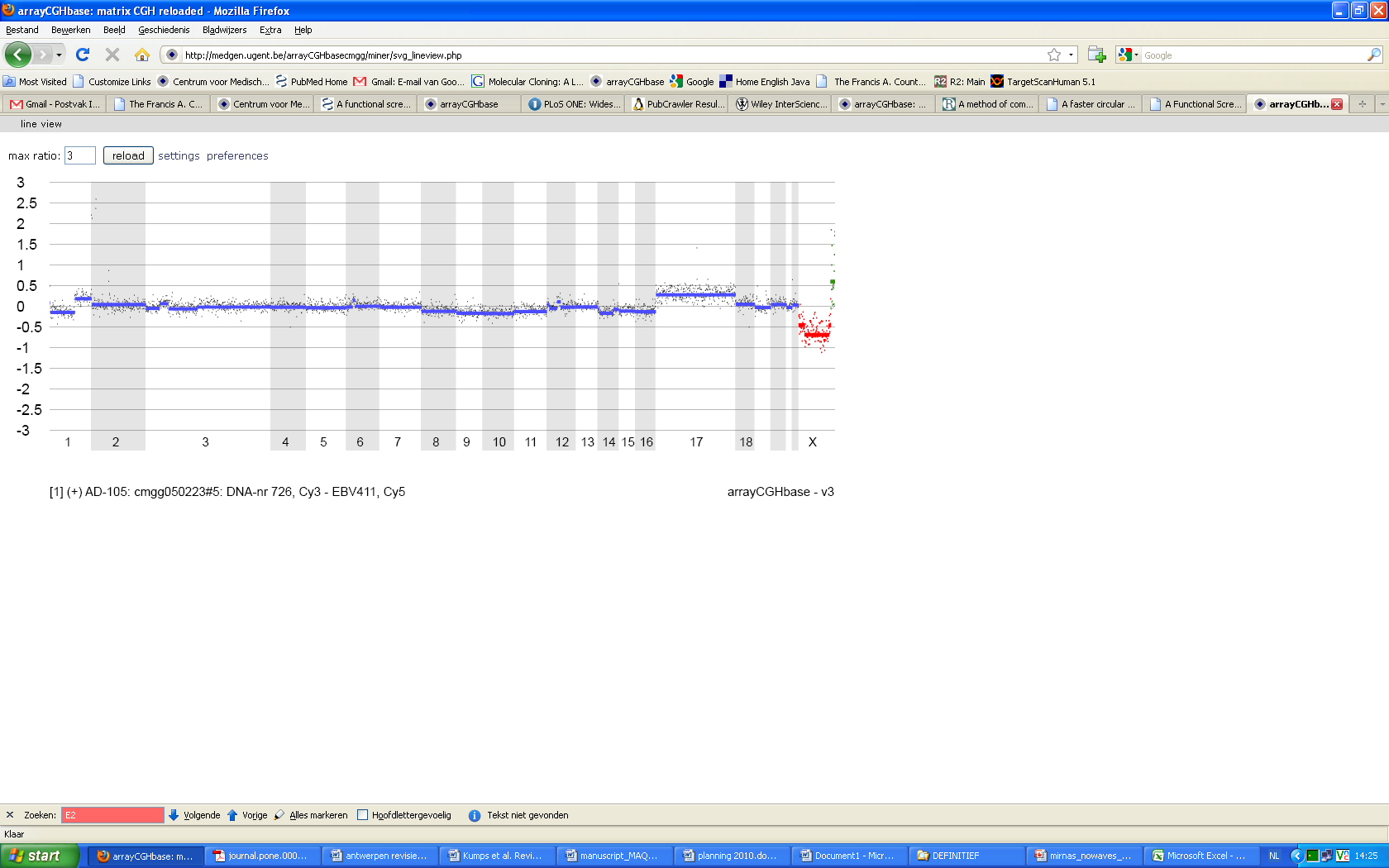
NB_127


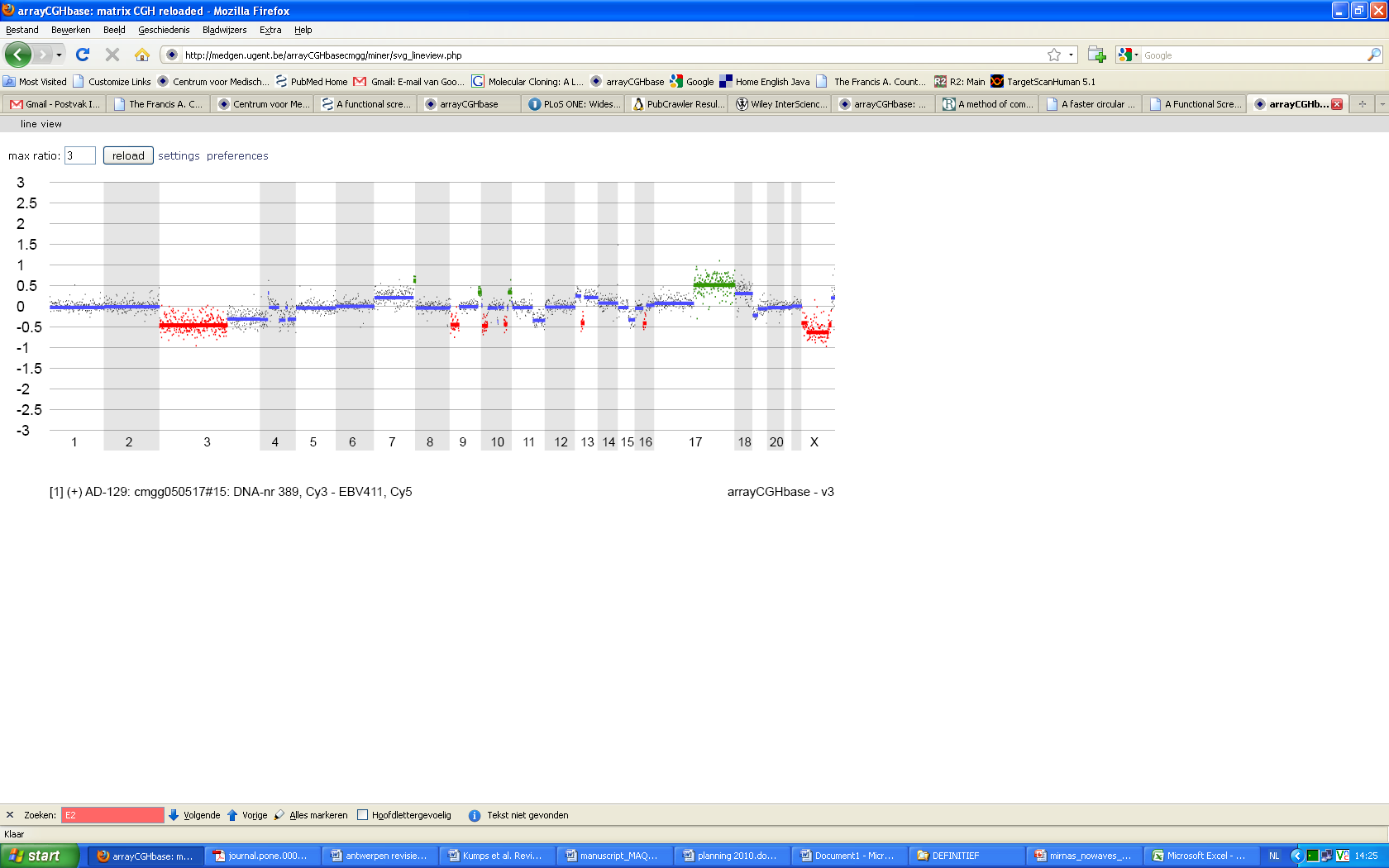
 NB_129


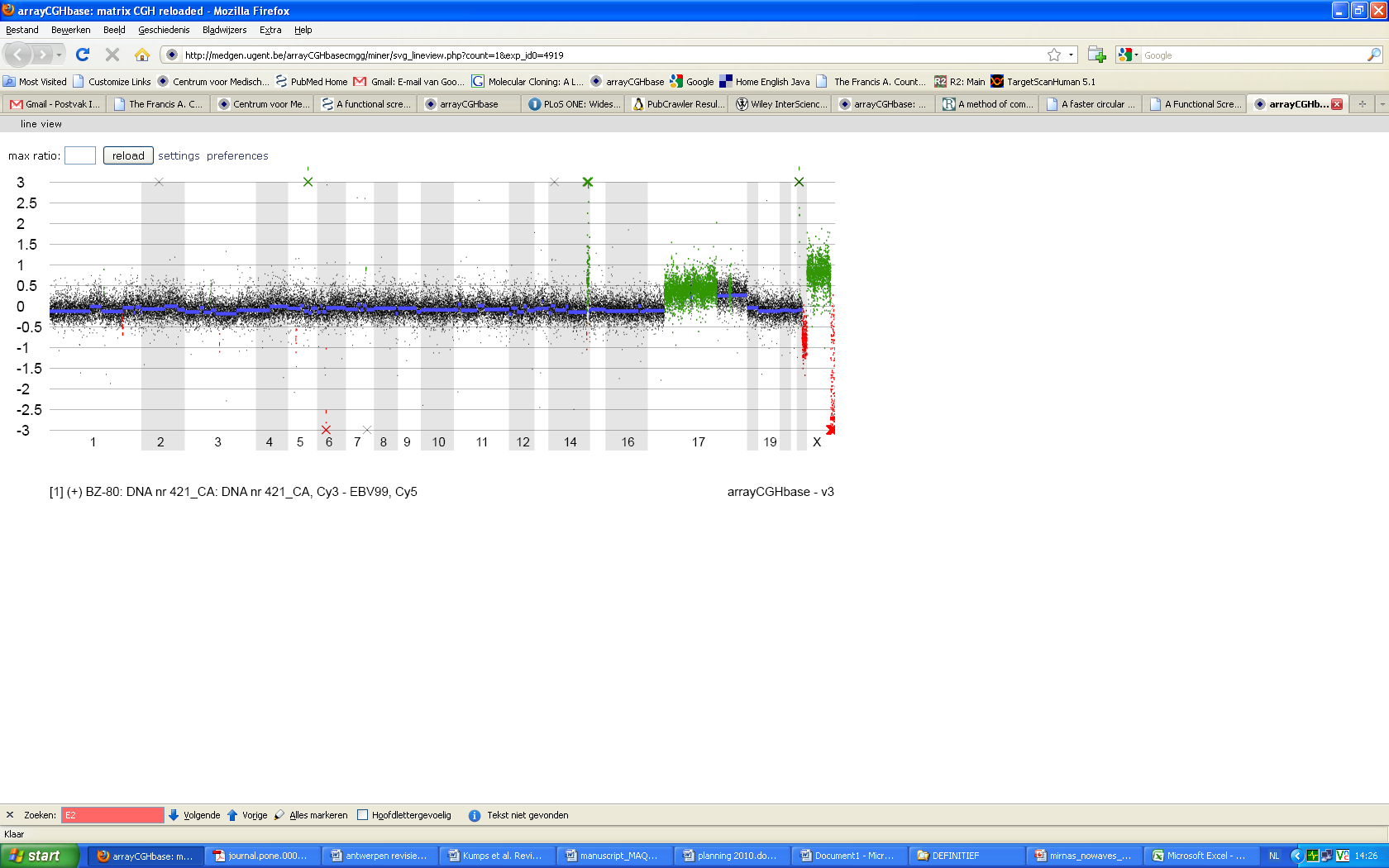
NB_142


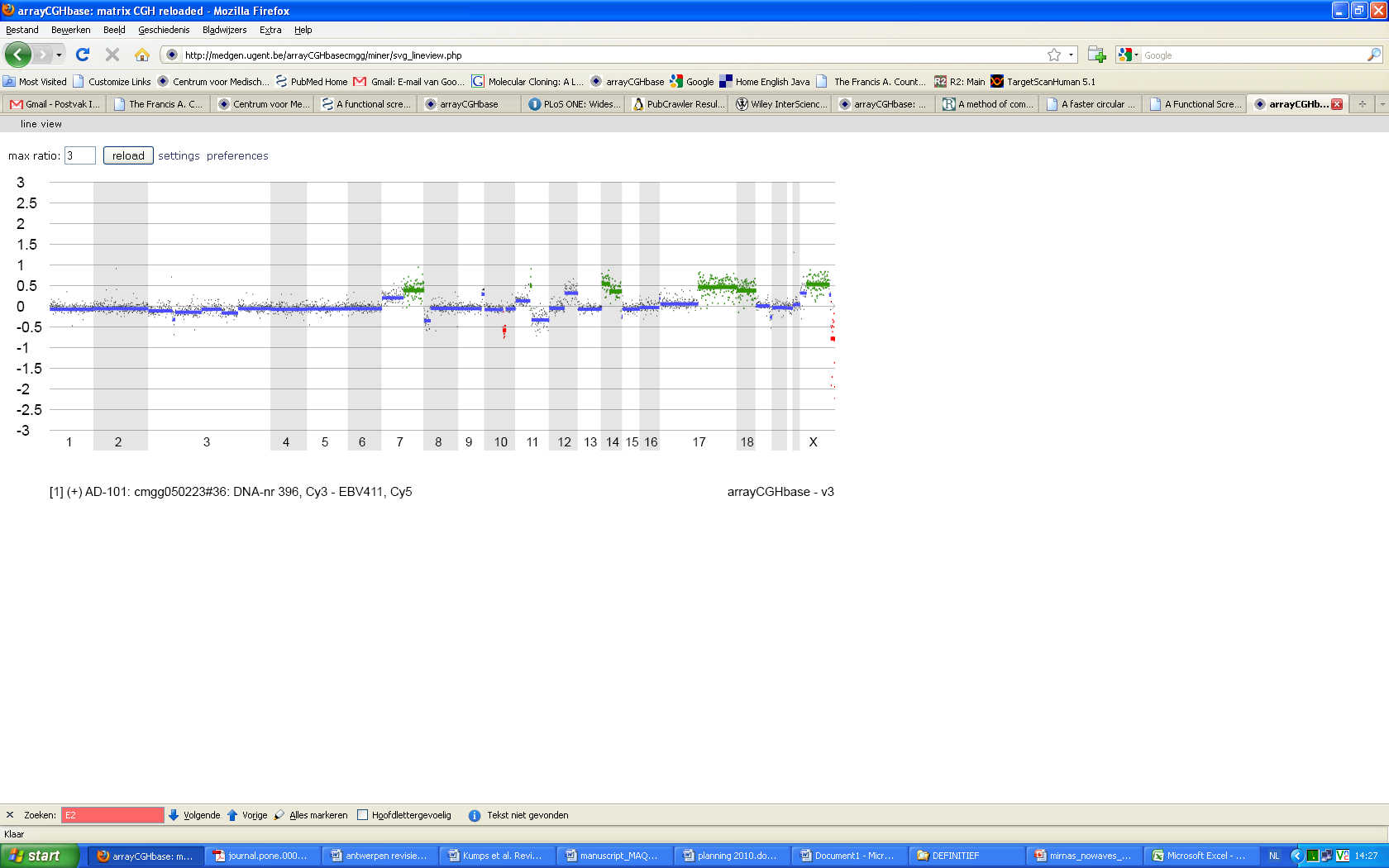
NB_176


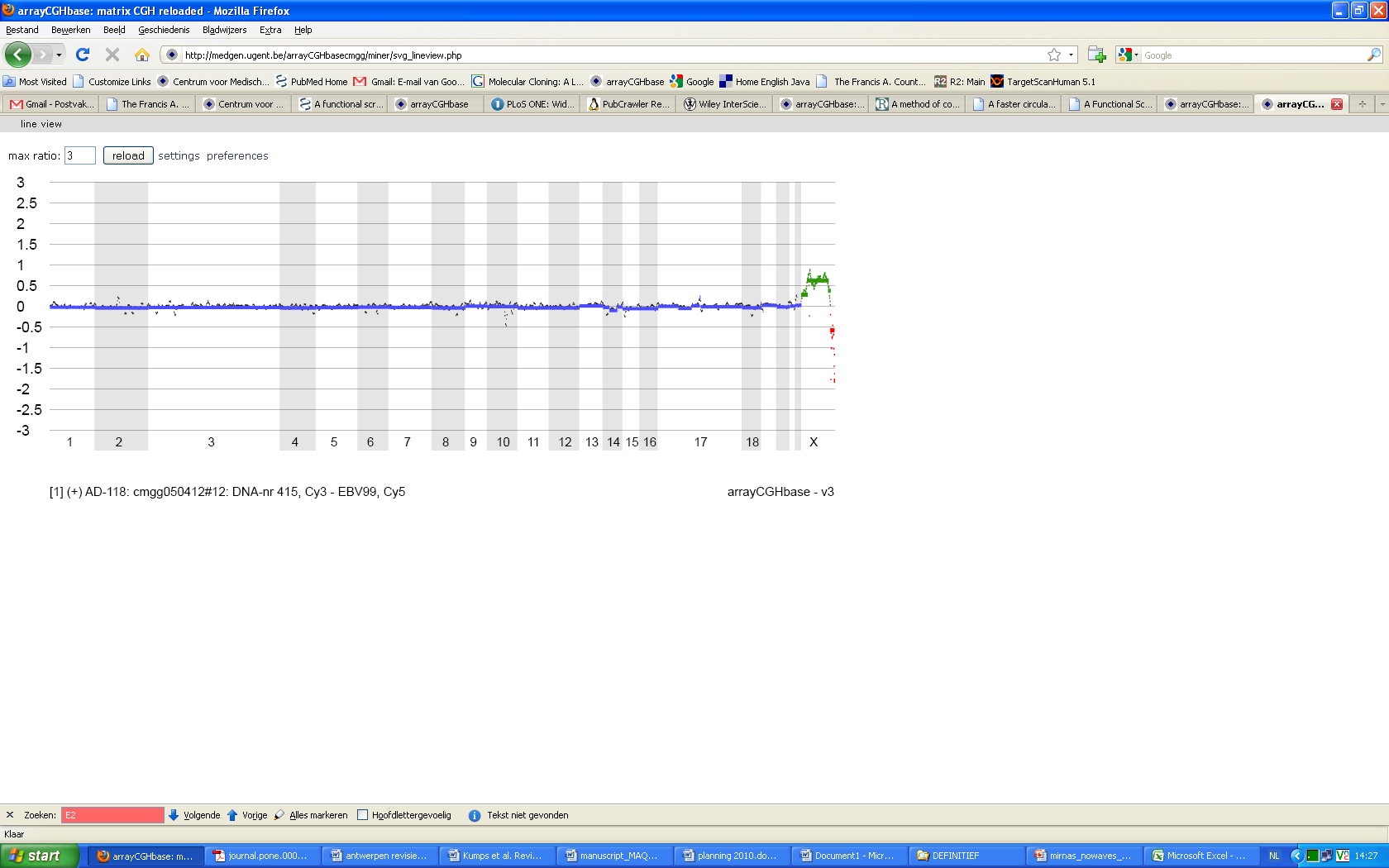
NB_182


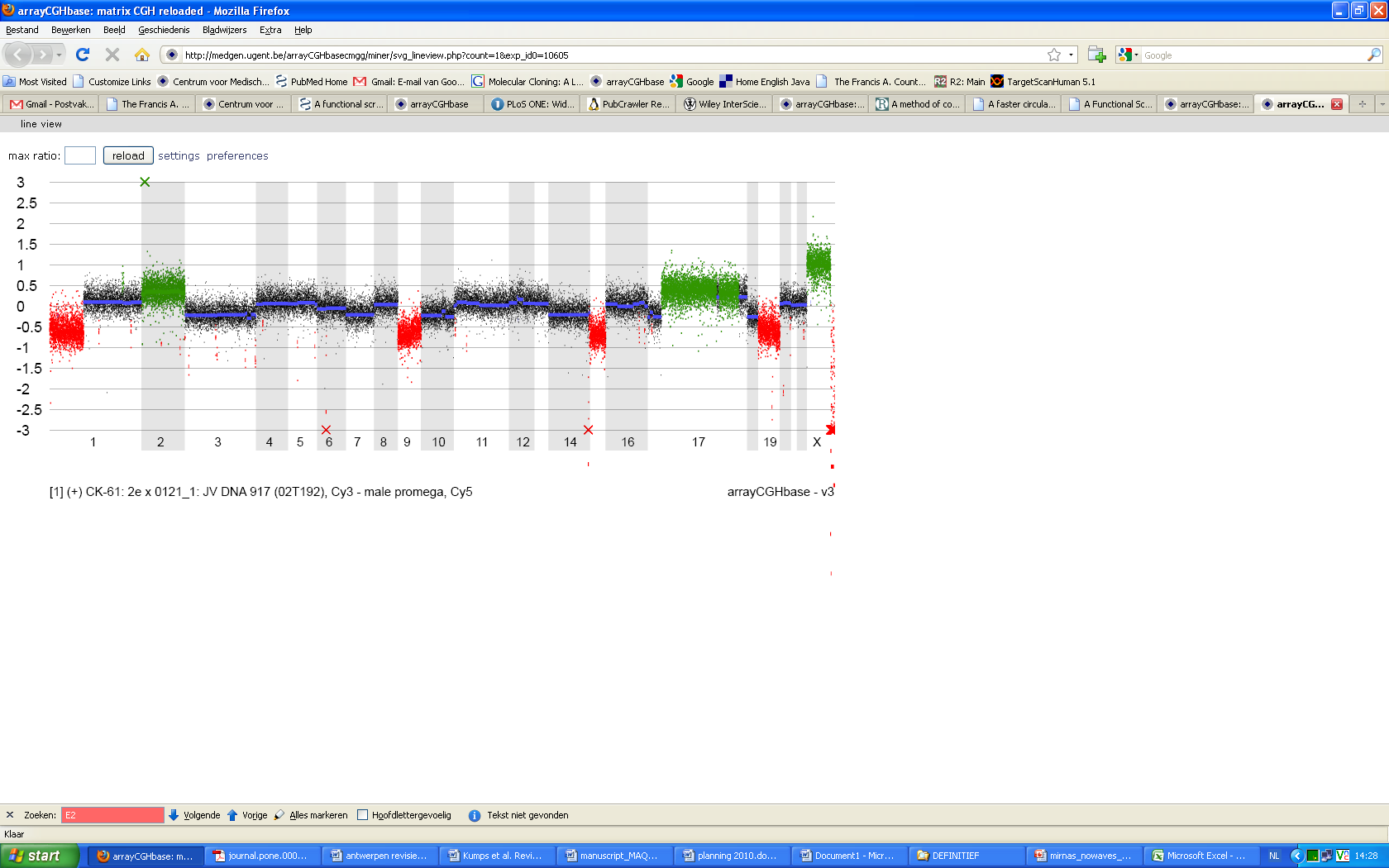
NB_187


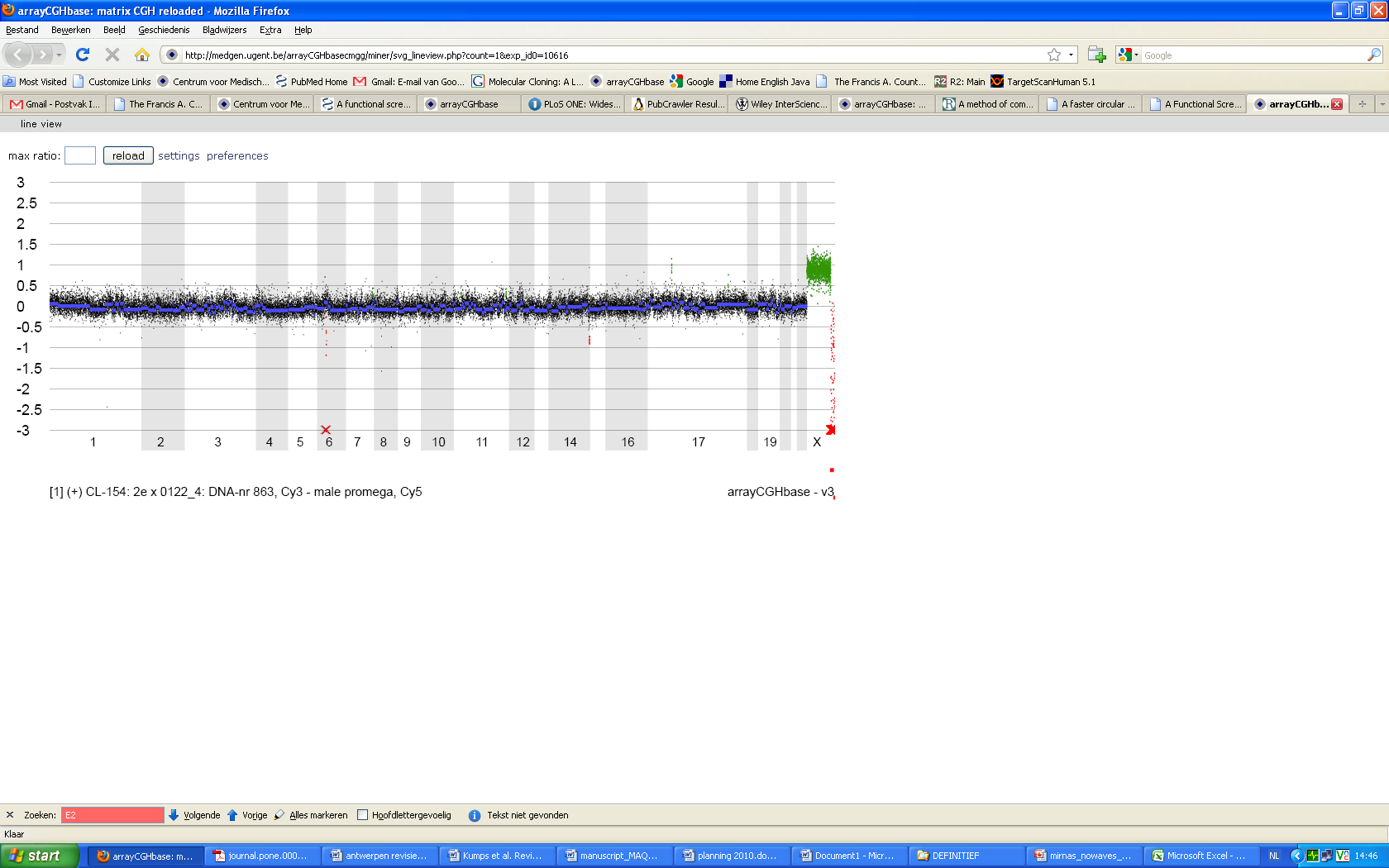
NB_382


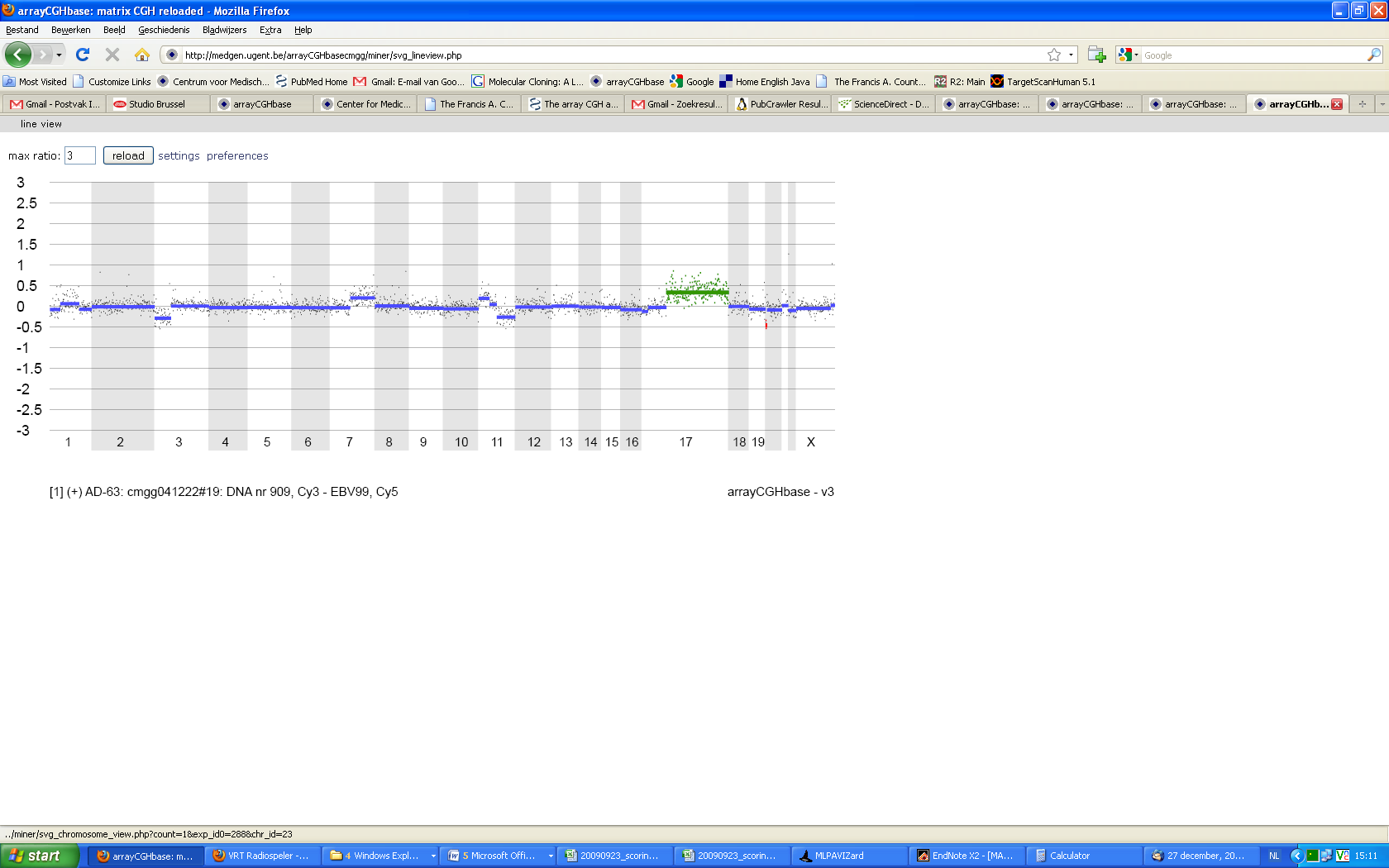
NB_394


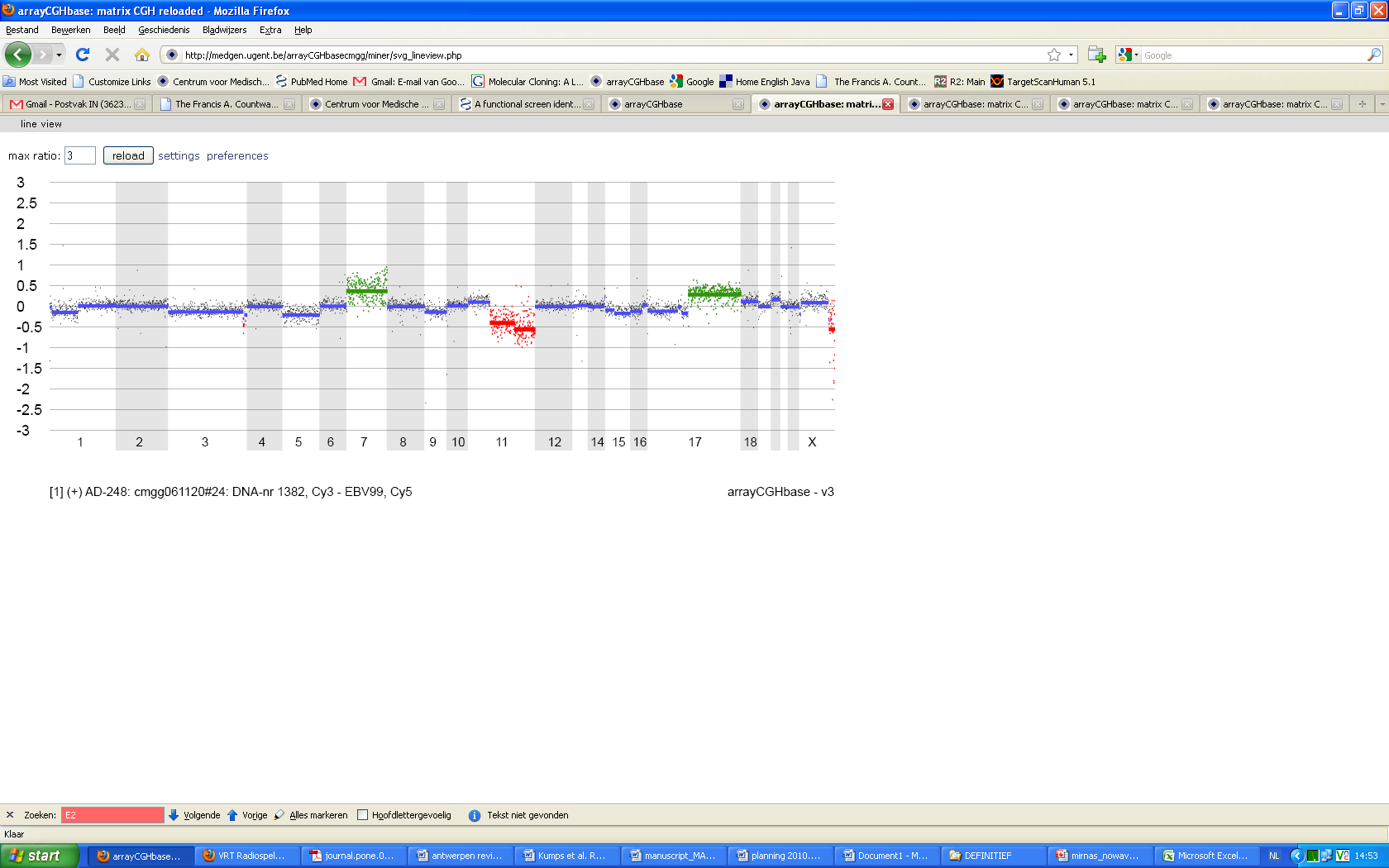
NB_402


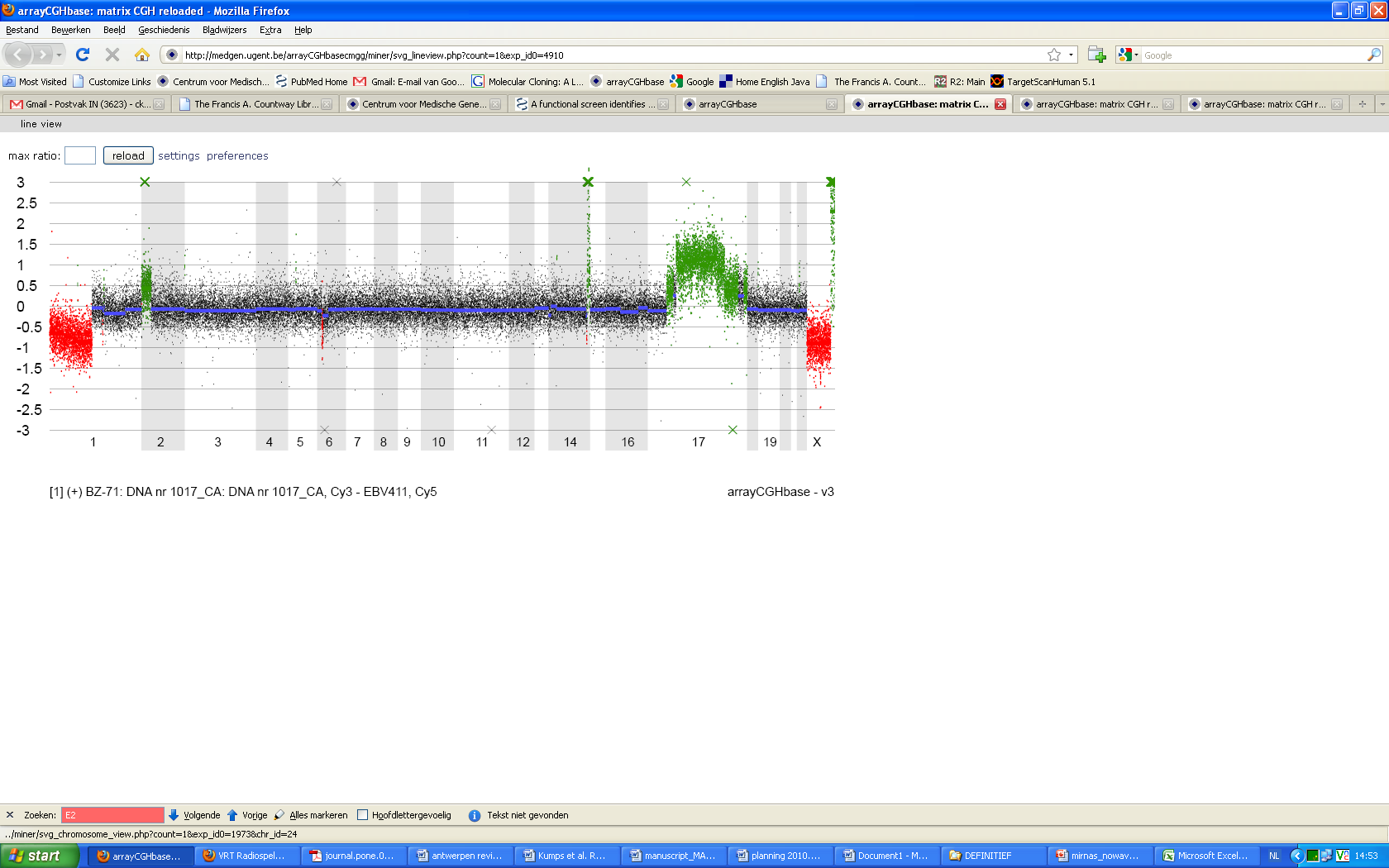
NB_418


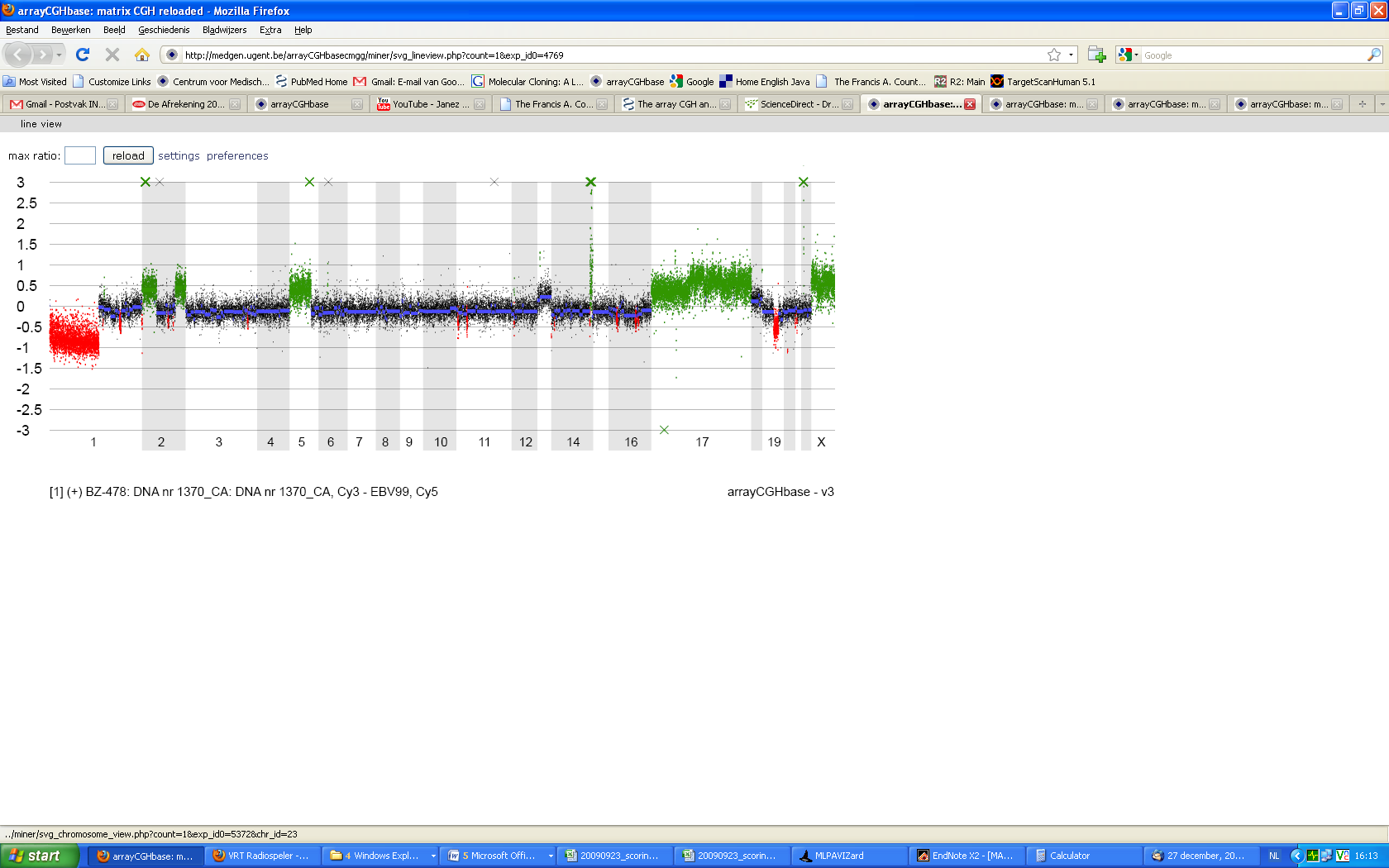
NB_466


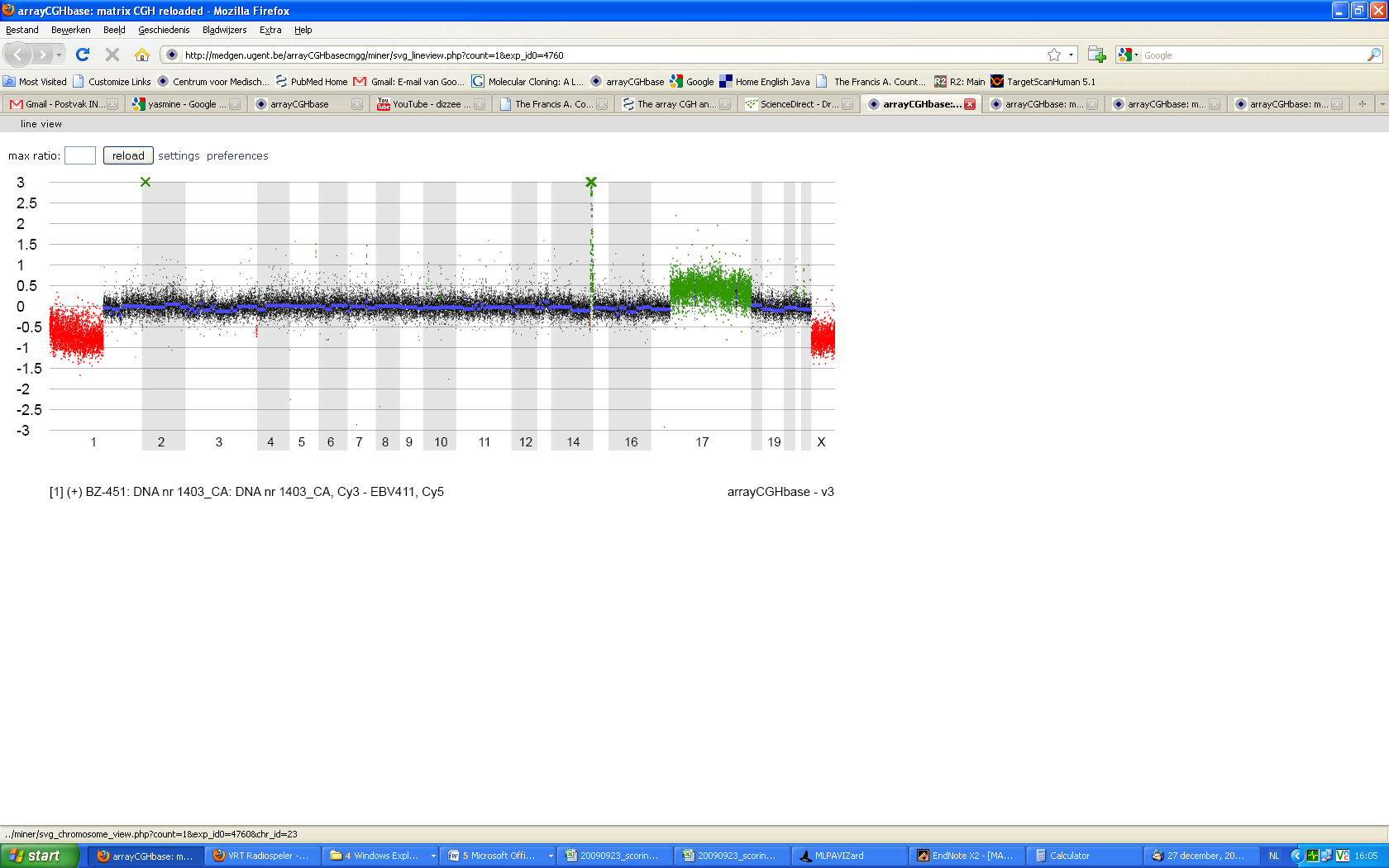
 NB_476


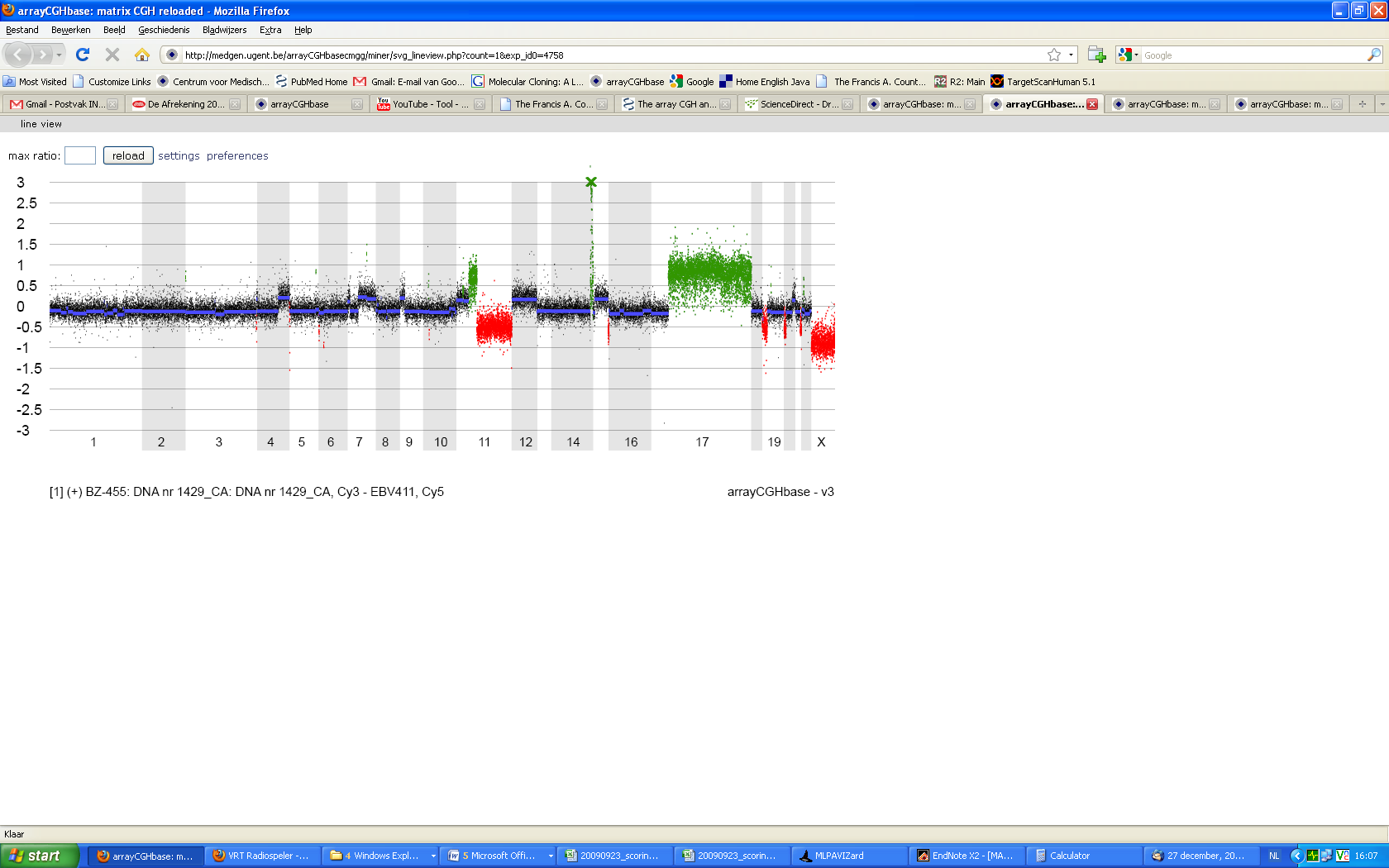
NB_481


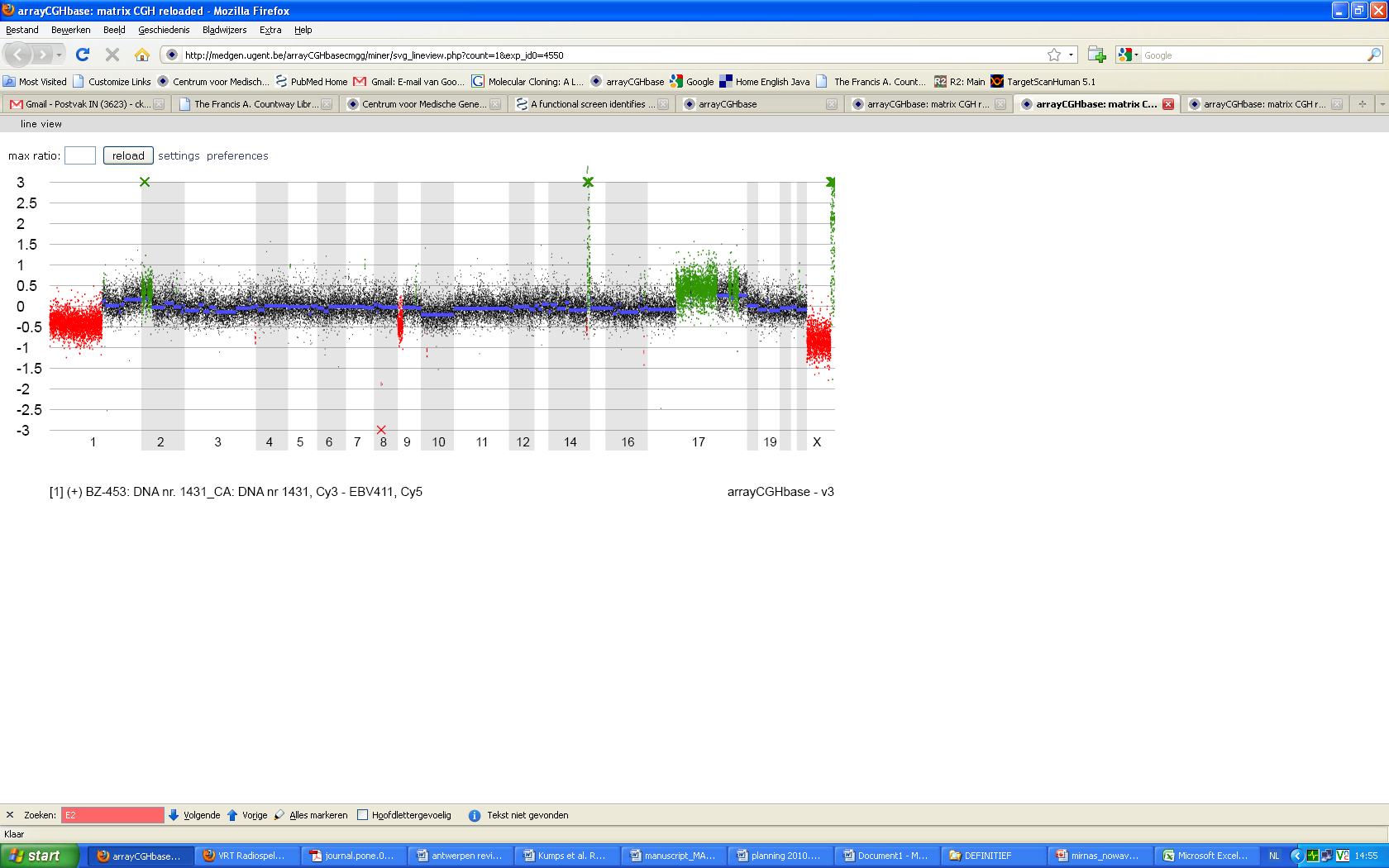
 NB_483


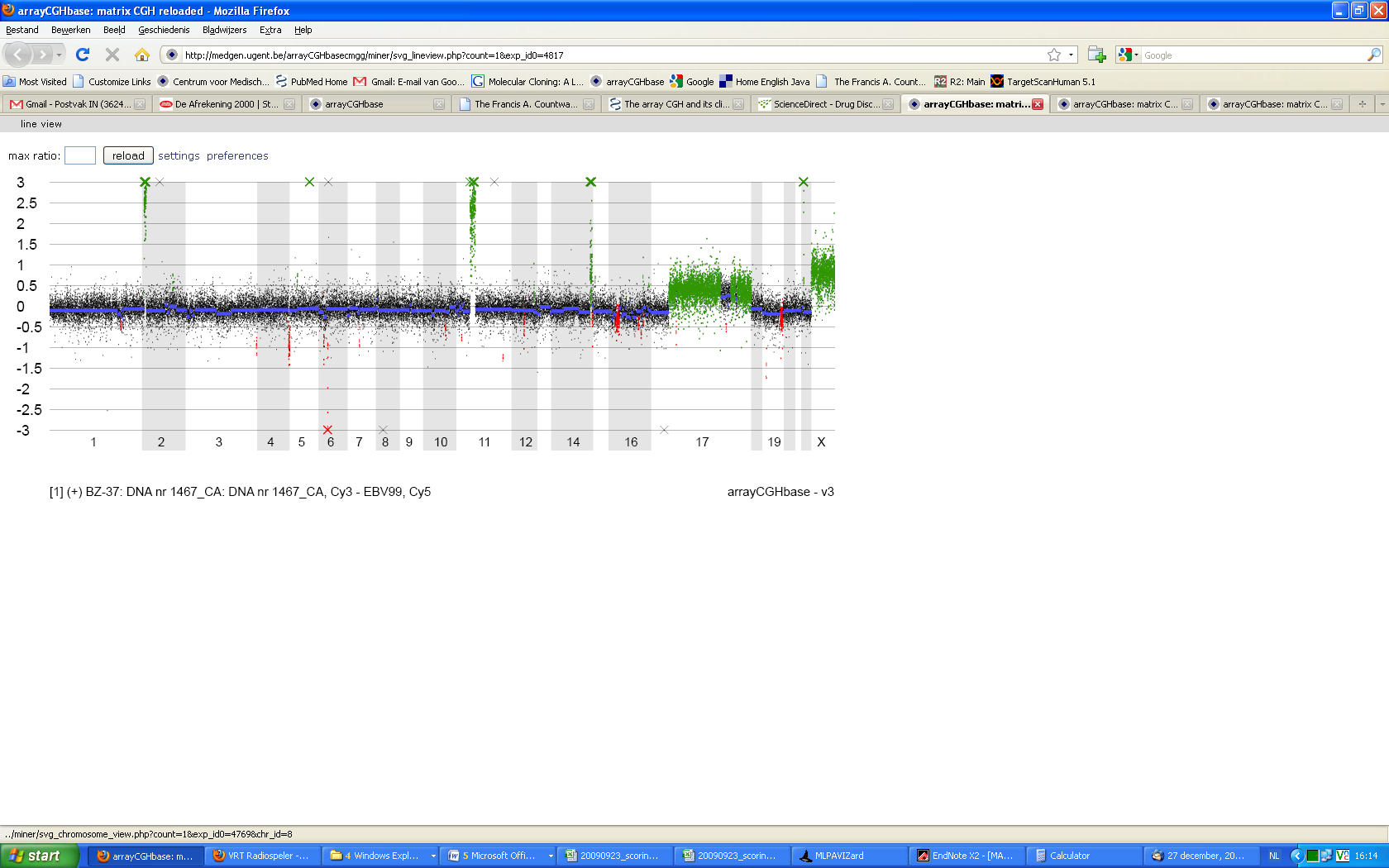
NB_DN_1467


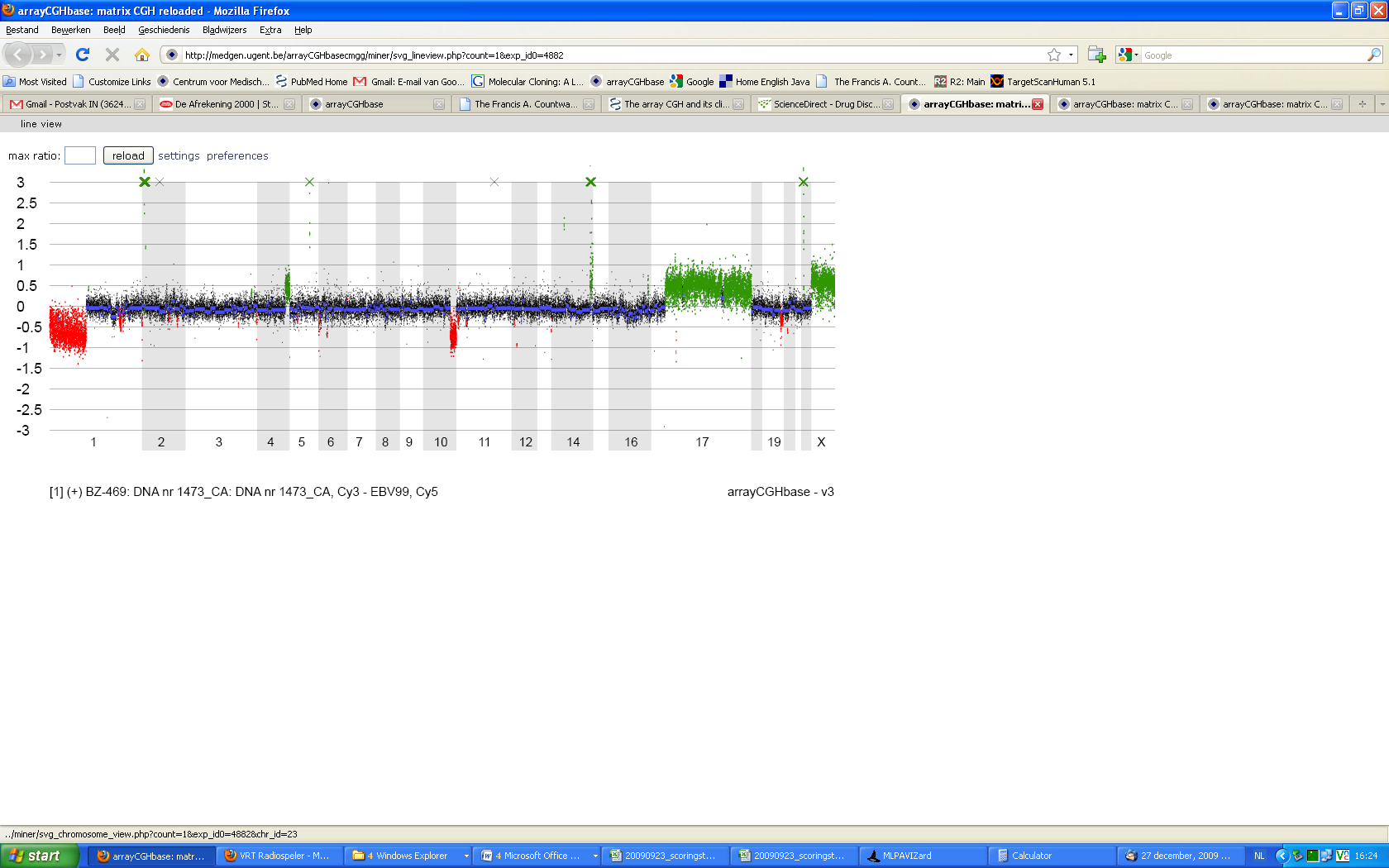
NB_DN_1473


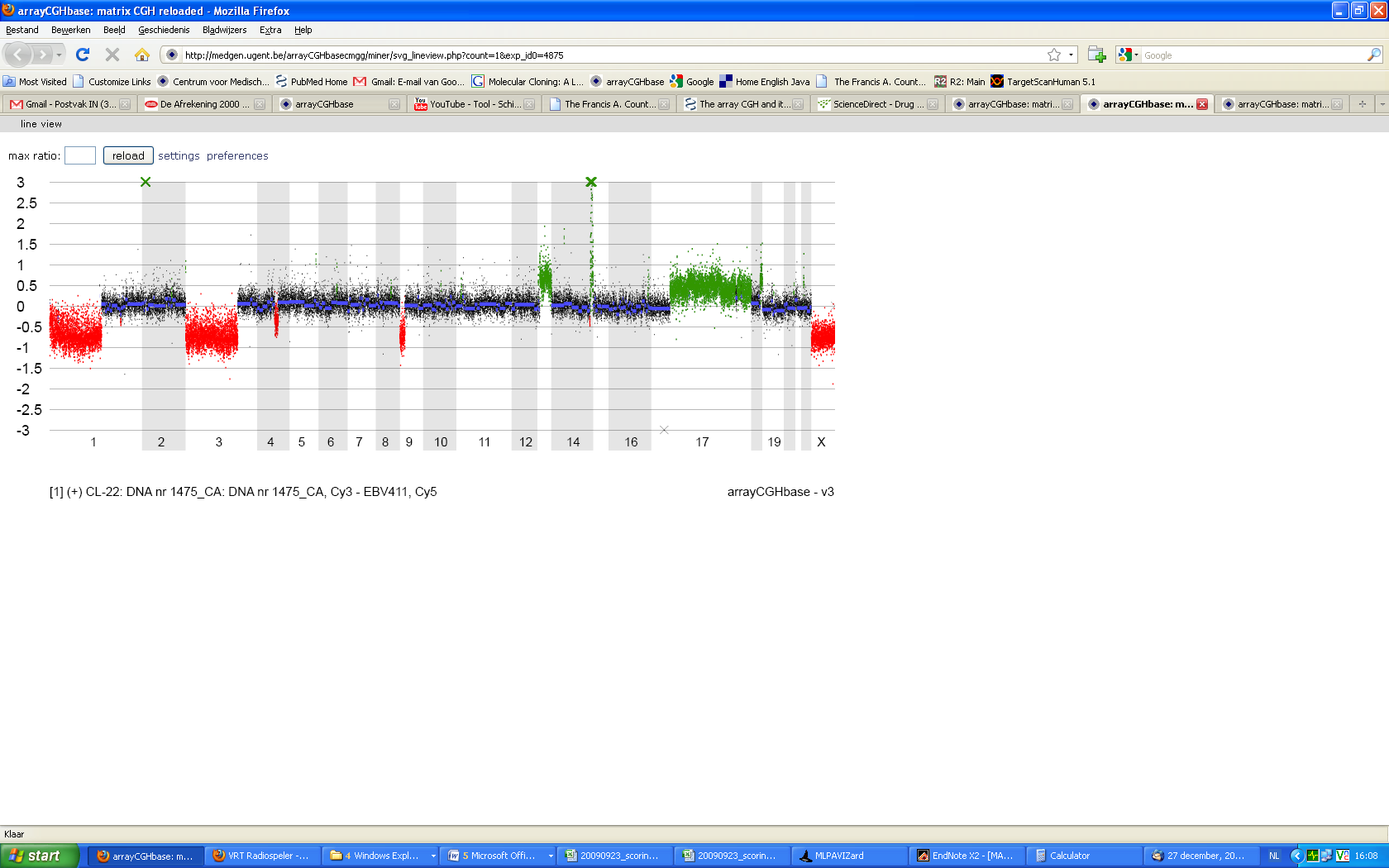
NB_DN_1475


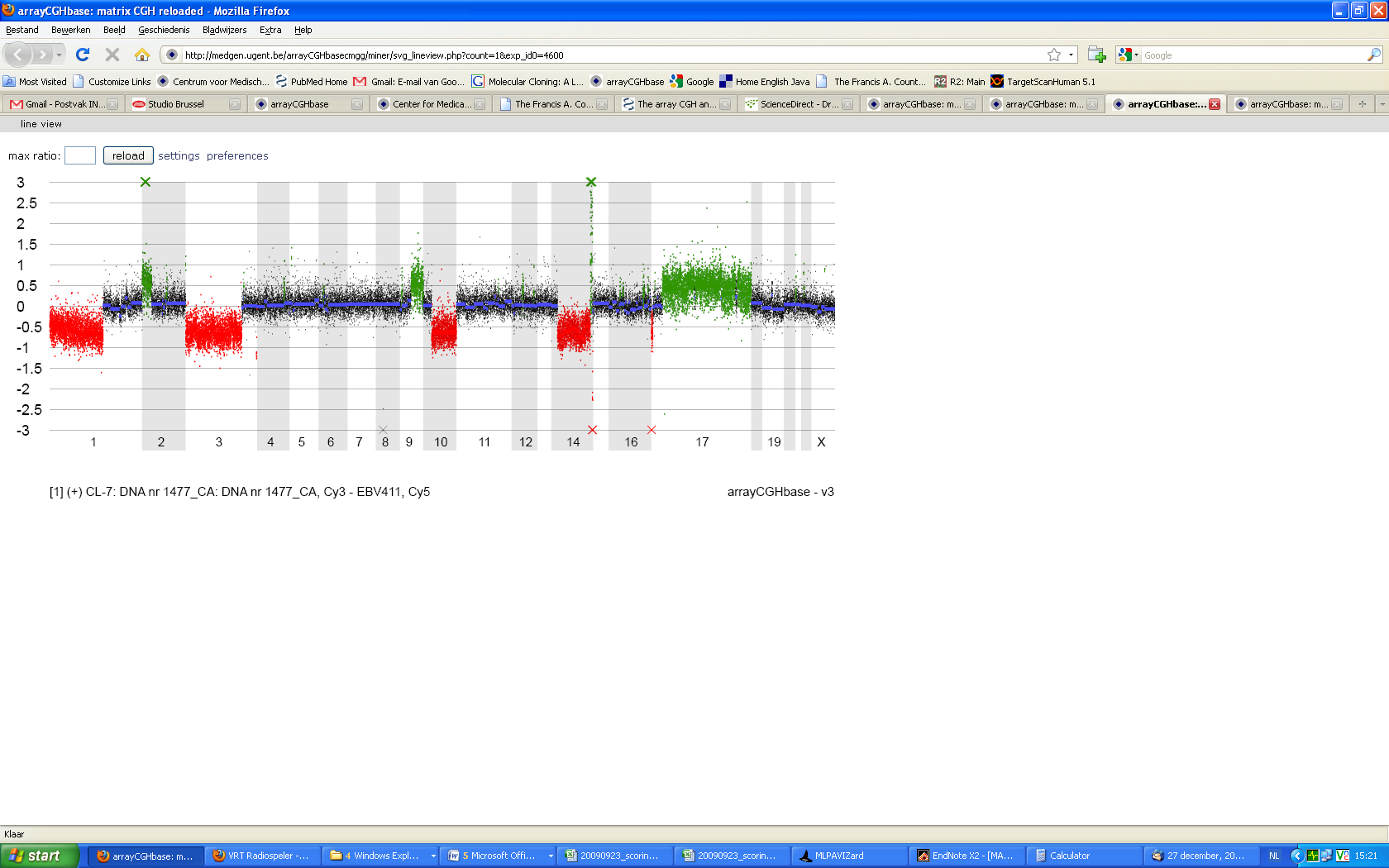
NB_DN_1477


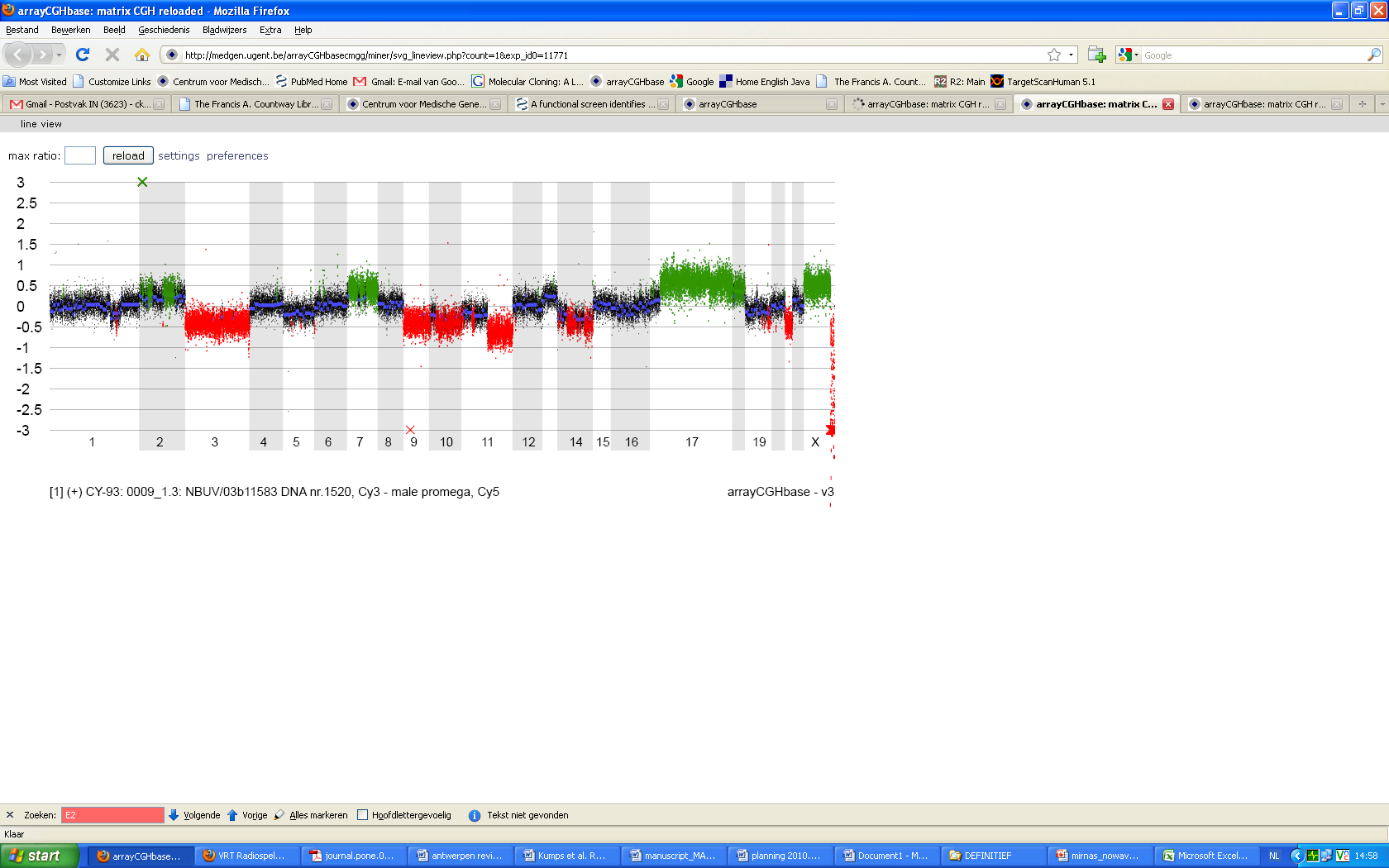
NB_DN_1520


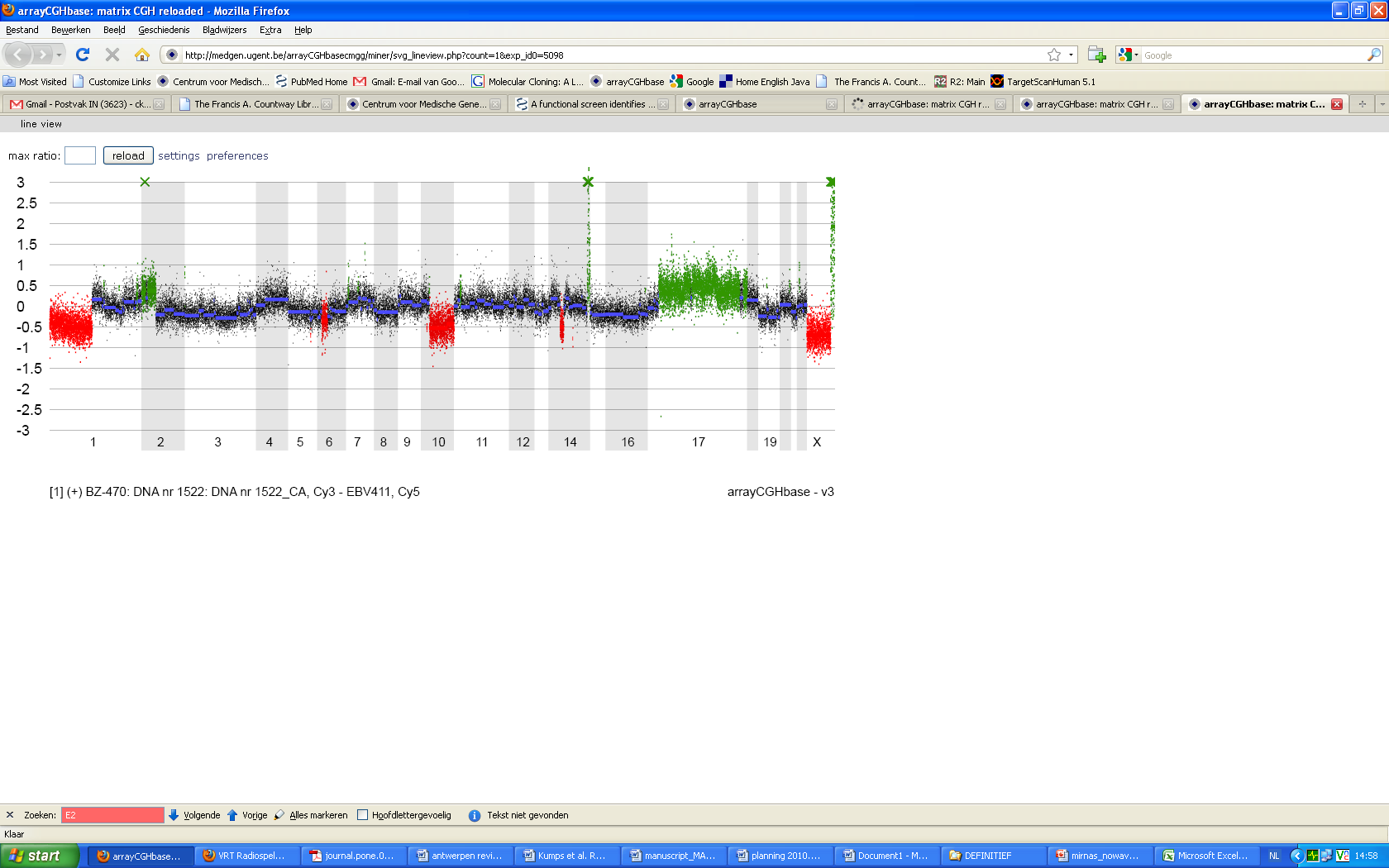
NB_DN_1522


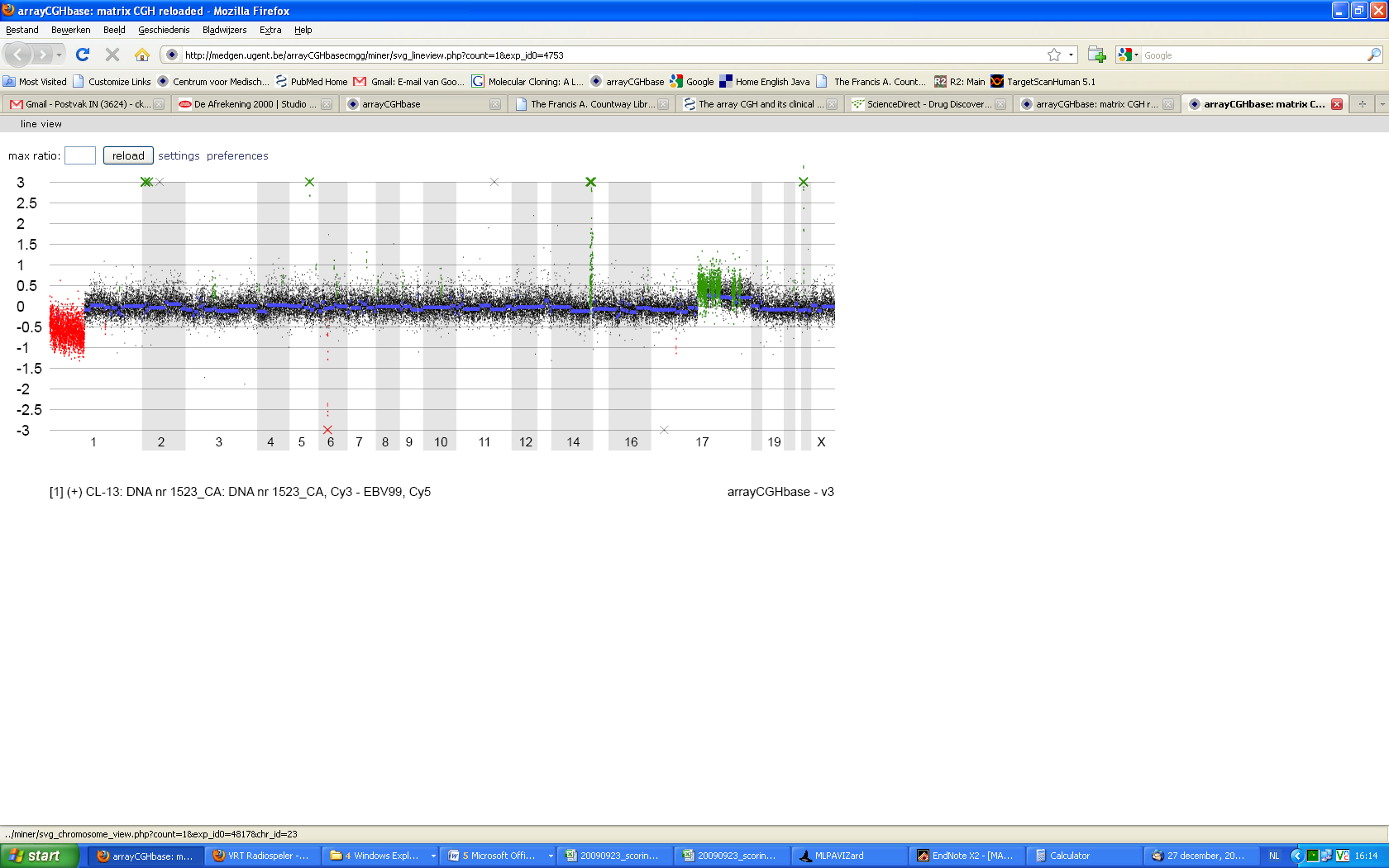
NB_DN_1523


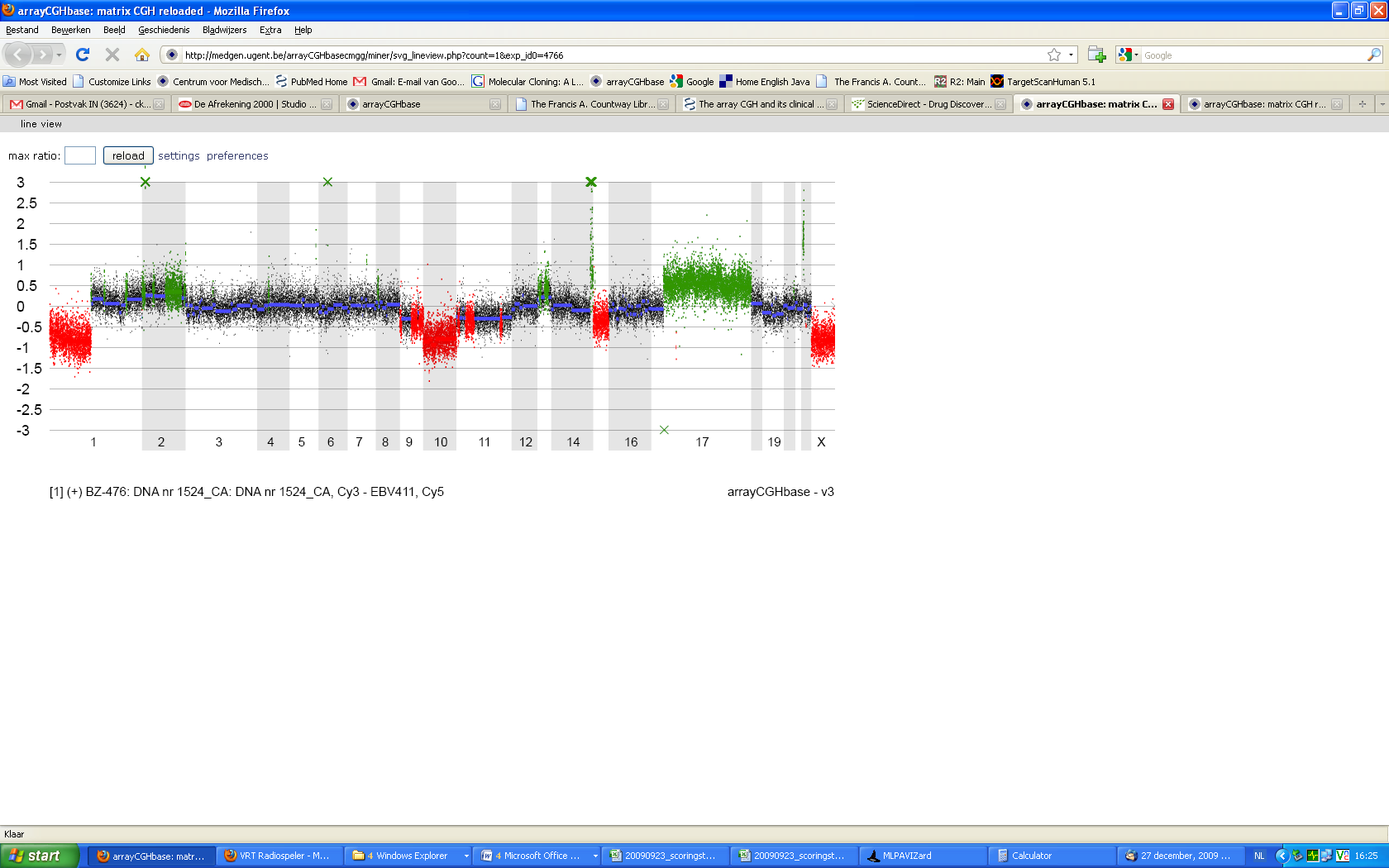
NB_DN_1524


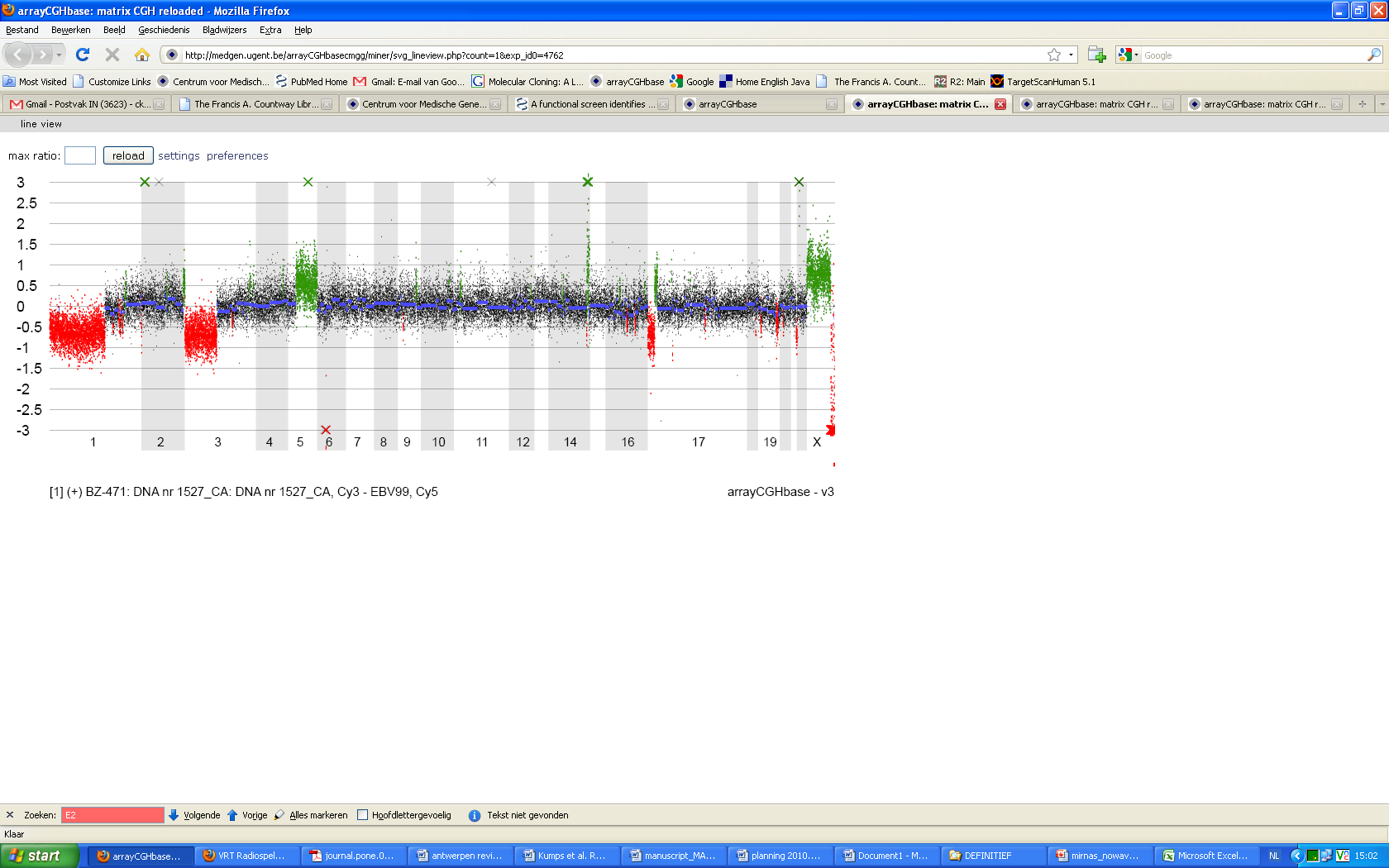
NB_DN_1527
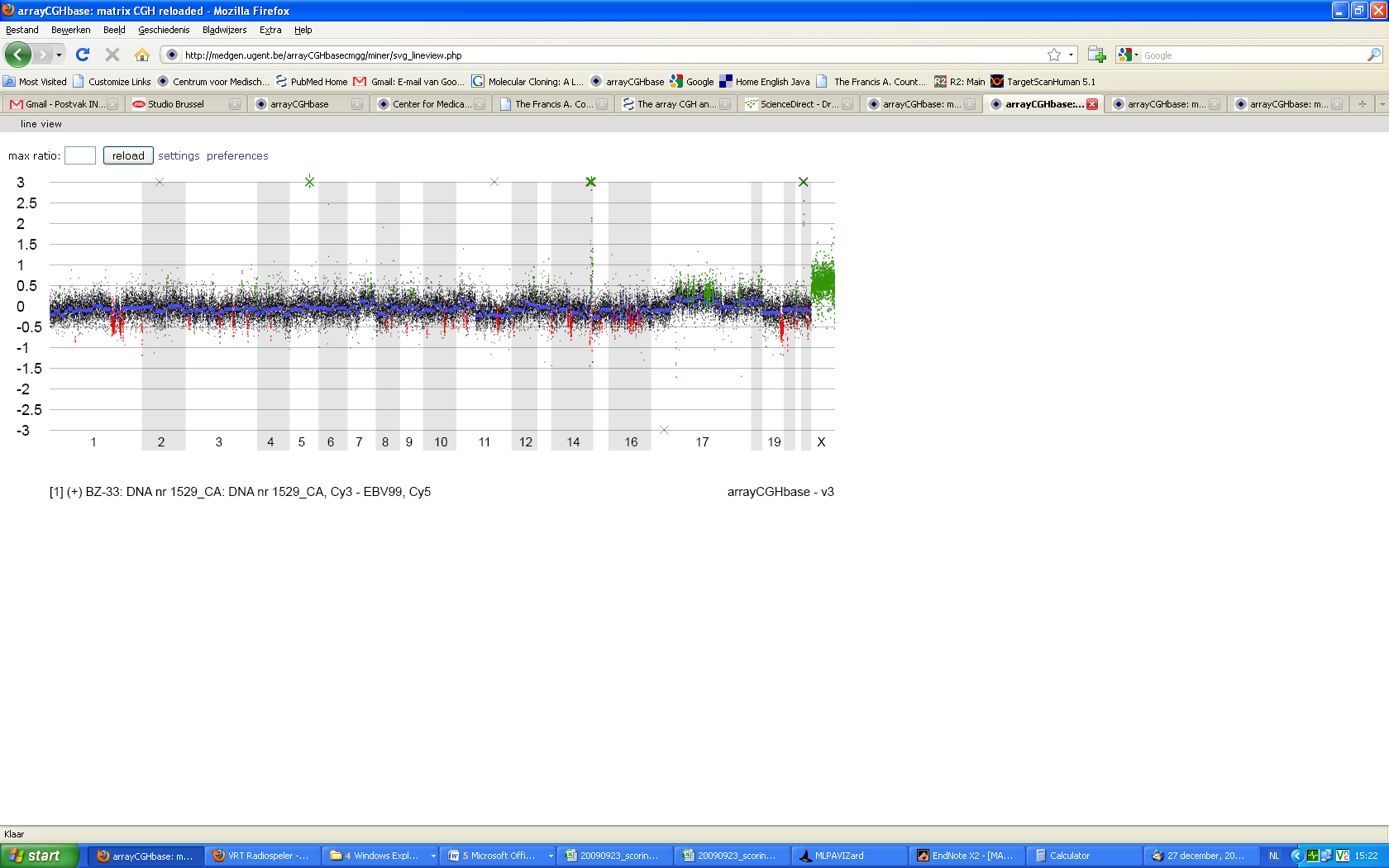
 NB_DN_1529


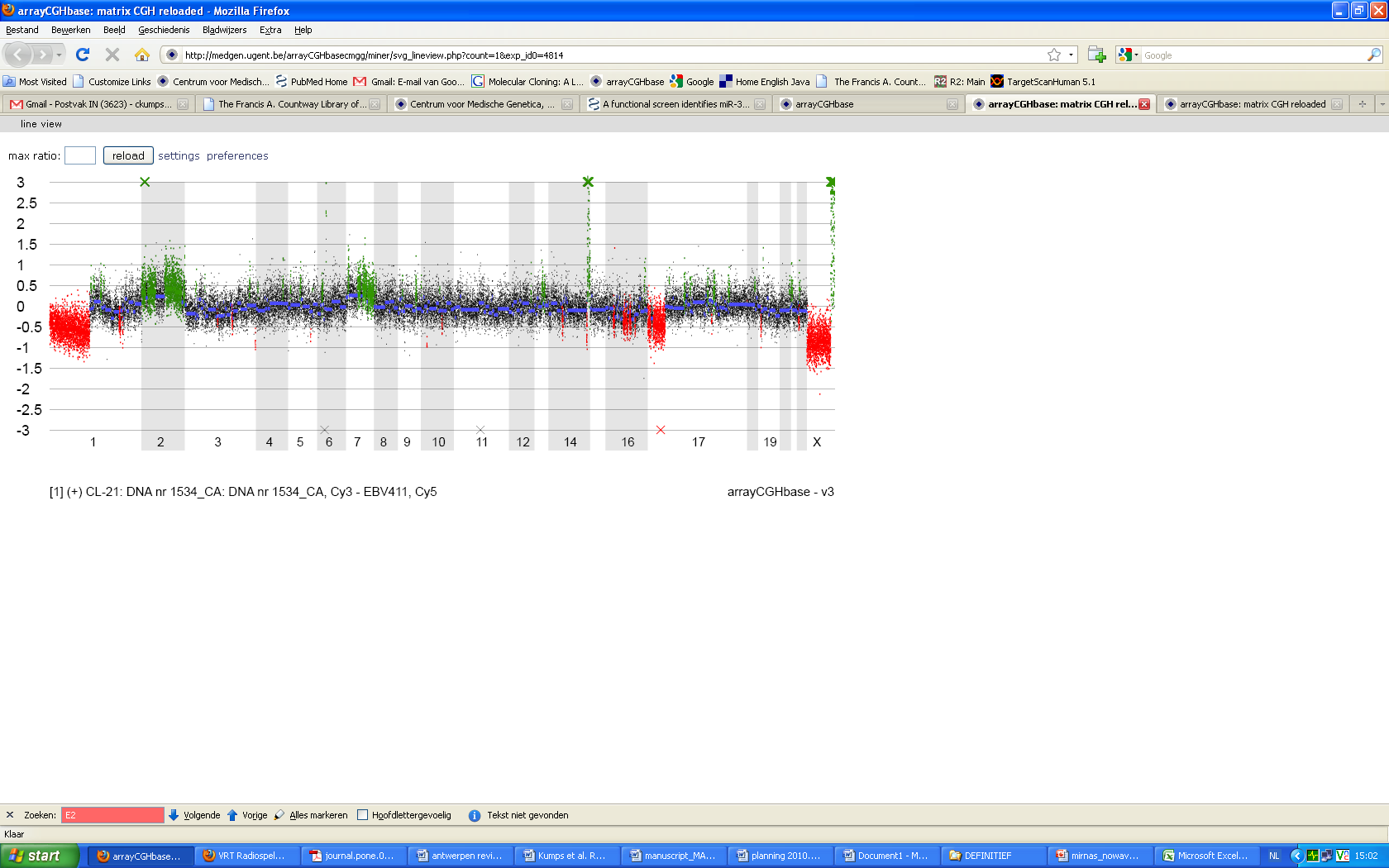
NB_DN_1534


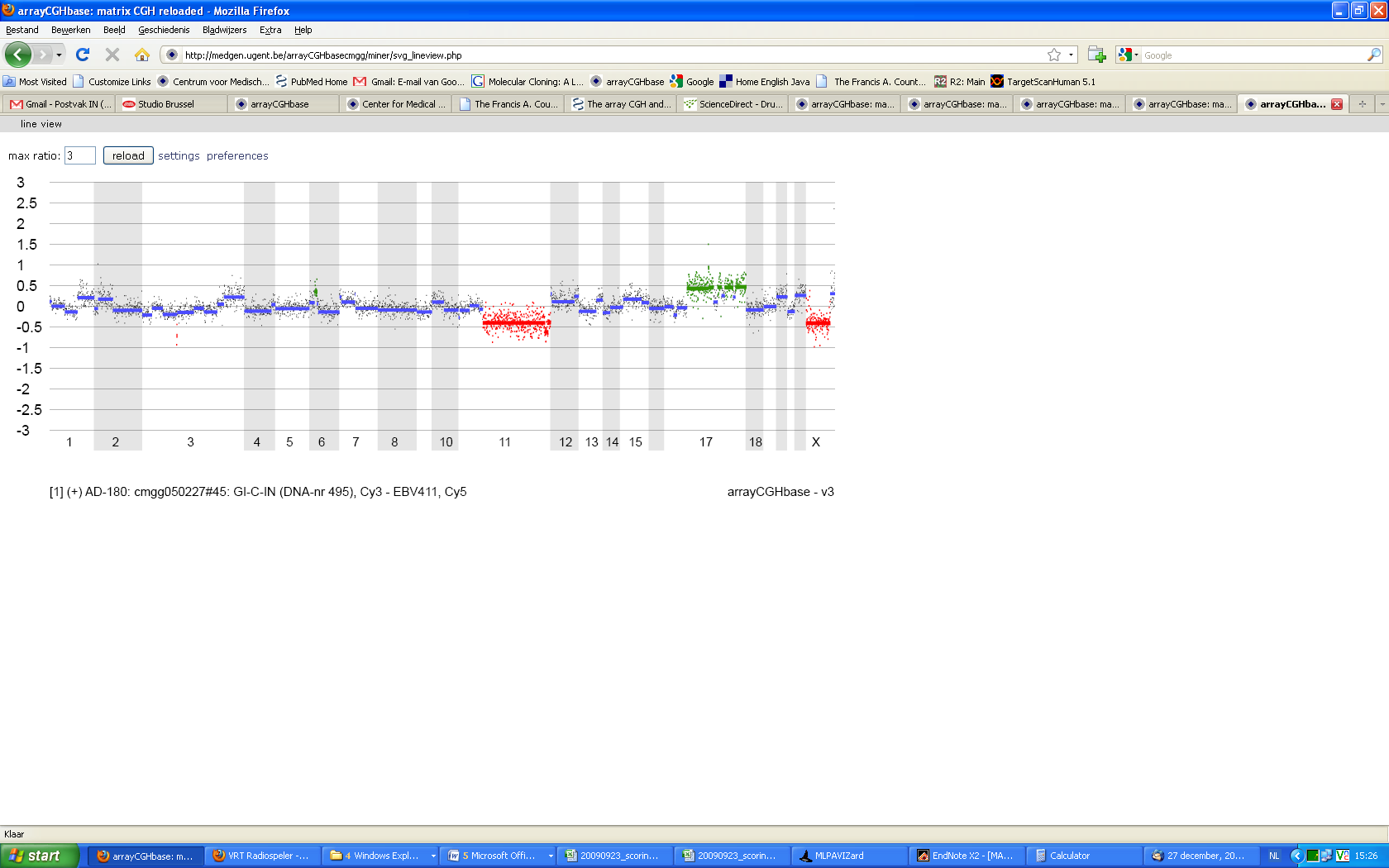
 GI-C-IN


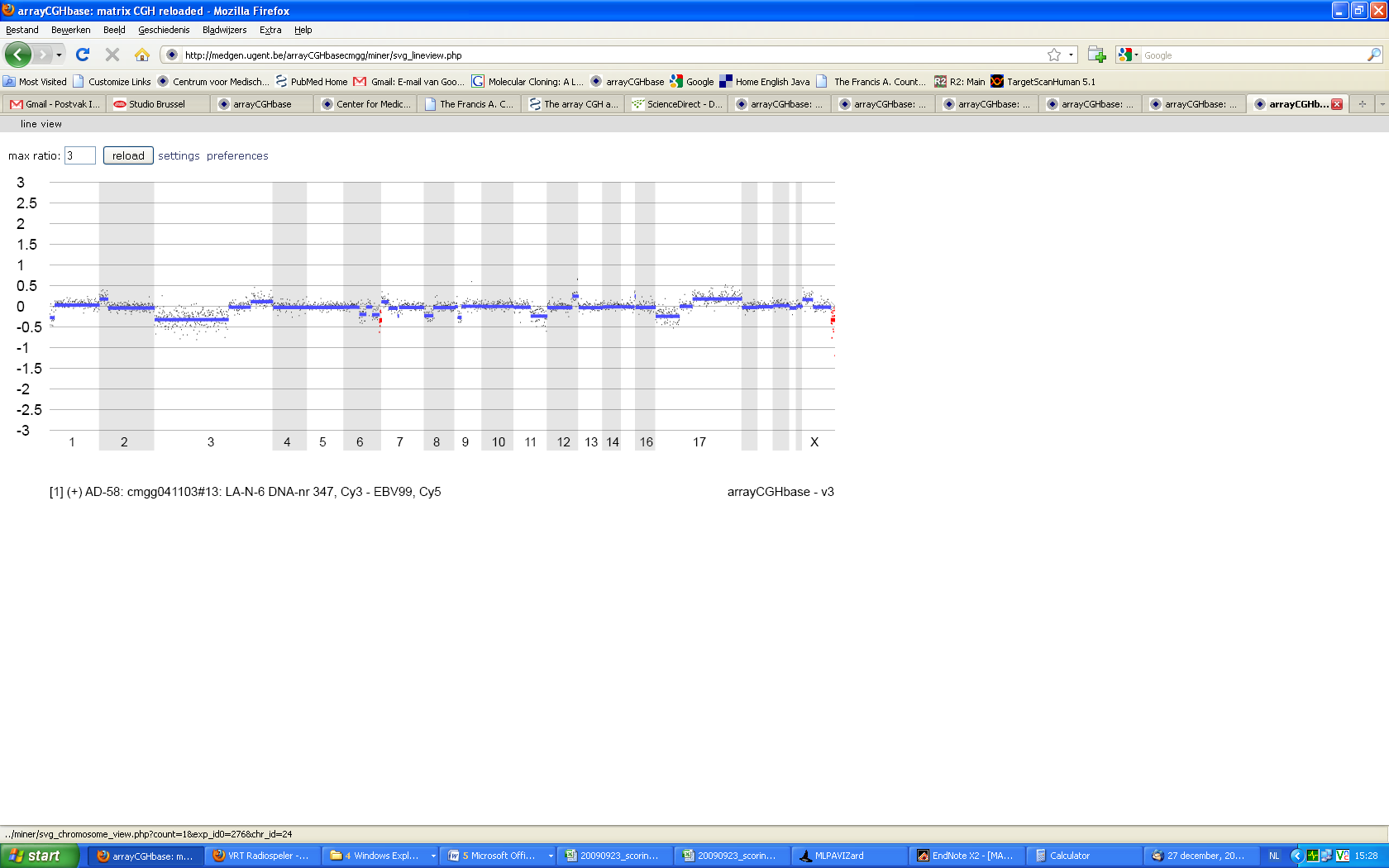
 LAN-6


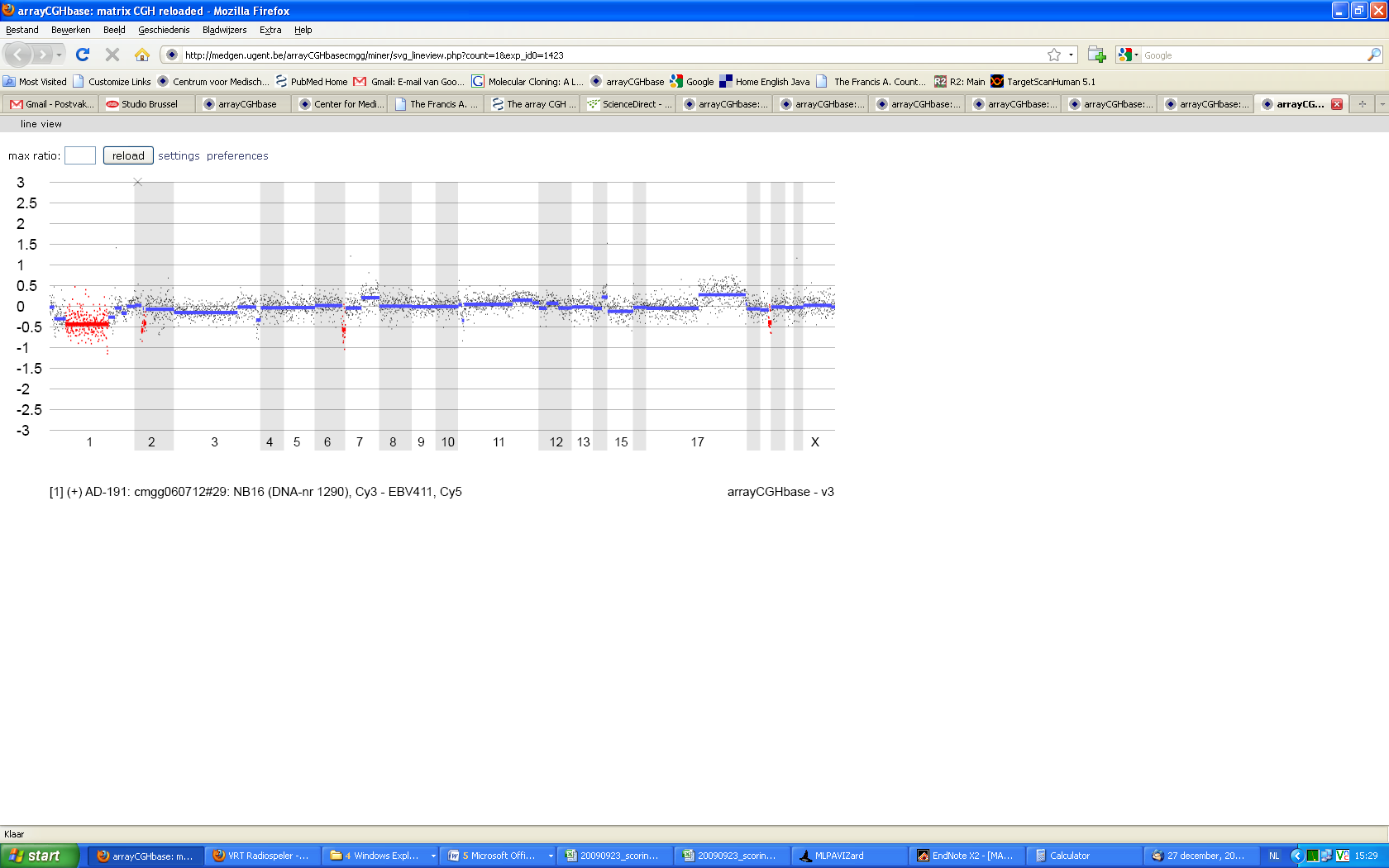
 NB-16


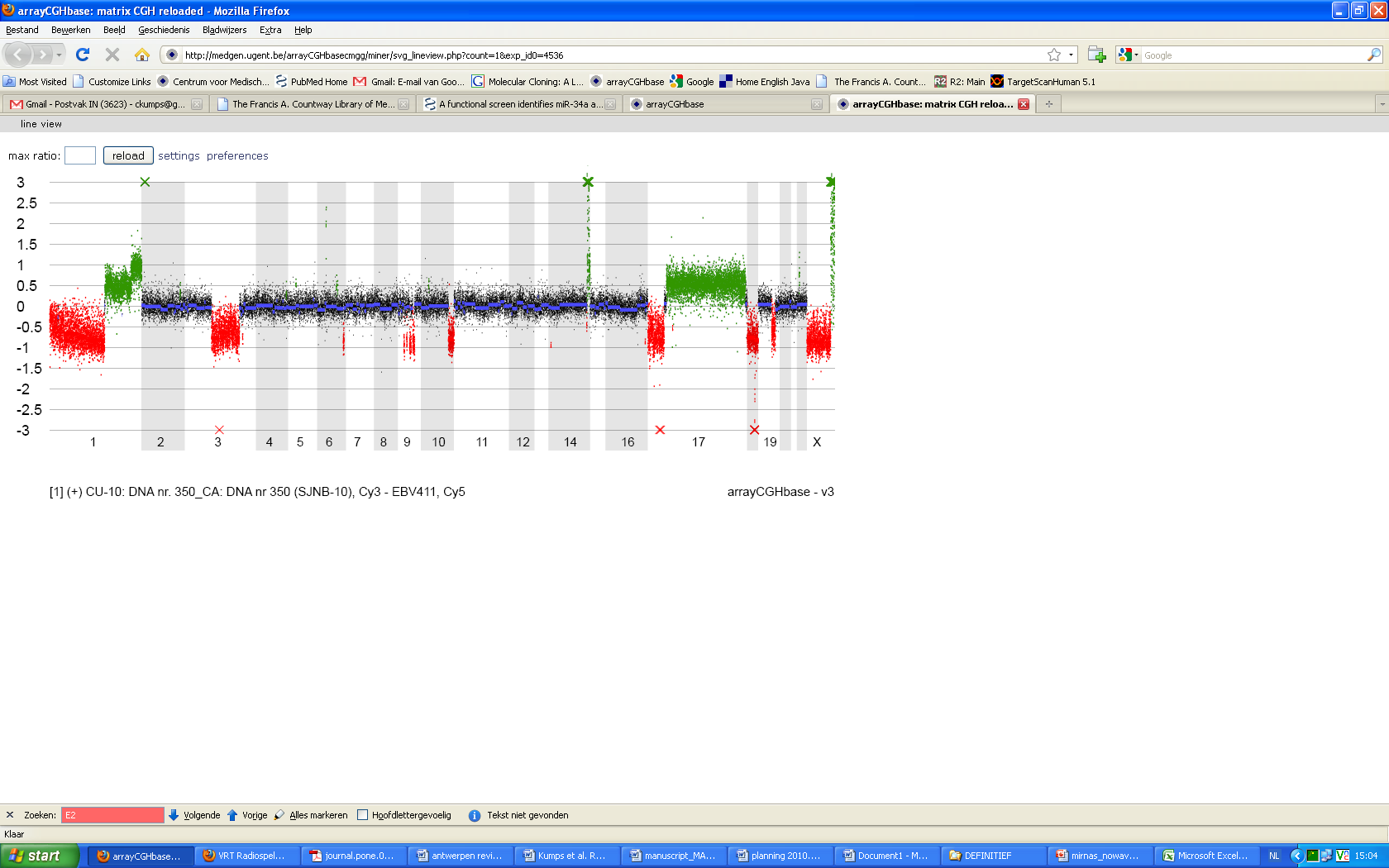
SJNB-10

Triploid


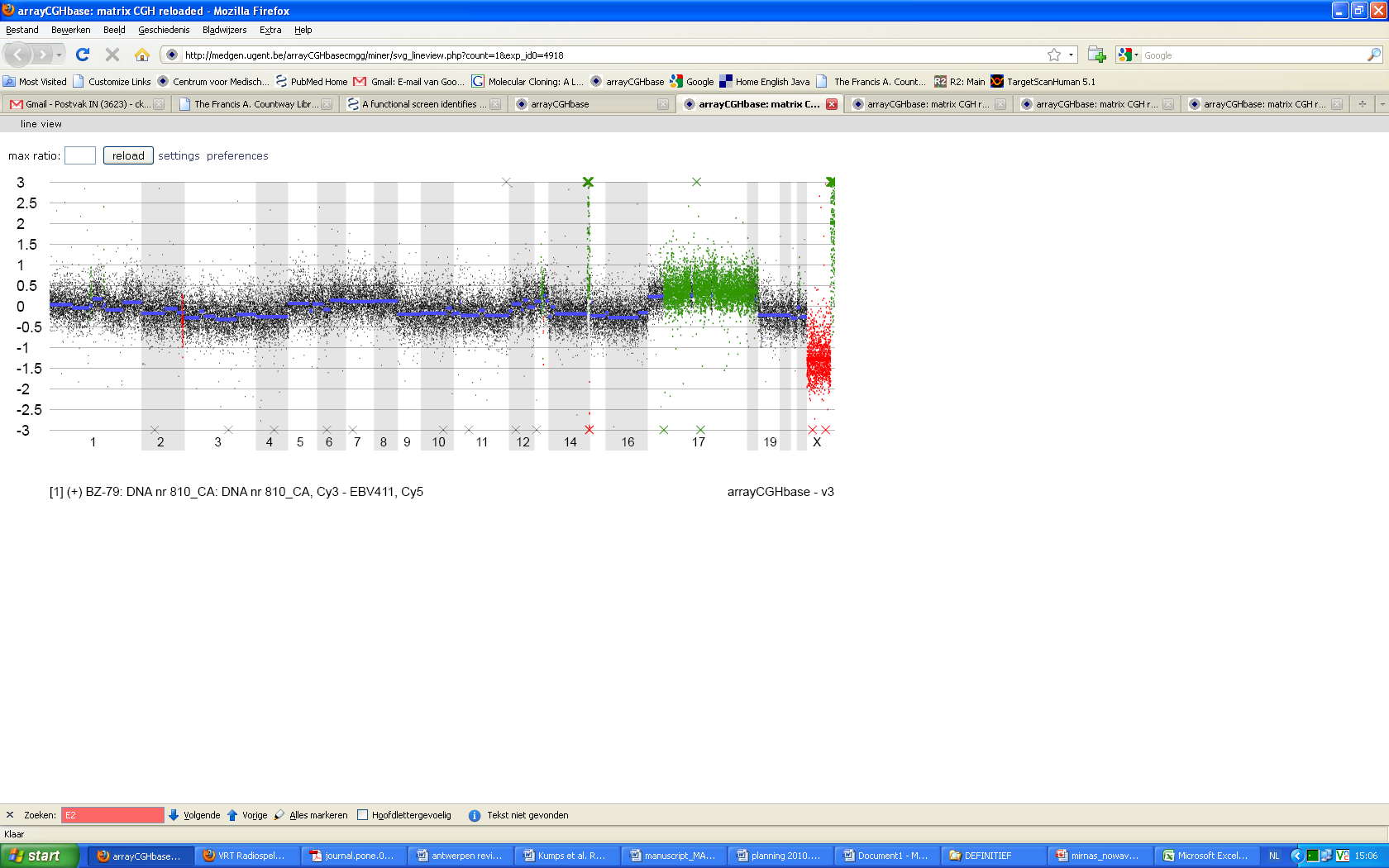
NB_19
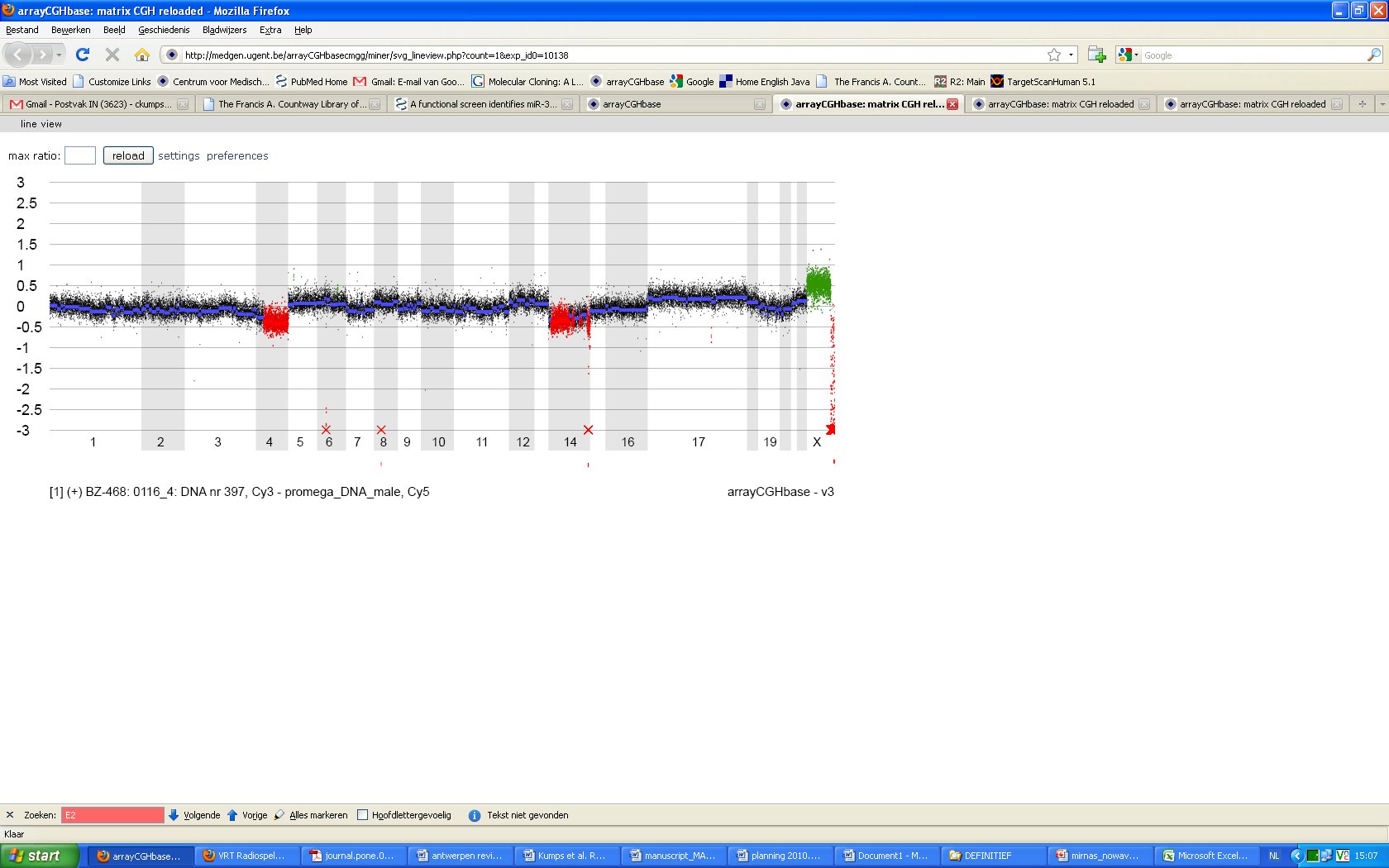
 NB_26


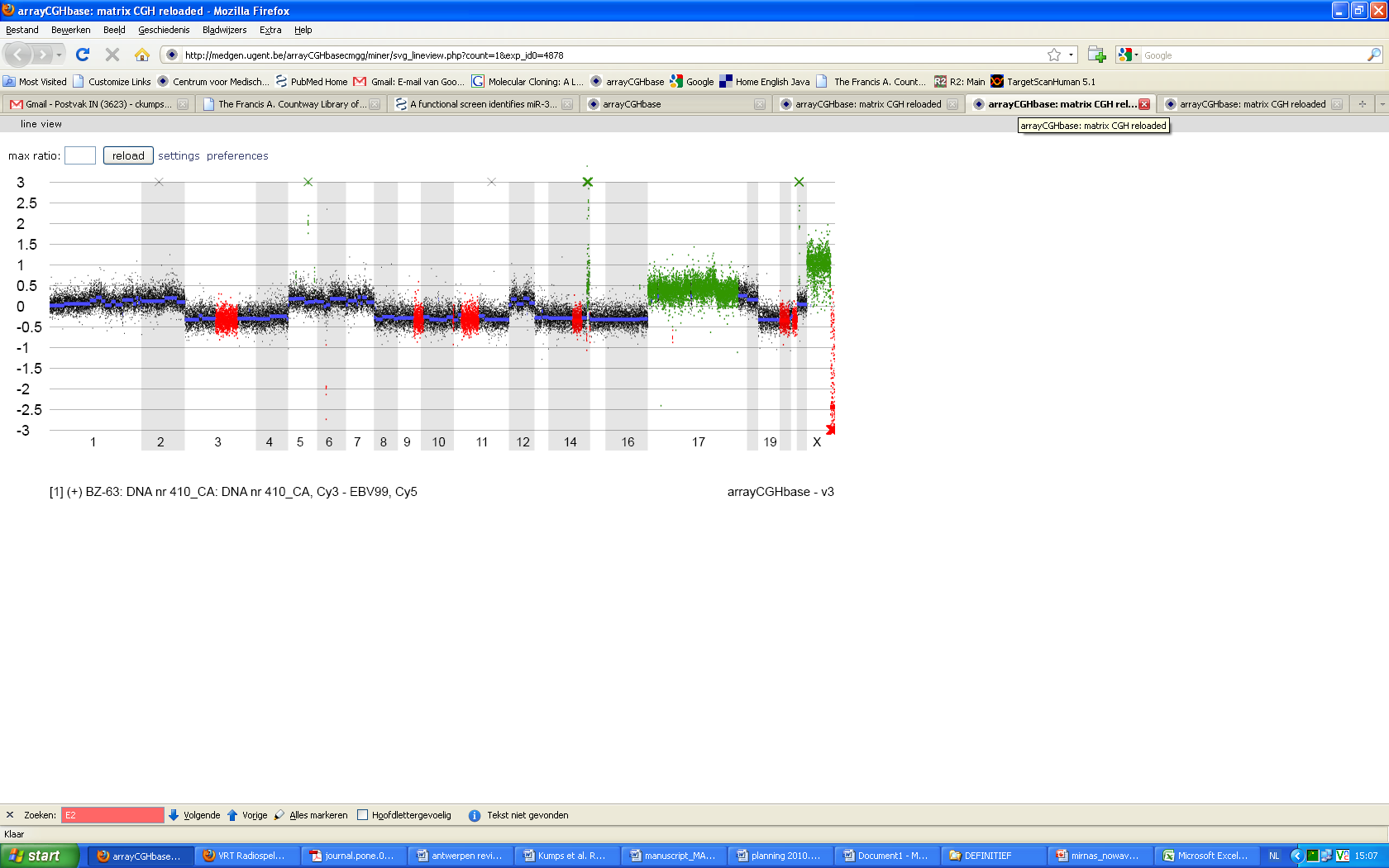
 NB_35


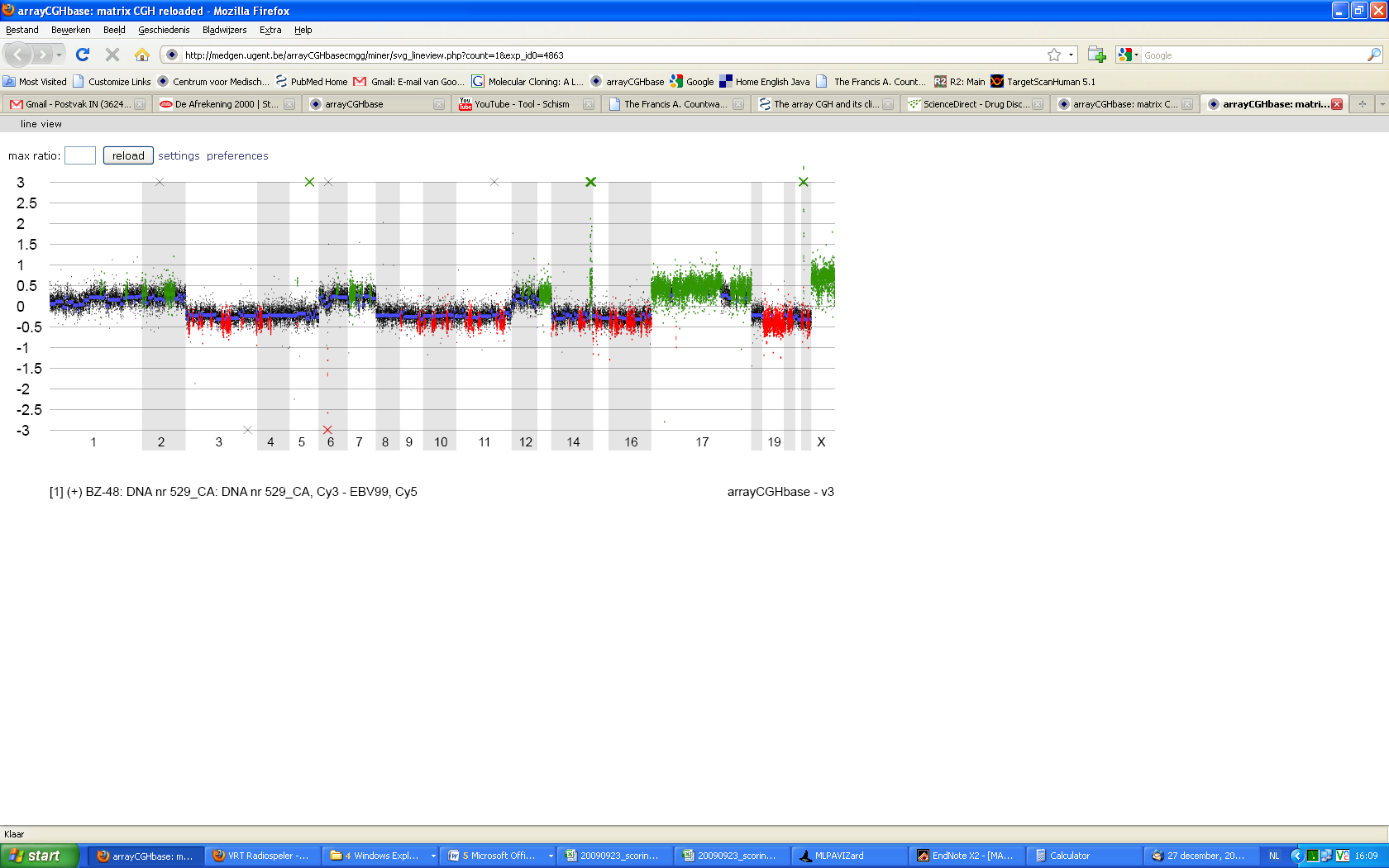
 NB_104


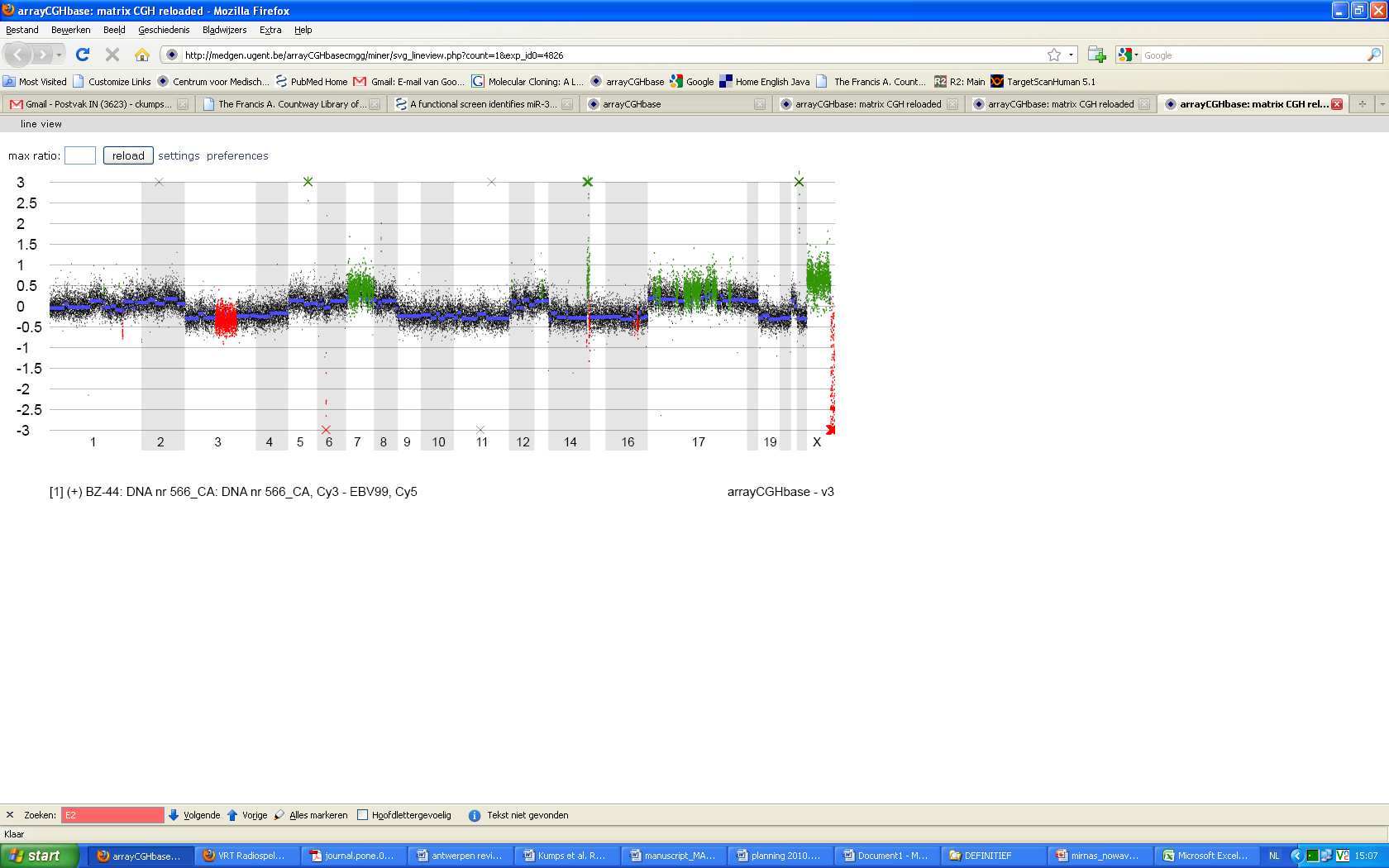
 NB_88


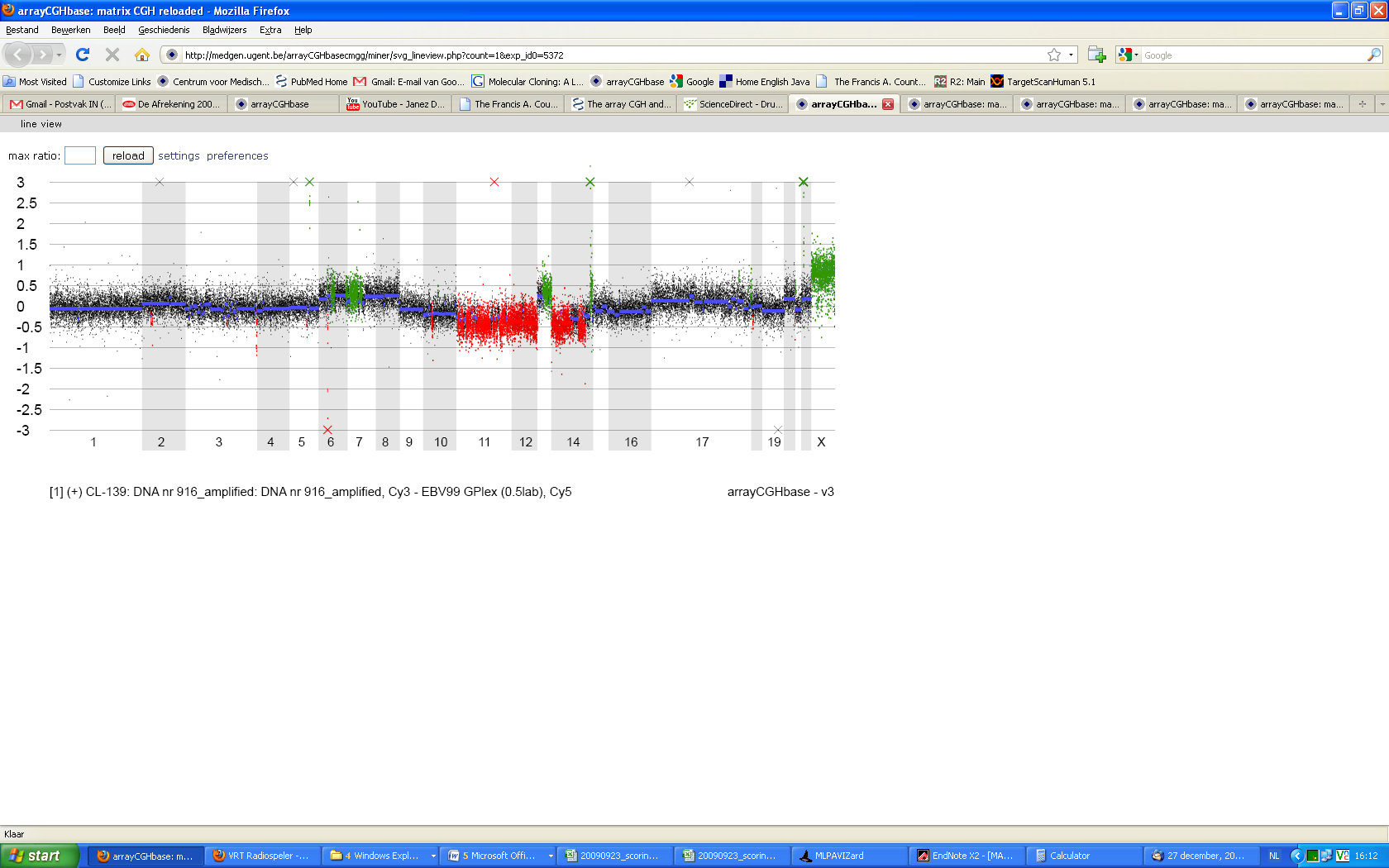
 NB_101


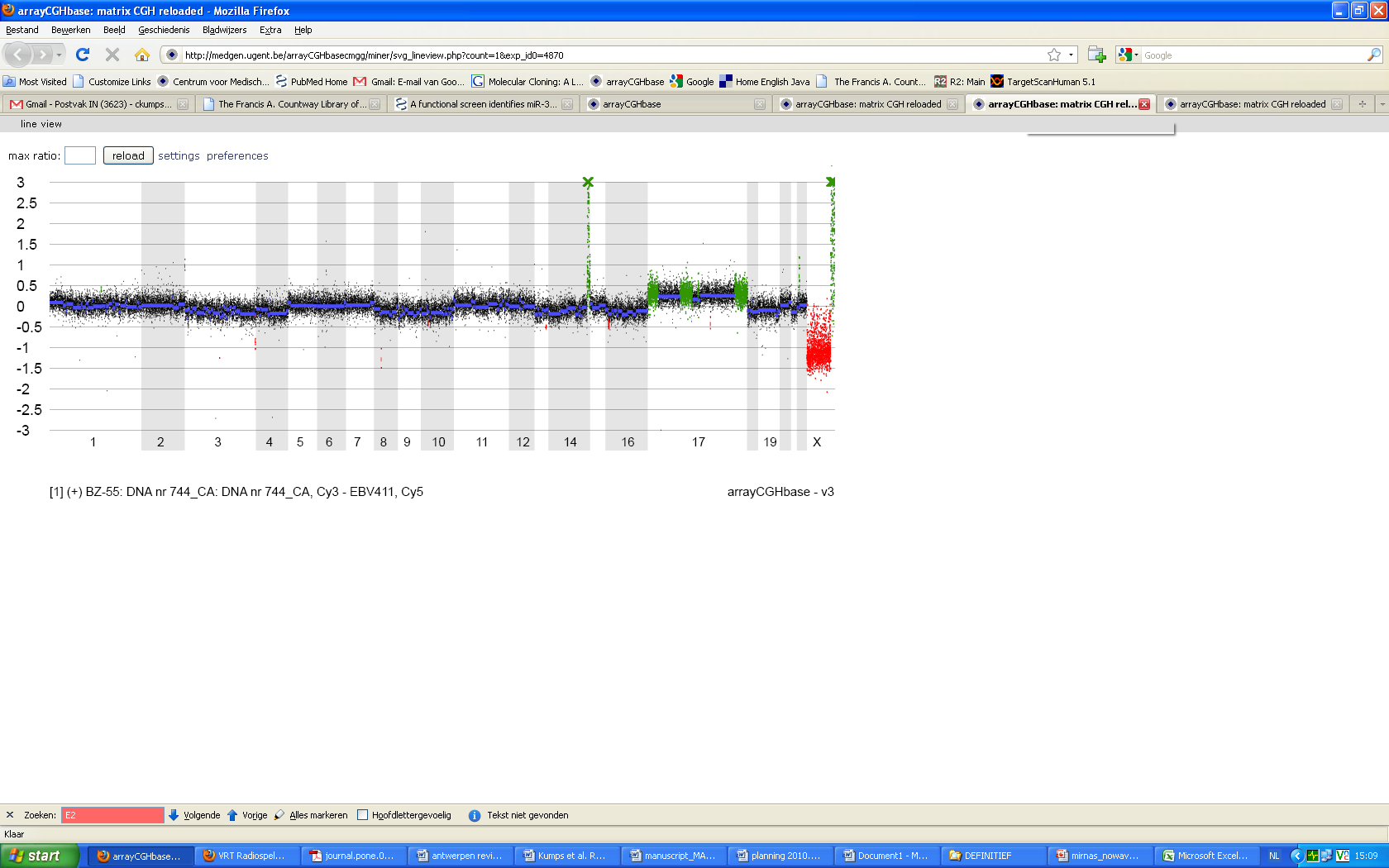
 NB_139


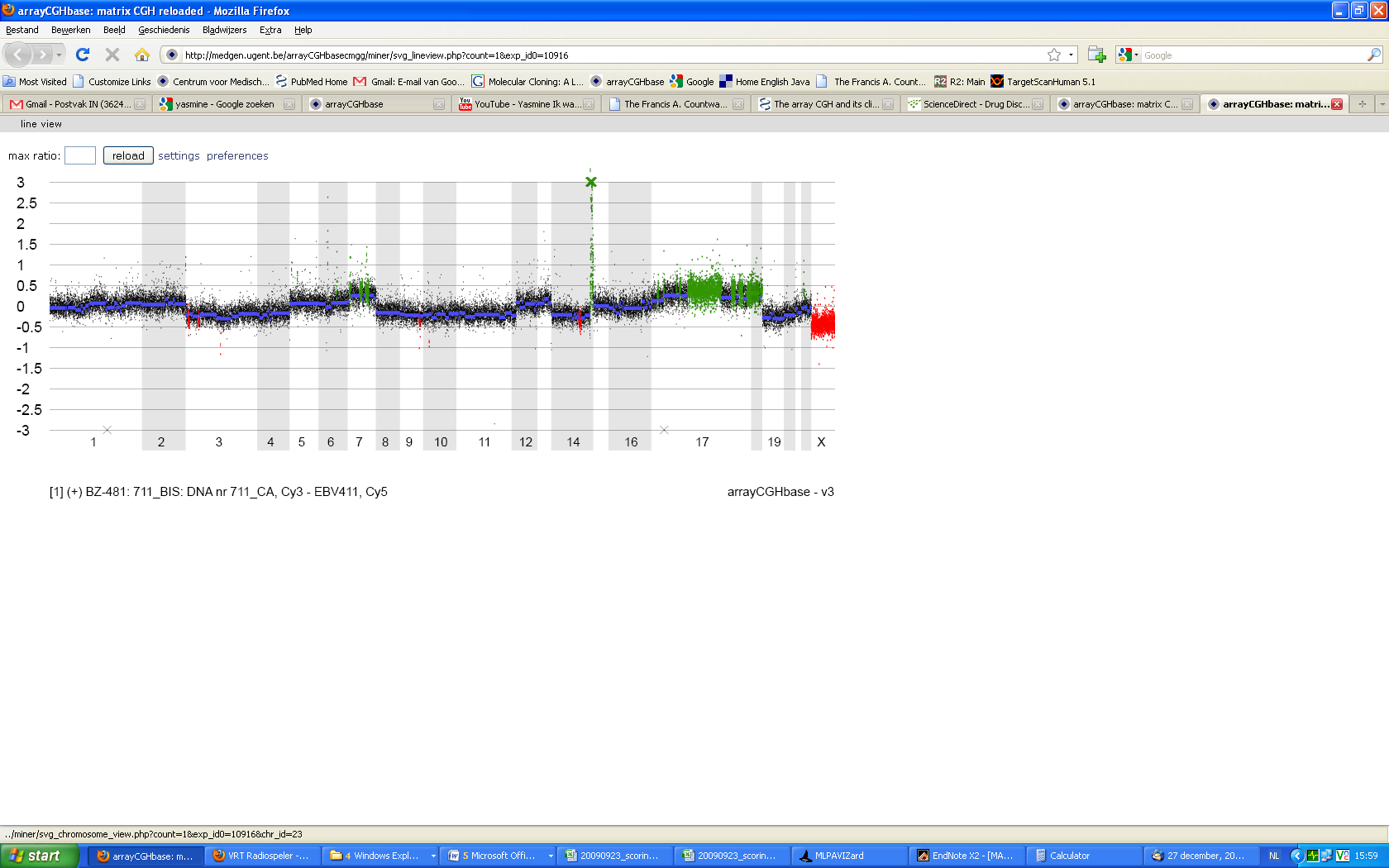
 NB_151


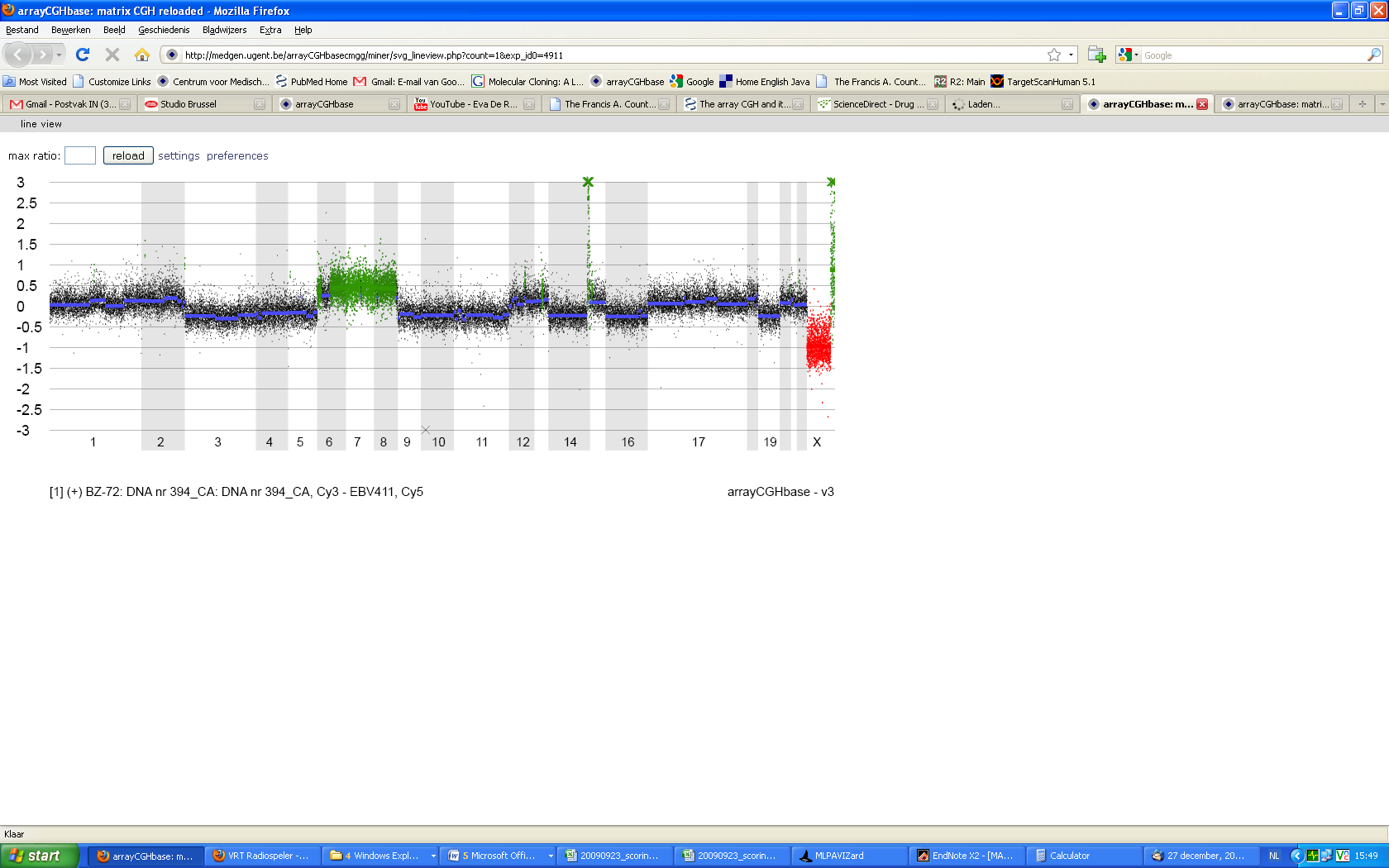
 NB_158


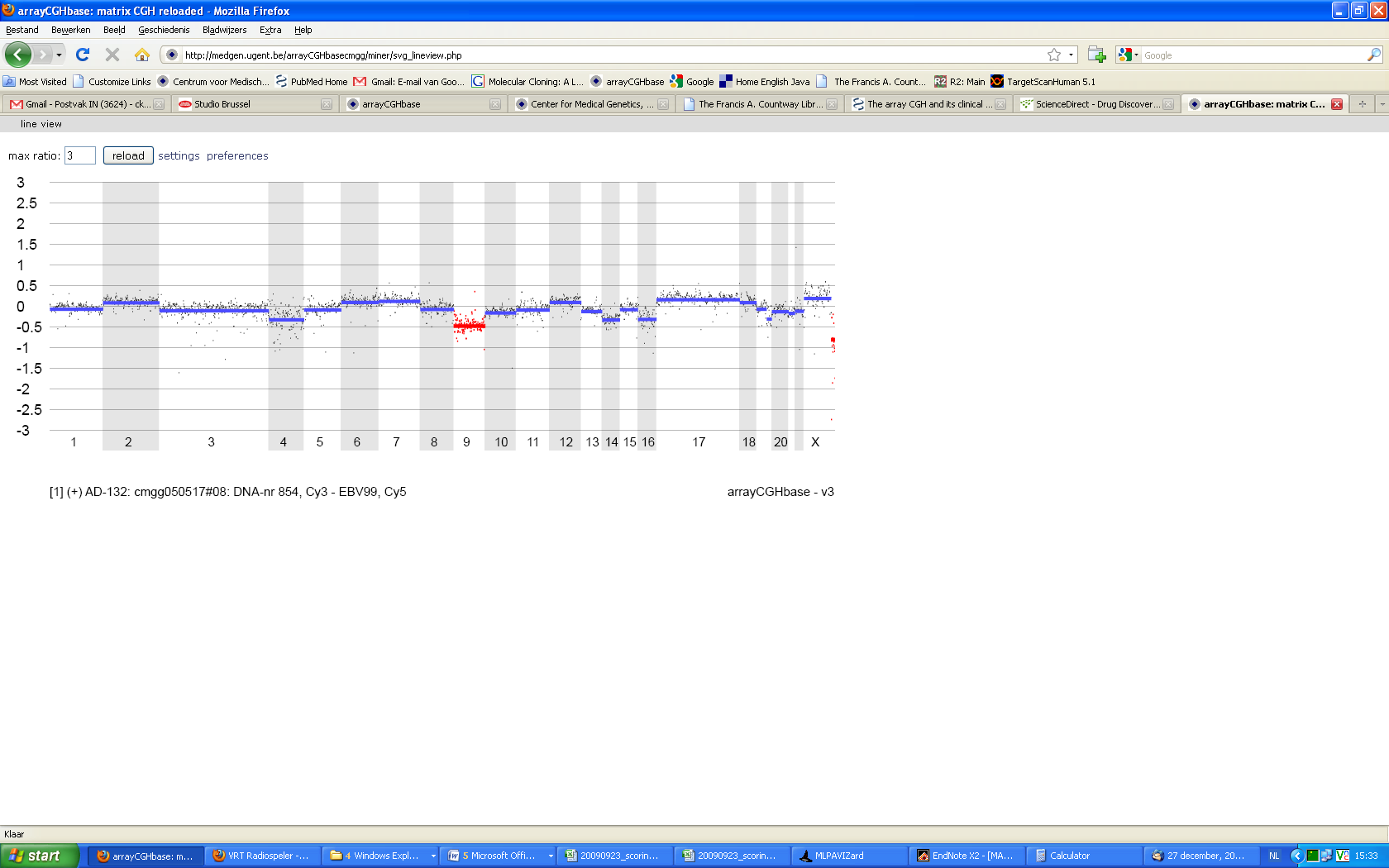
 NB_378


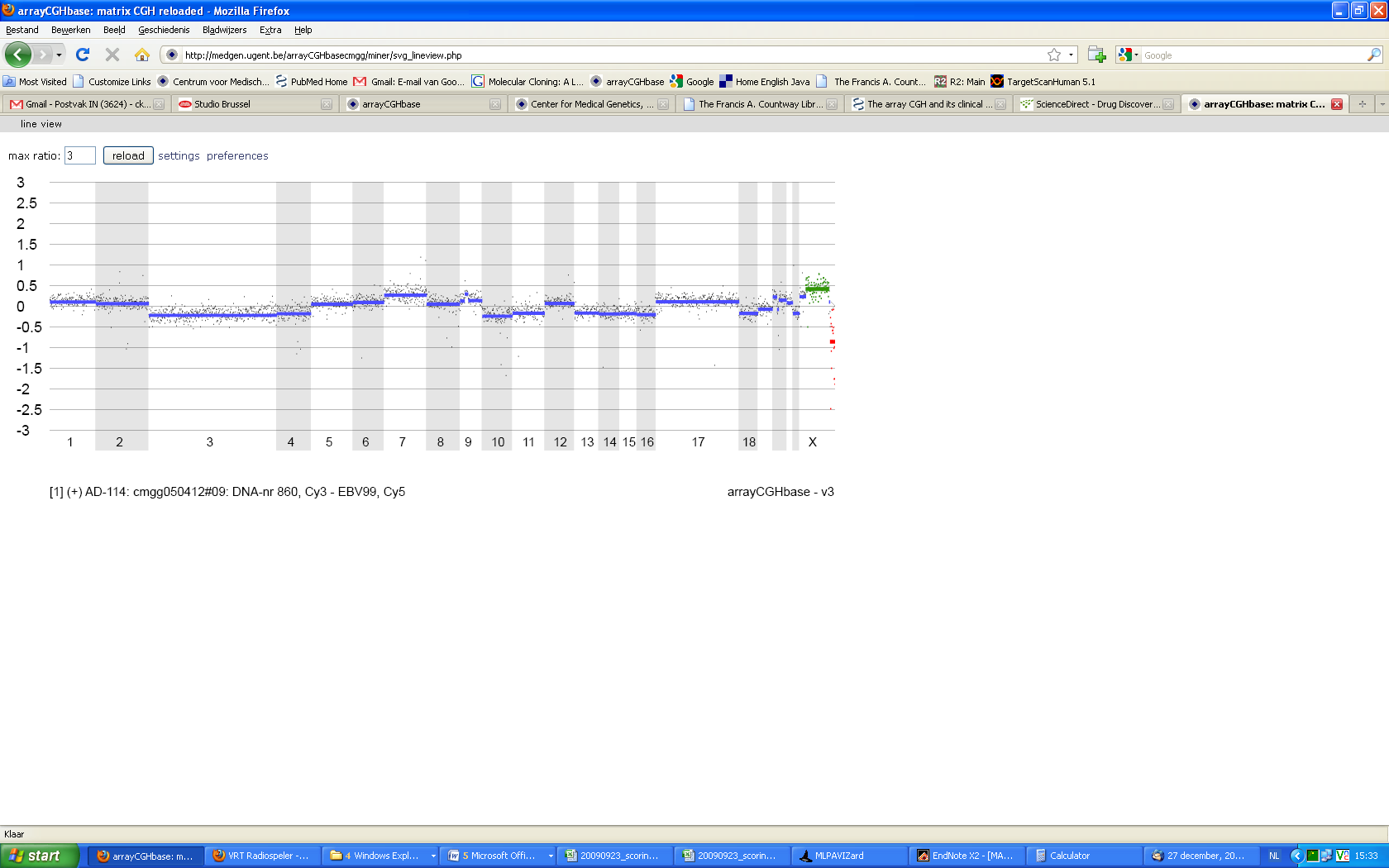
 NB_385


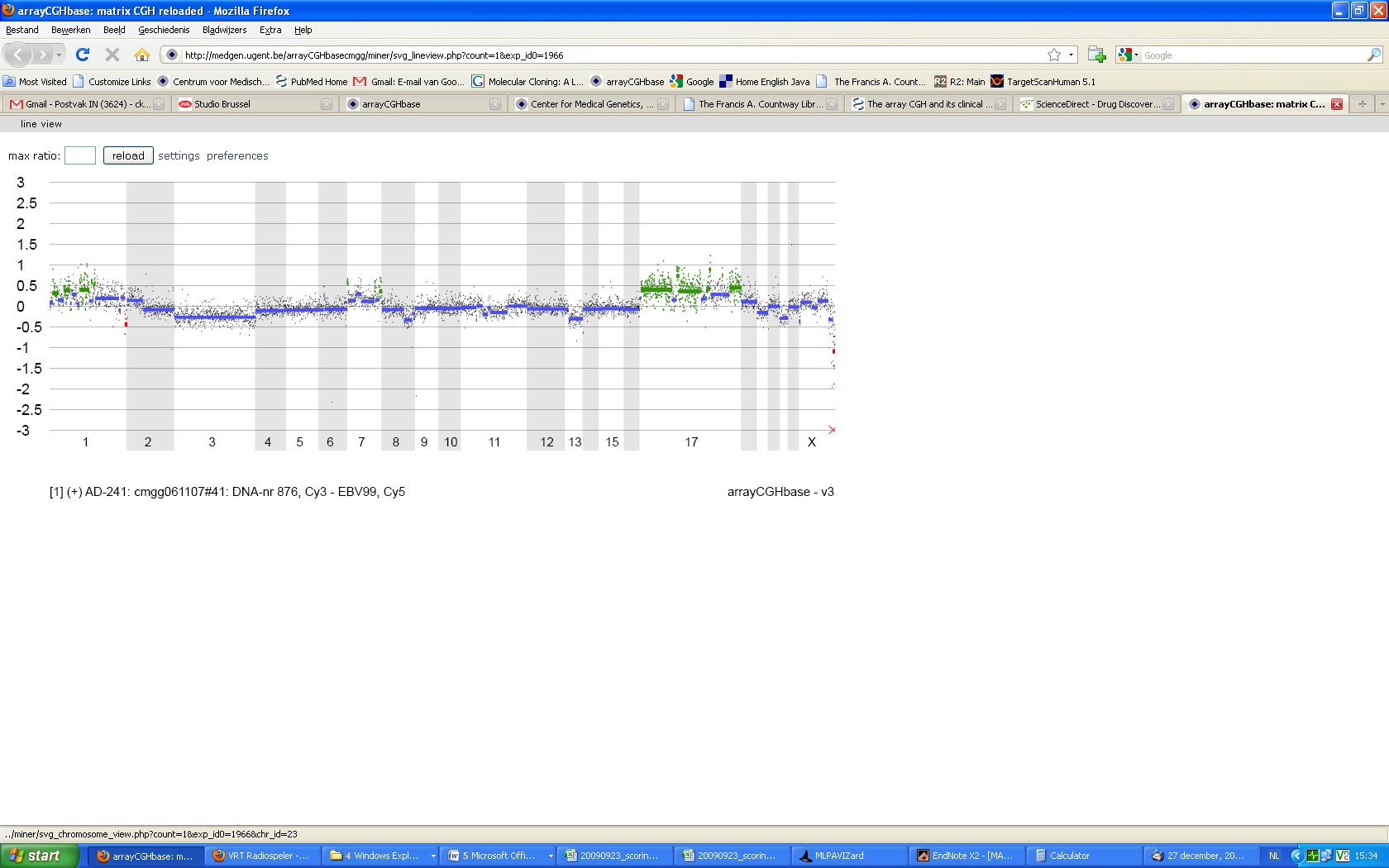
 NB_390


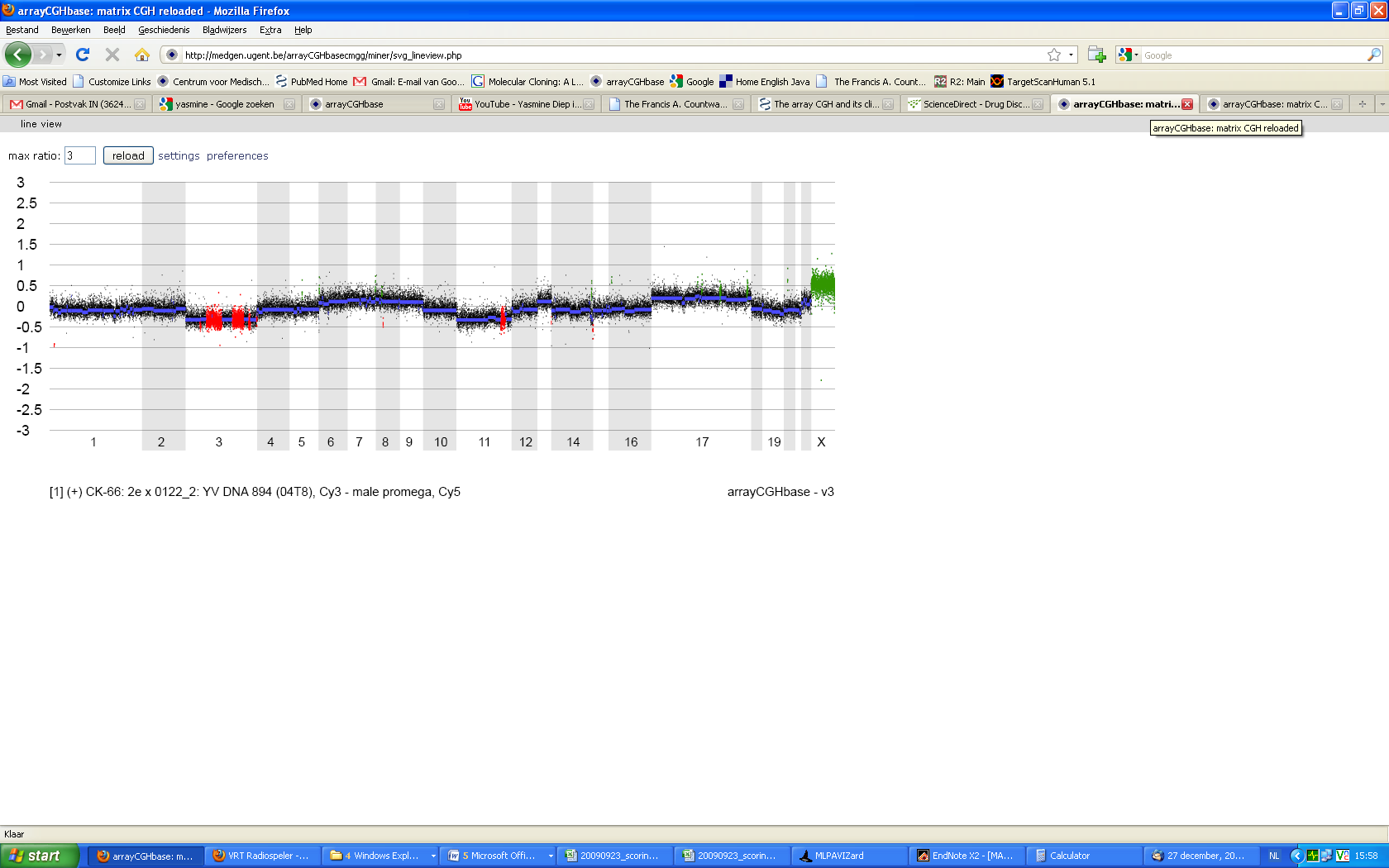
 NB_397


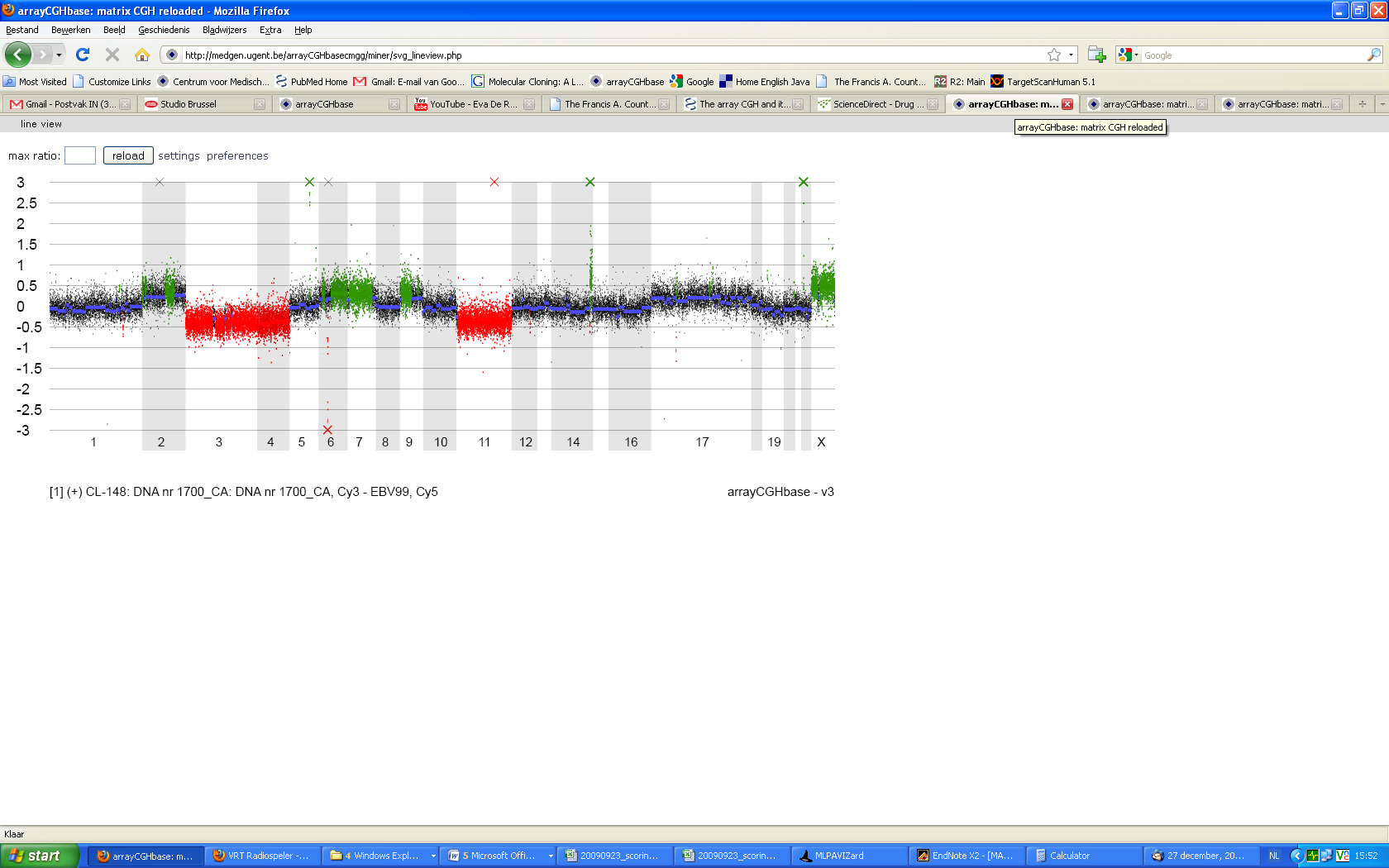
 NB_409


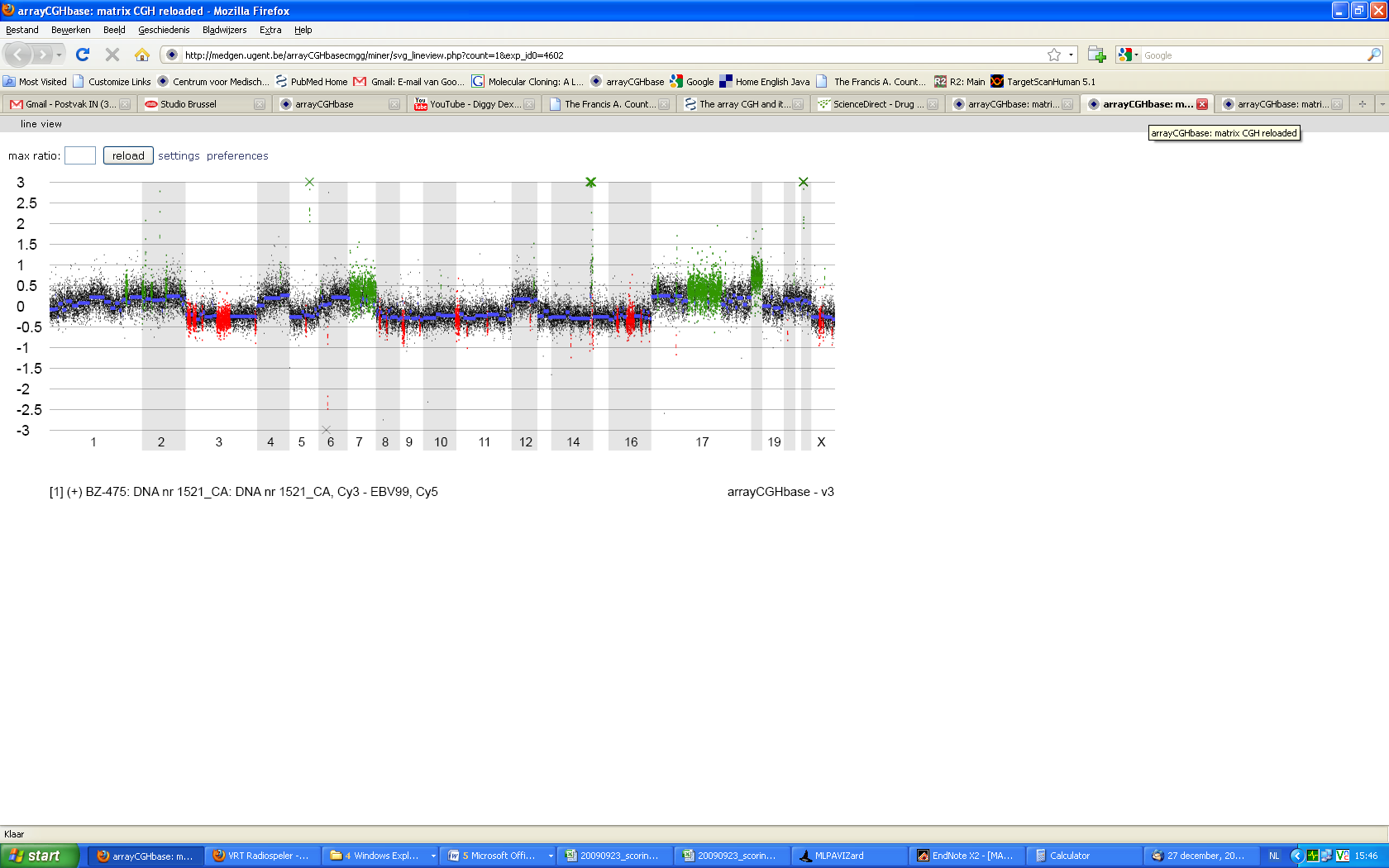
 NB_DN1521


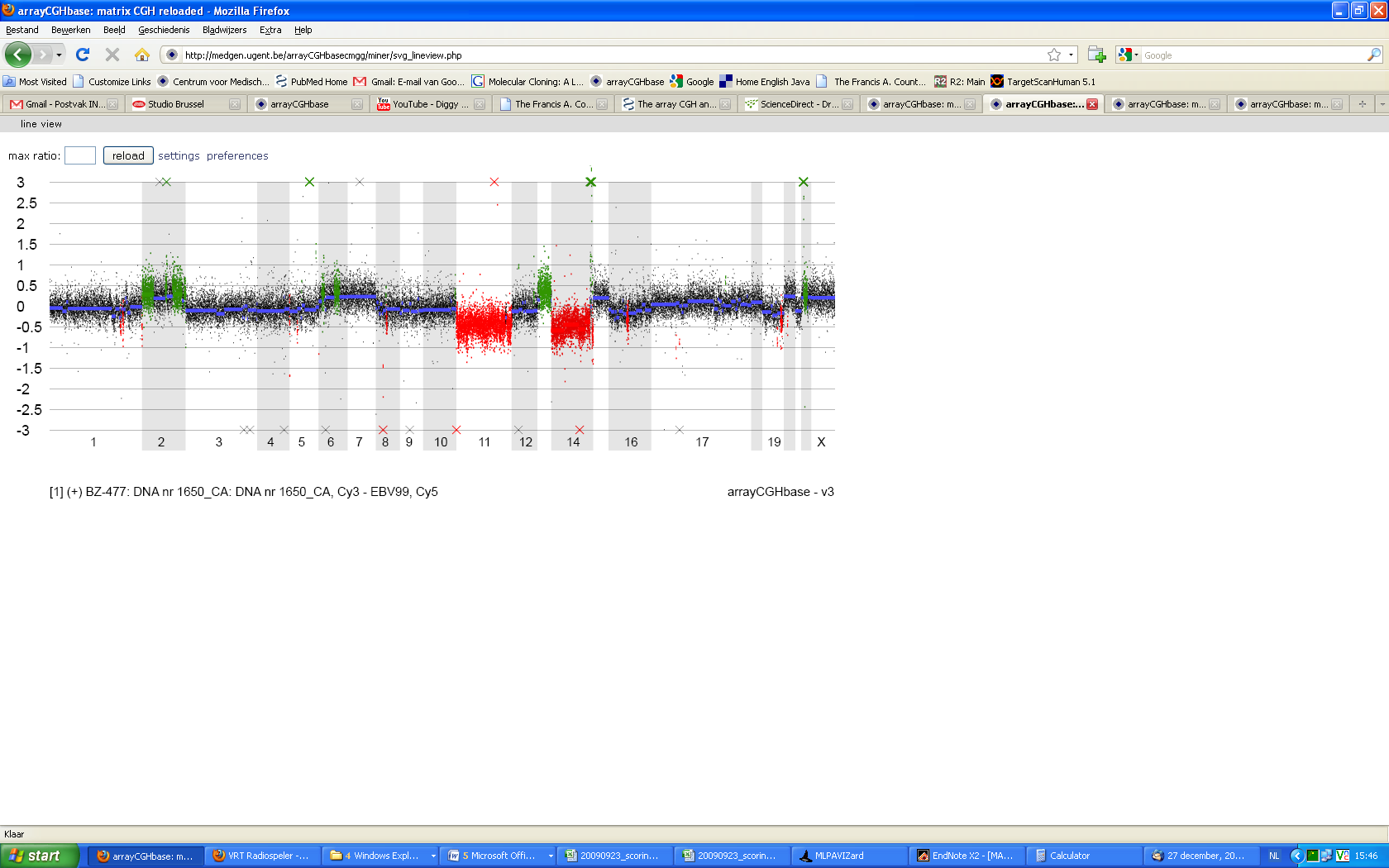
 NB_DN1650


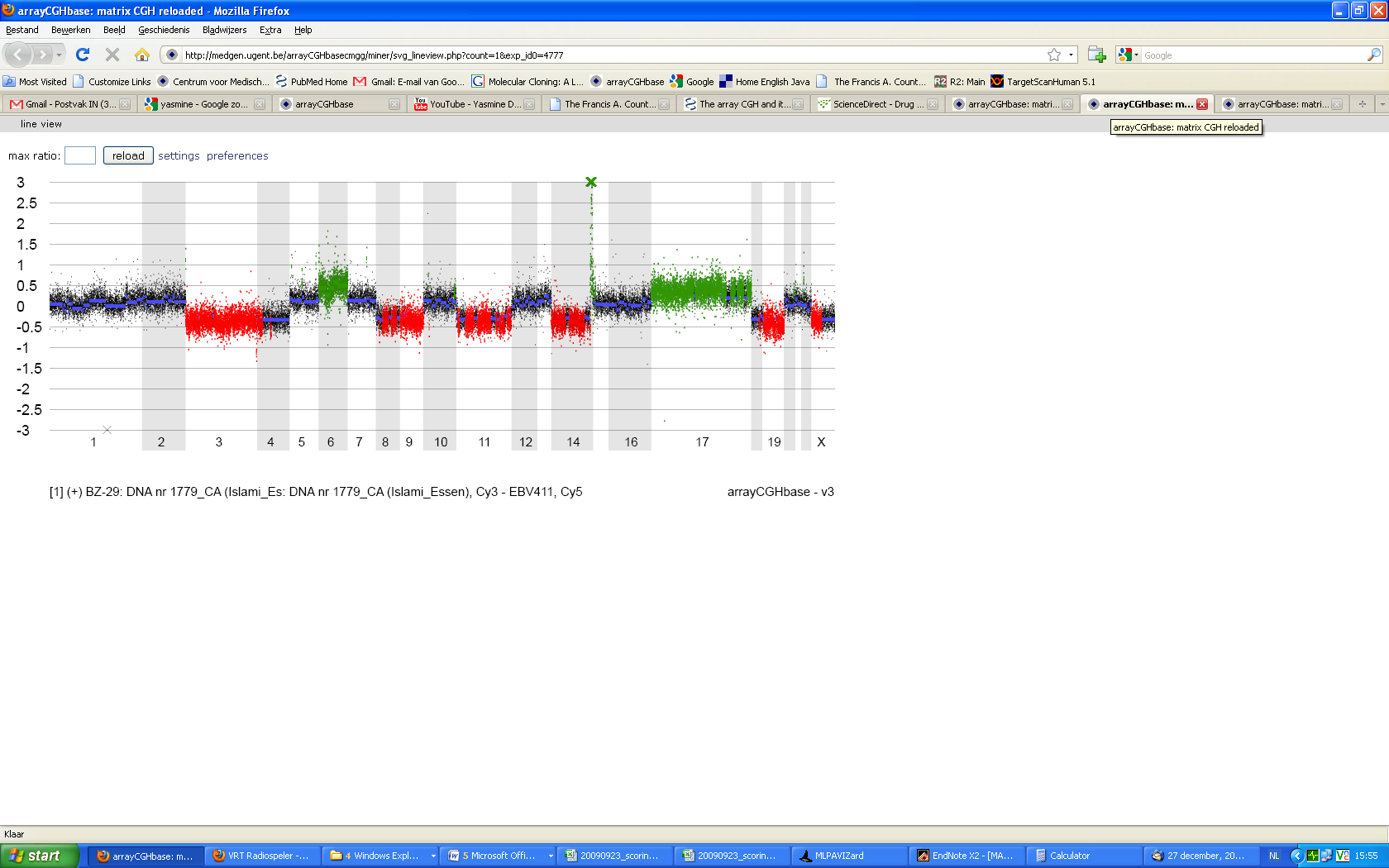
 NB_DN1862
