# Supplementary material for: Multiplex Amplicon Quantification (MAQ), a fast and efficient method for the simultaneous detection of copy number alterations in neuroblastoma
Source: BMC Genomics. 2010 May 12;11:298. doi: 10.1186/1471-2164-11-298 (PMC2879279; doi:10.1186/1471-2164-11-298)
Supplement: Additional file 5 — Overview of ROC curves of MLPA and MAQ assays versus array CGH. Overview of ROC curves of MLPA and MAQ assays versus array CGH at 1p, 2p, 3p, 11q and 17q for tumors with segmental and numerical aberrations. [file 1471-2164-11-298-S5.DOC]

**MLPA segmental MAQ segmental**


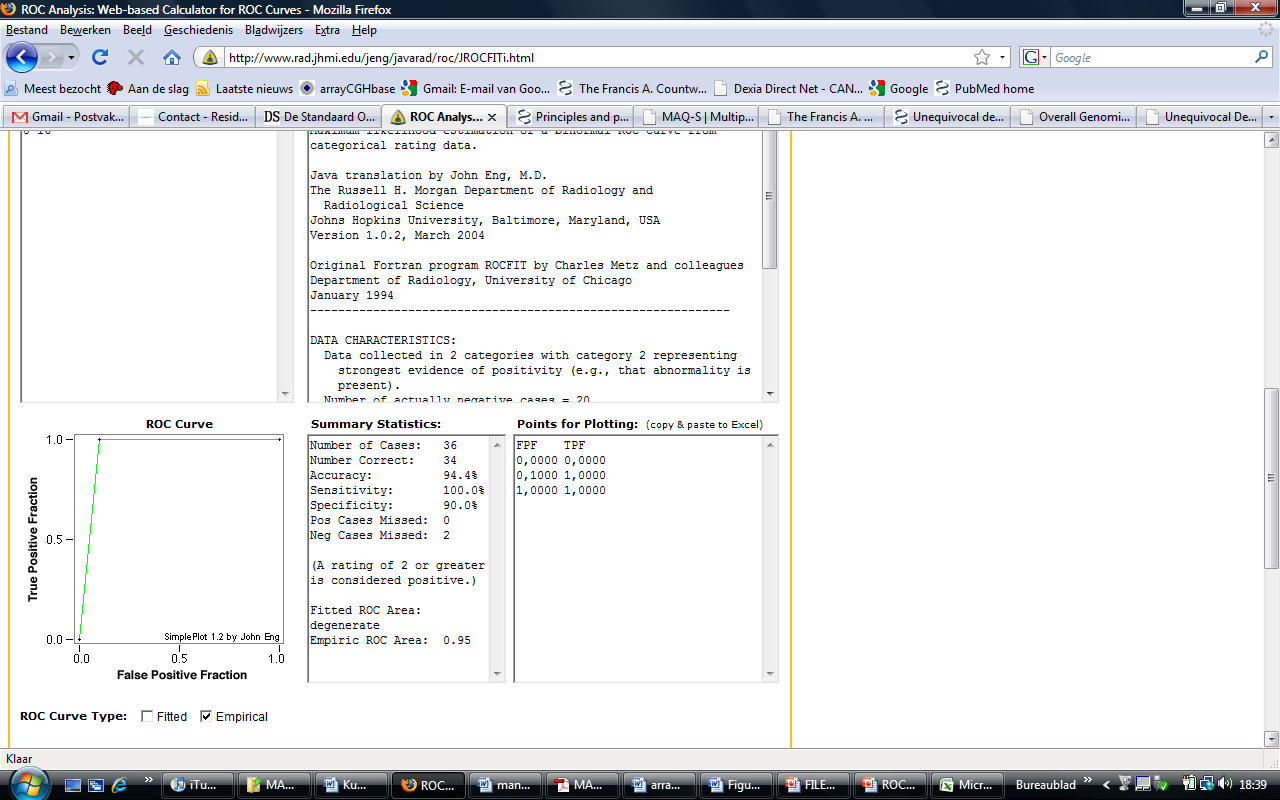

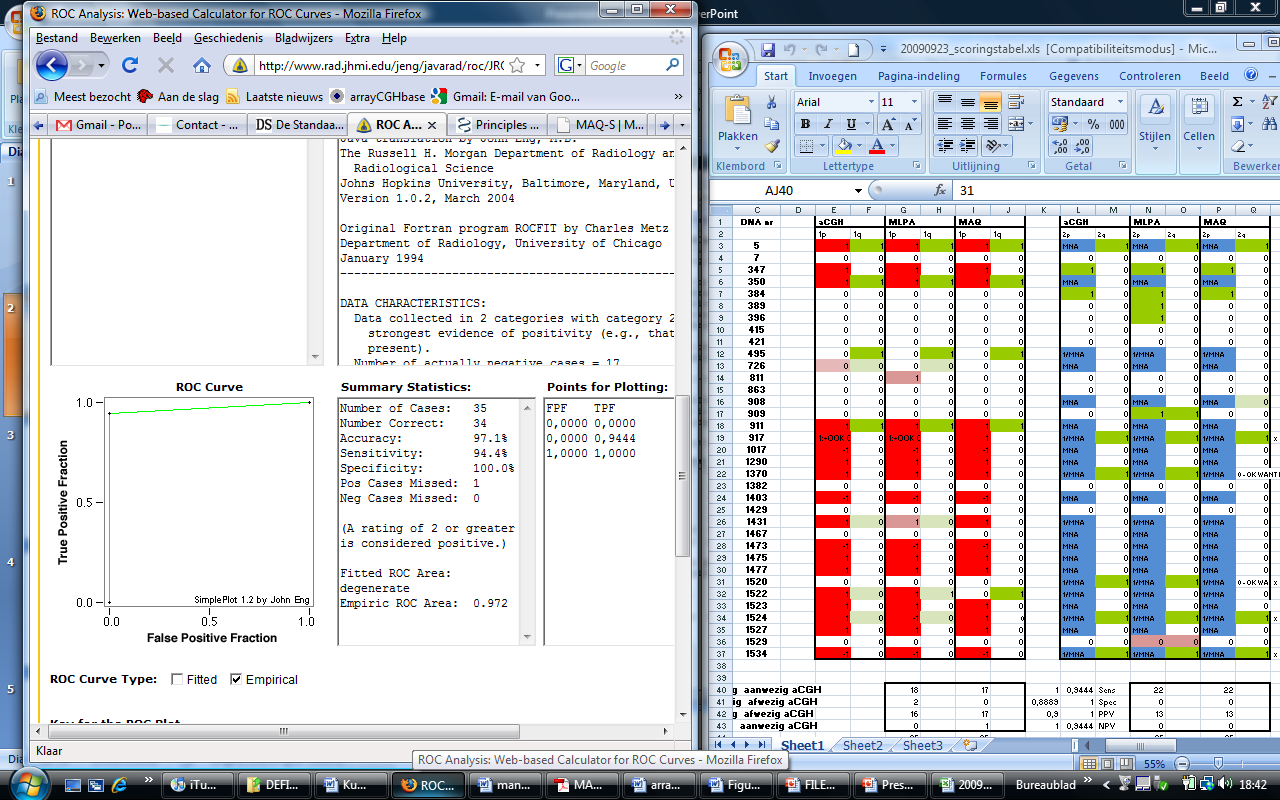


1p


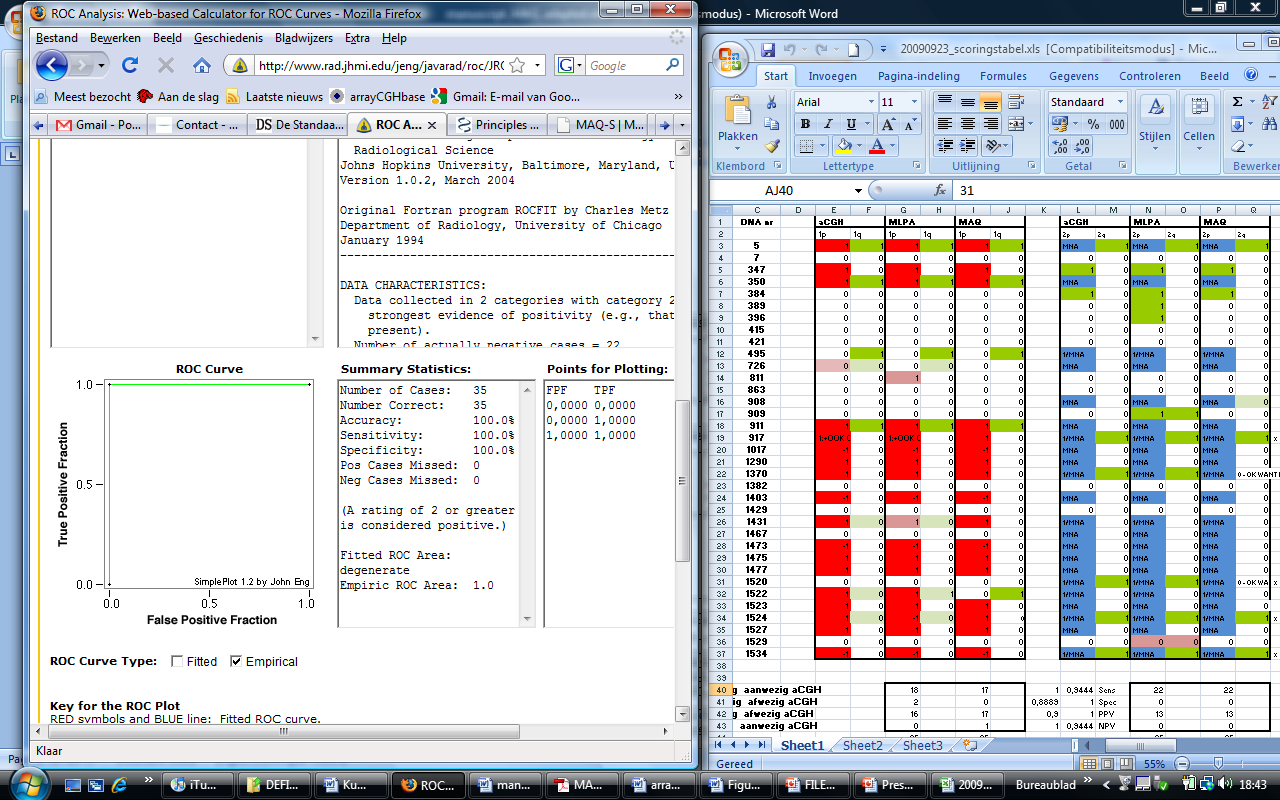

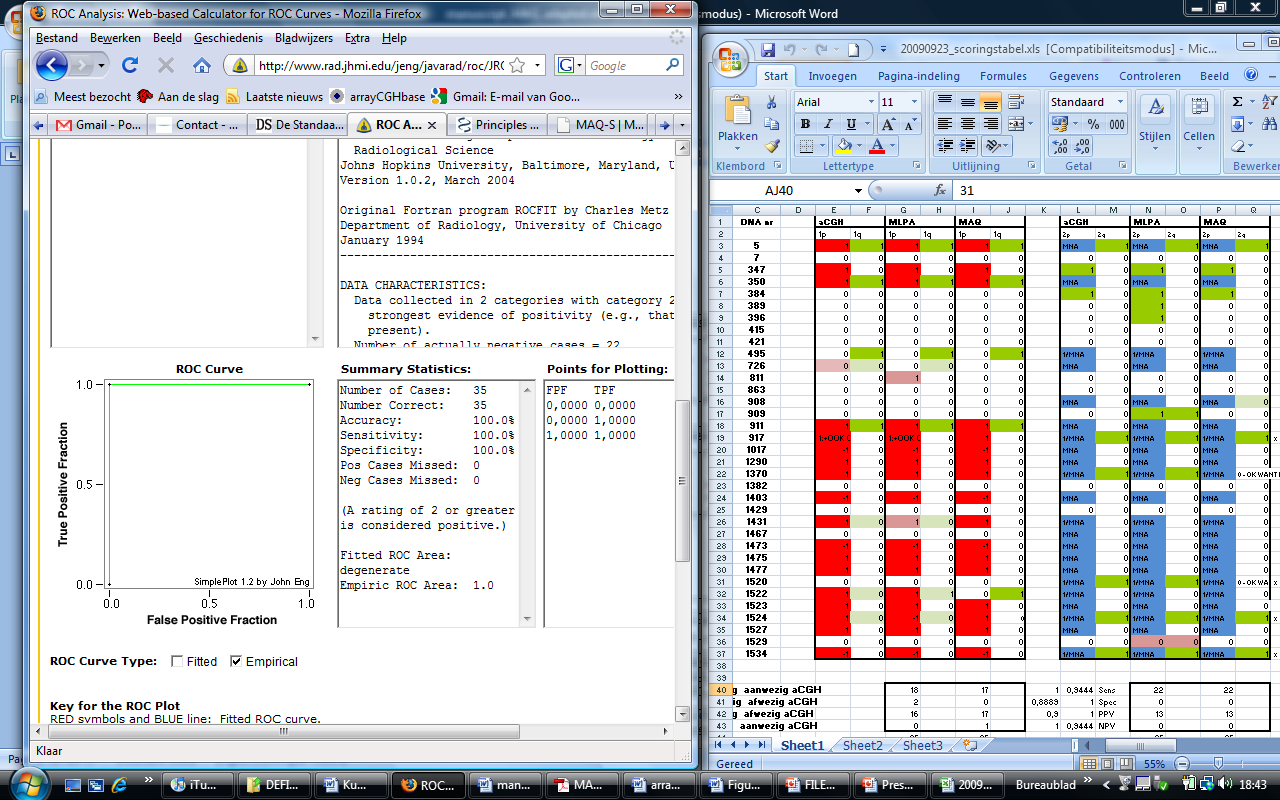


MNA


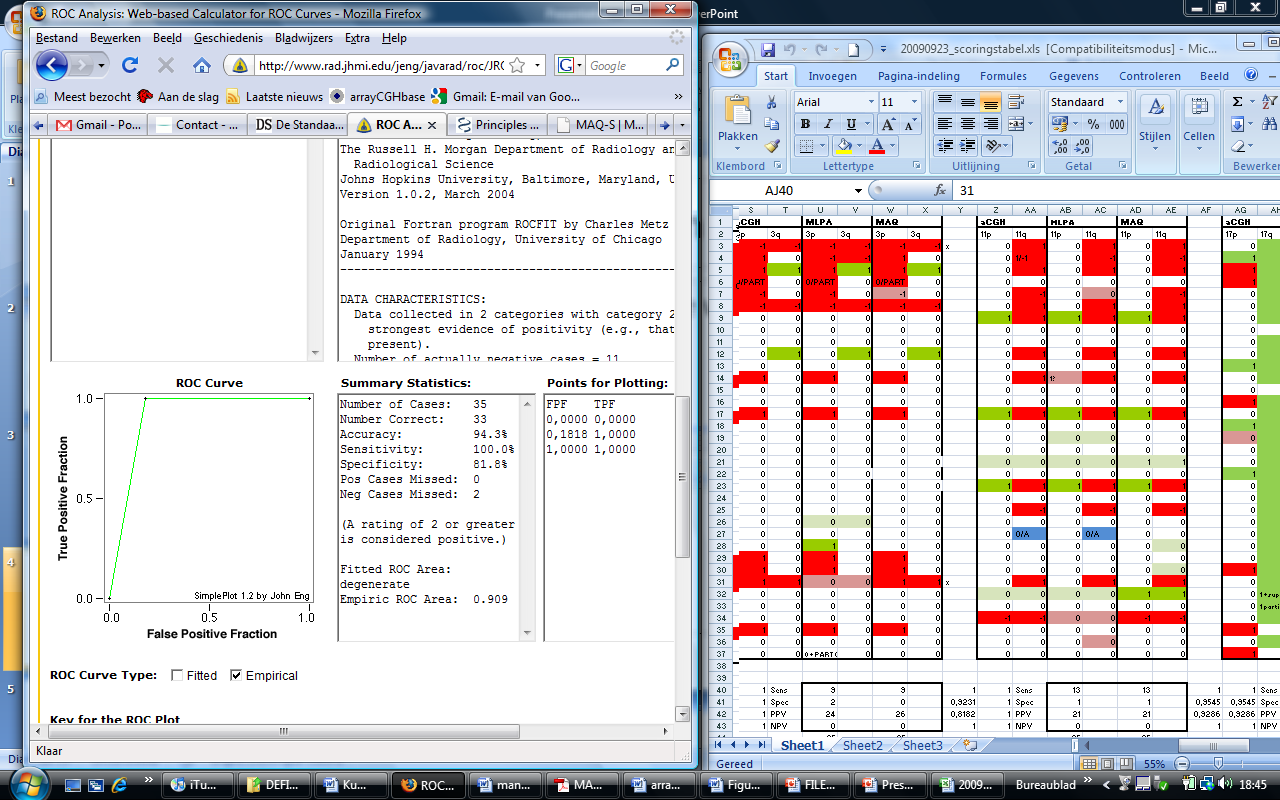

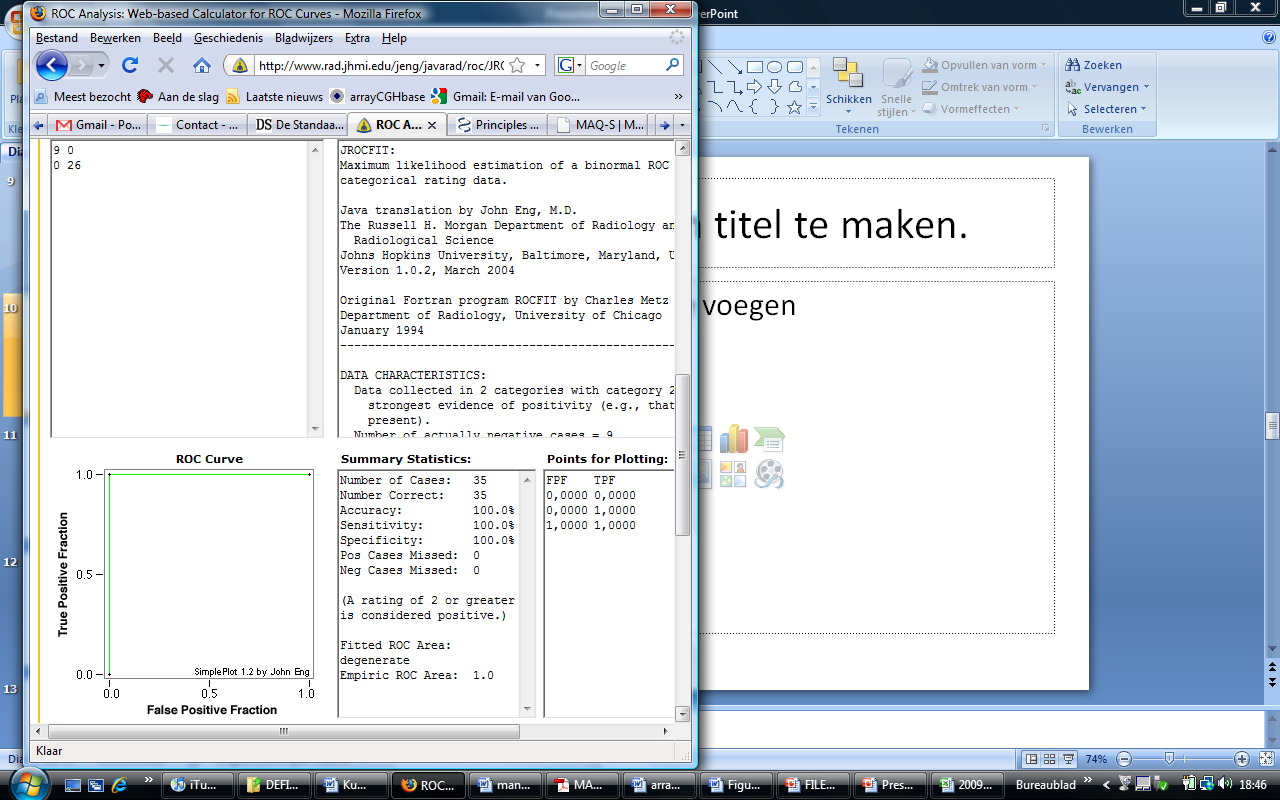


3p


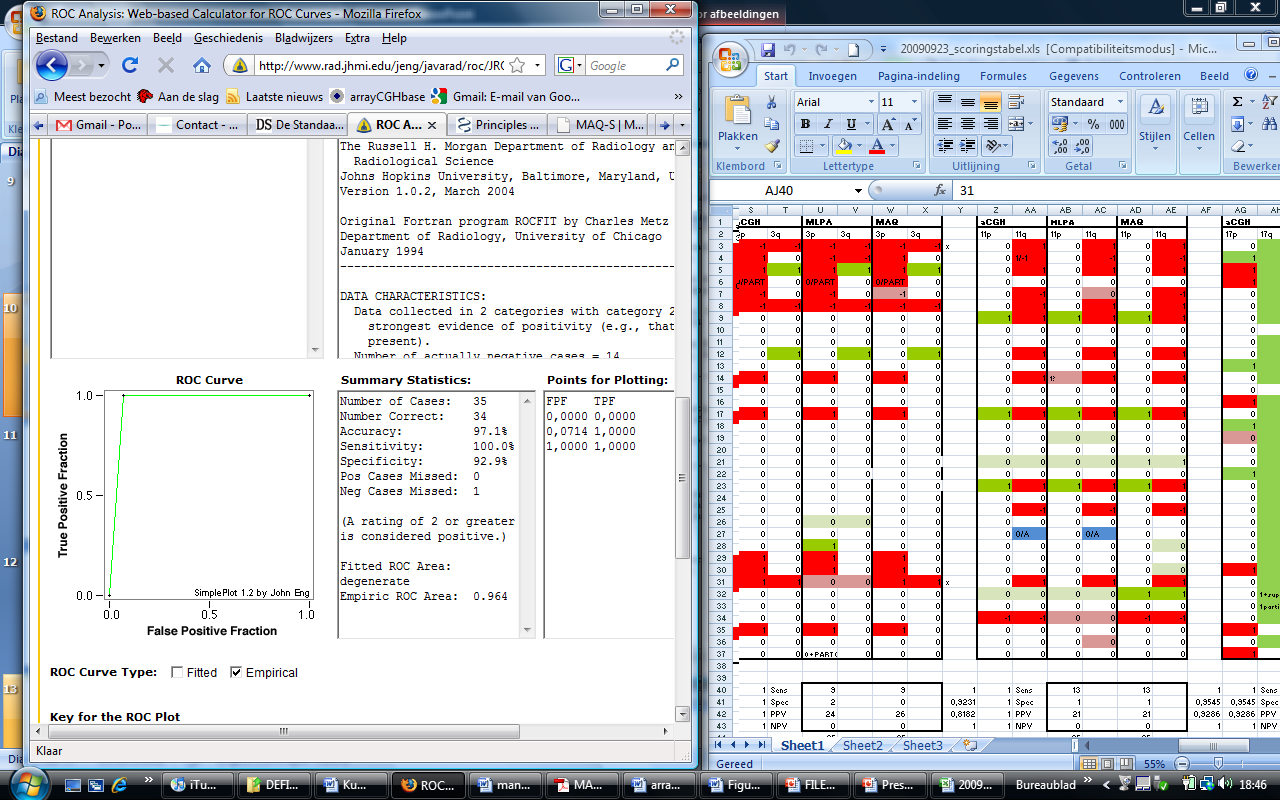

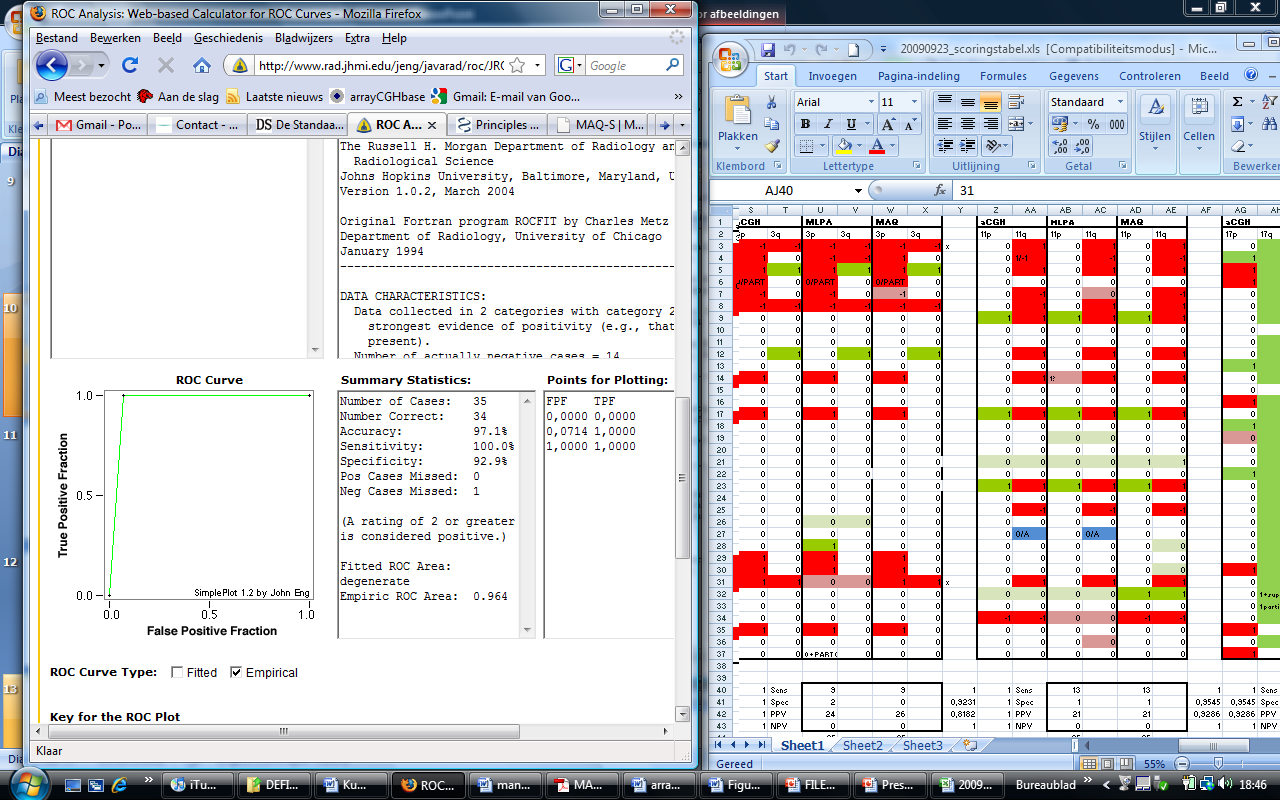

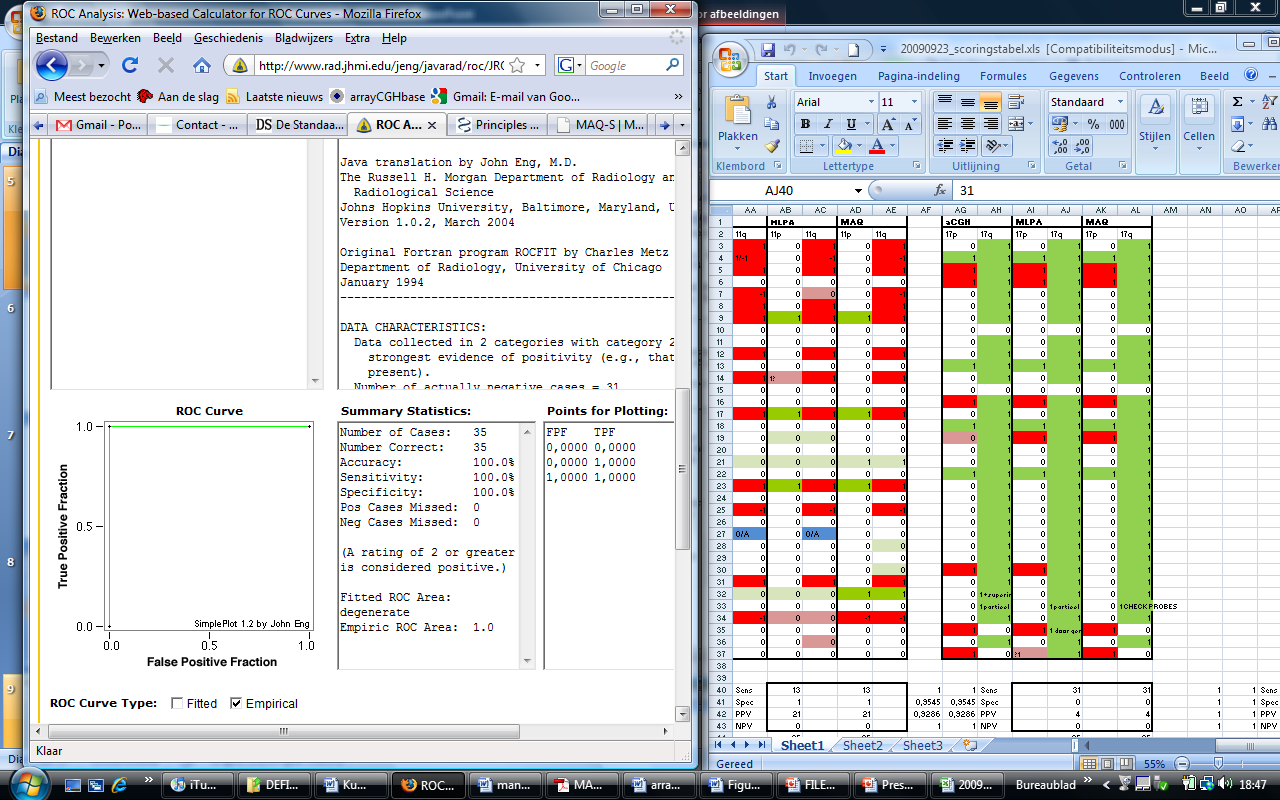

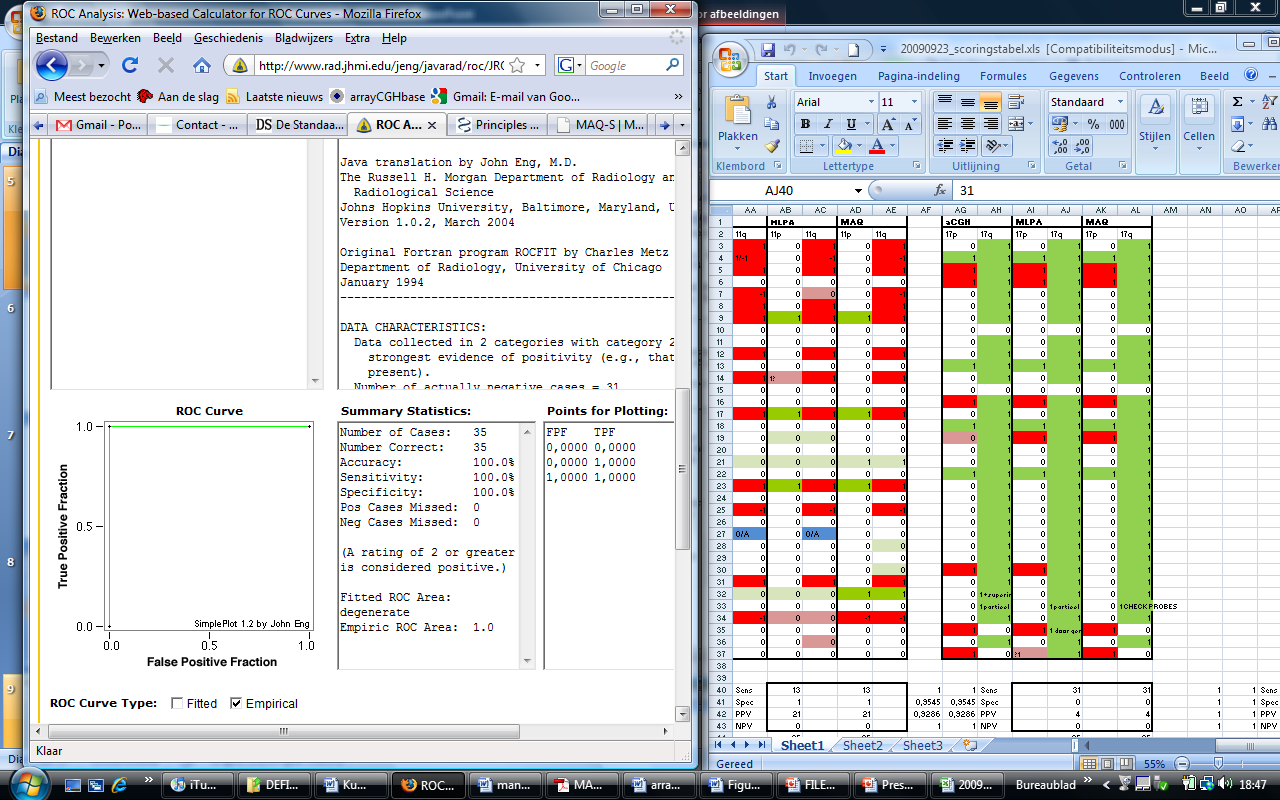


11q

17q

**MLPA numerical MAQ numerical**


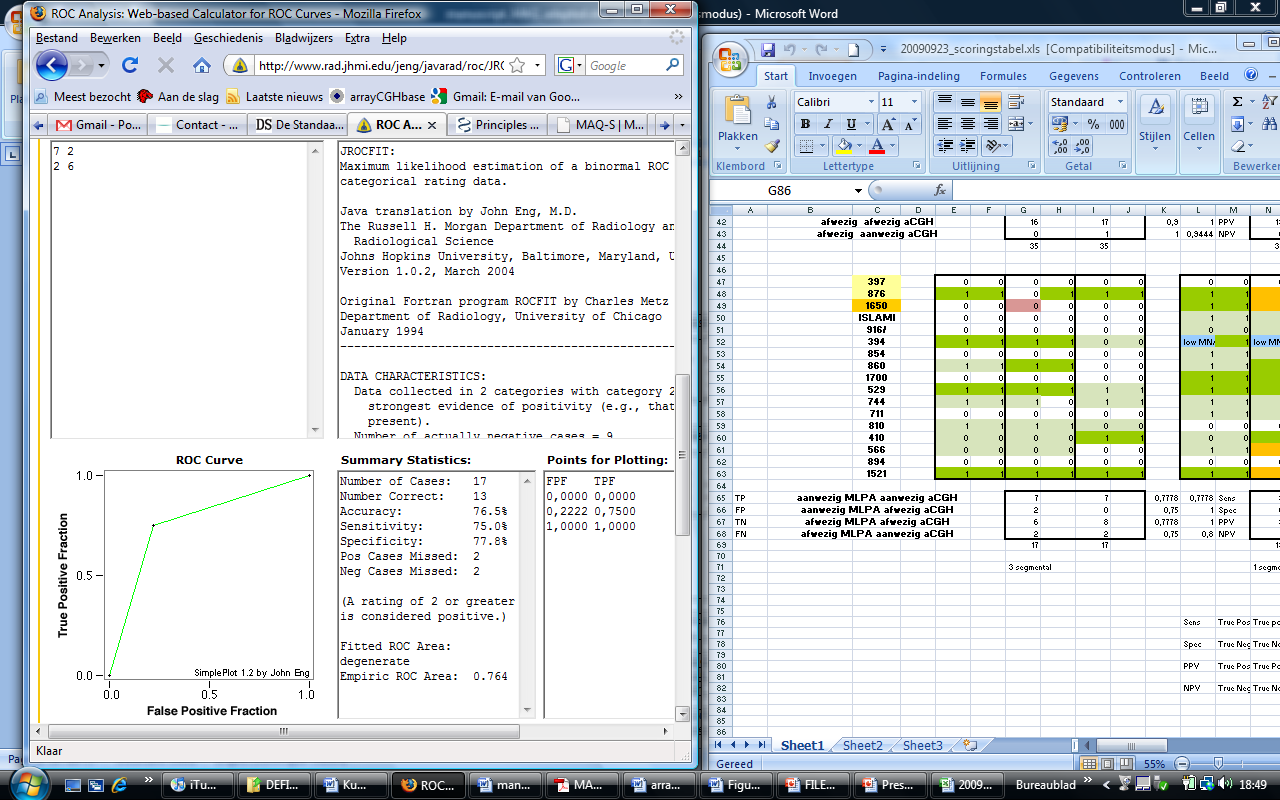

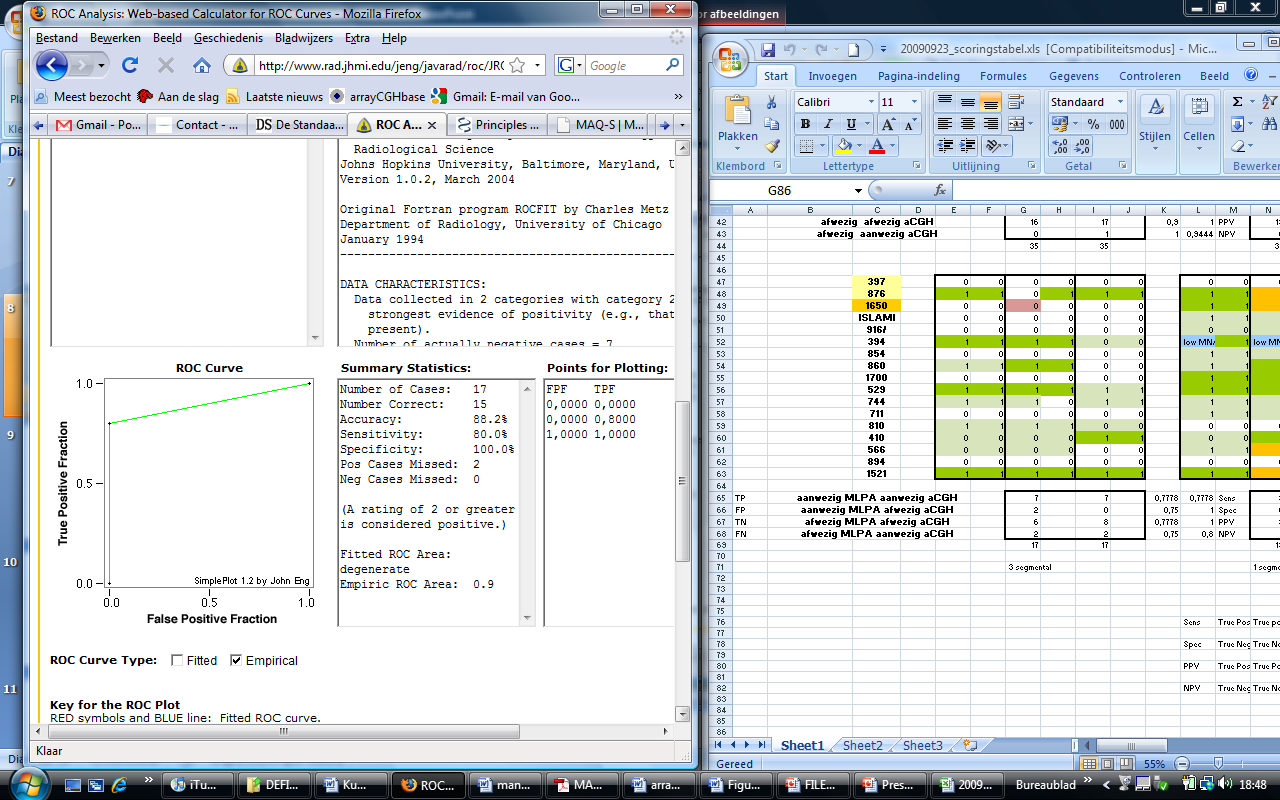

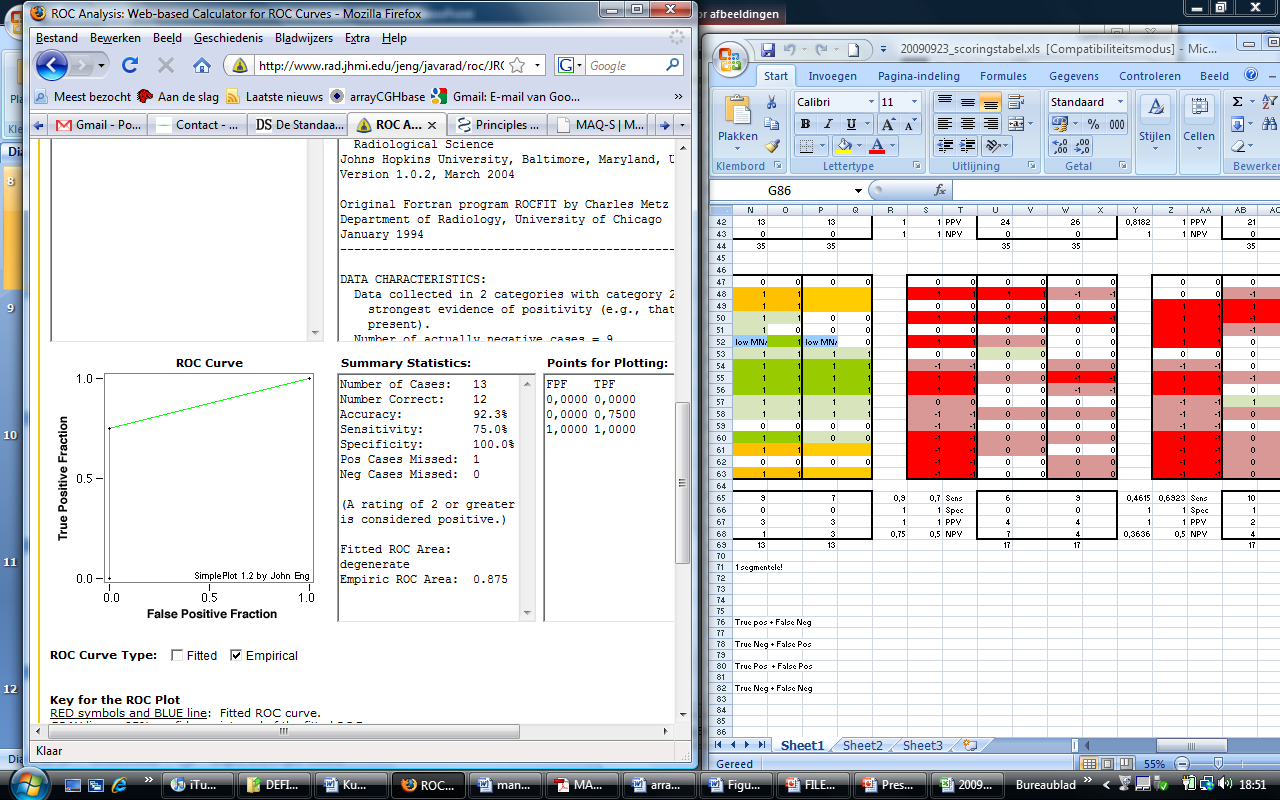

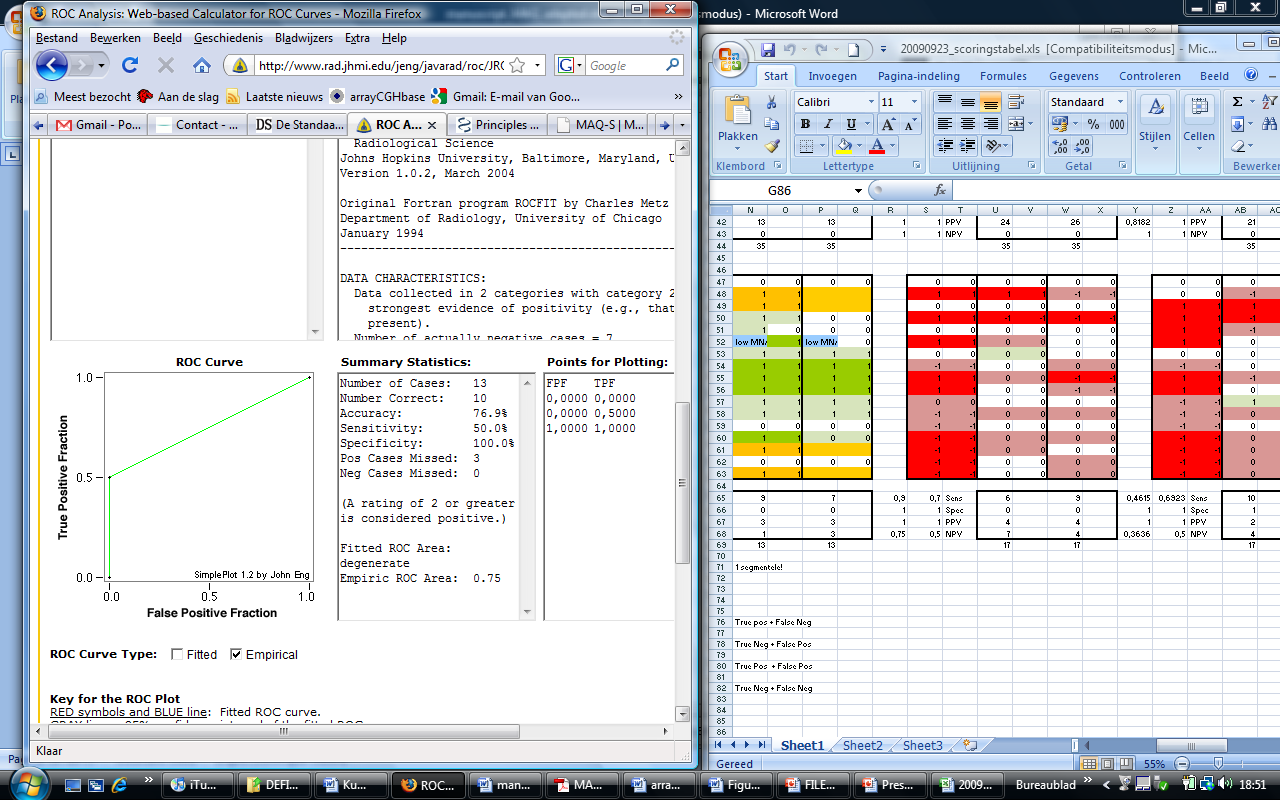

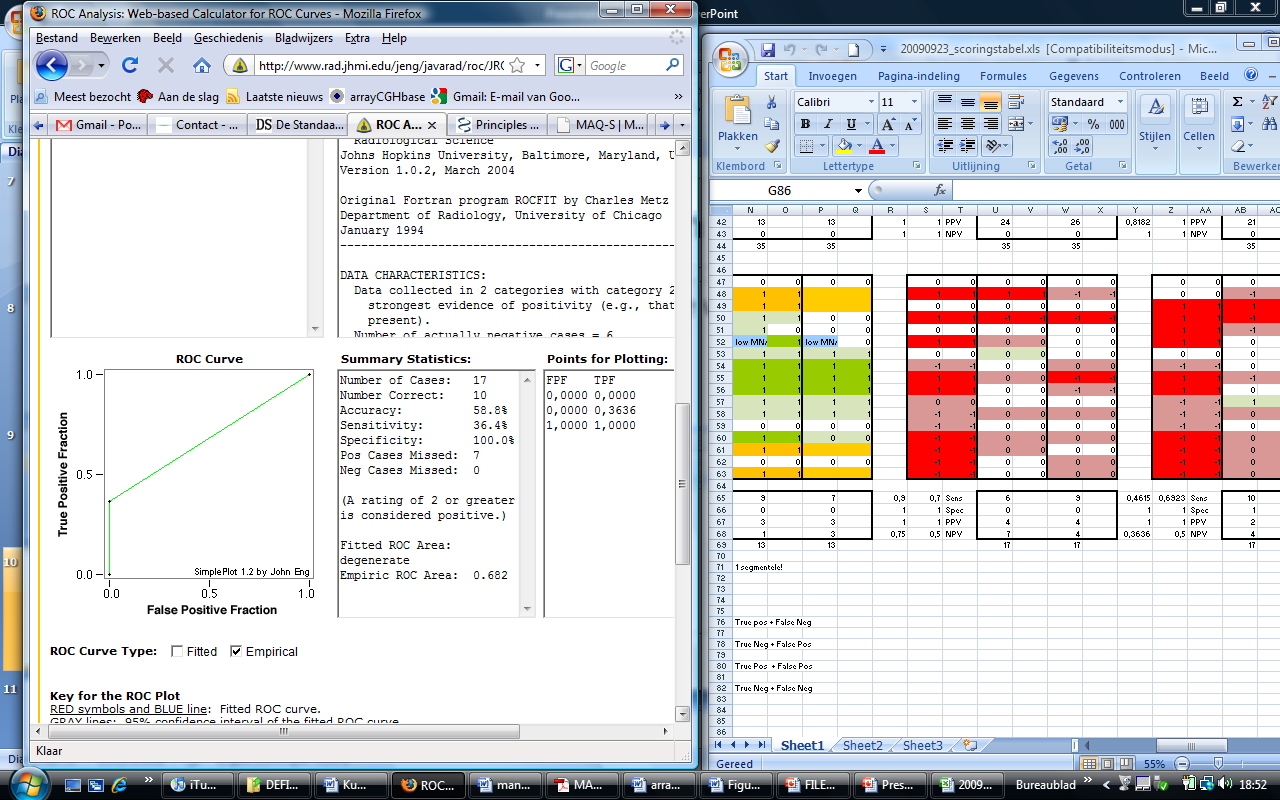

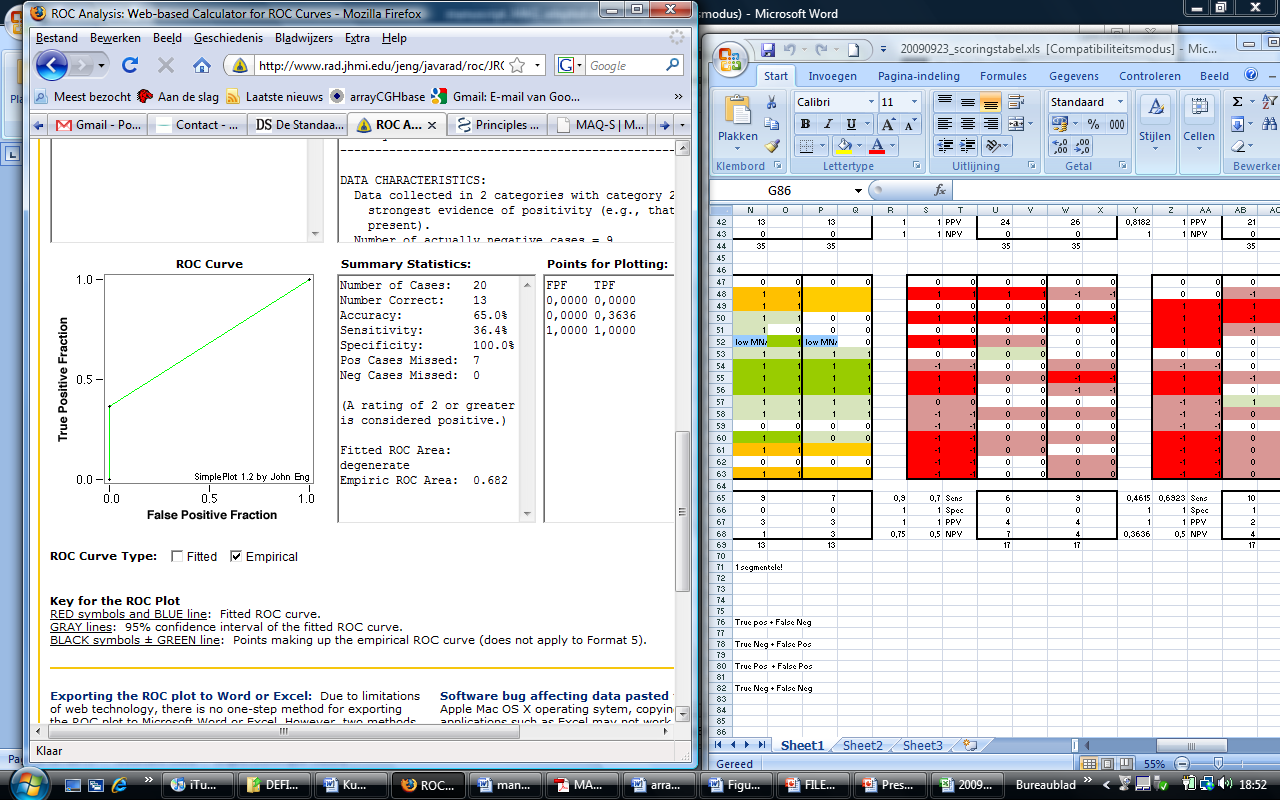

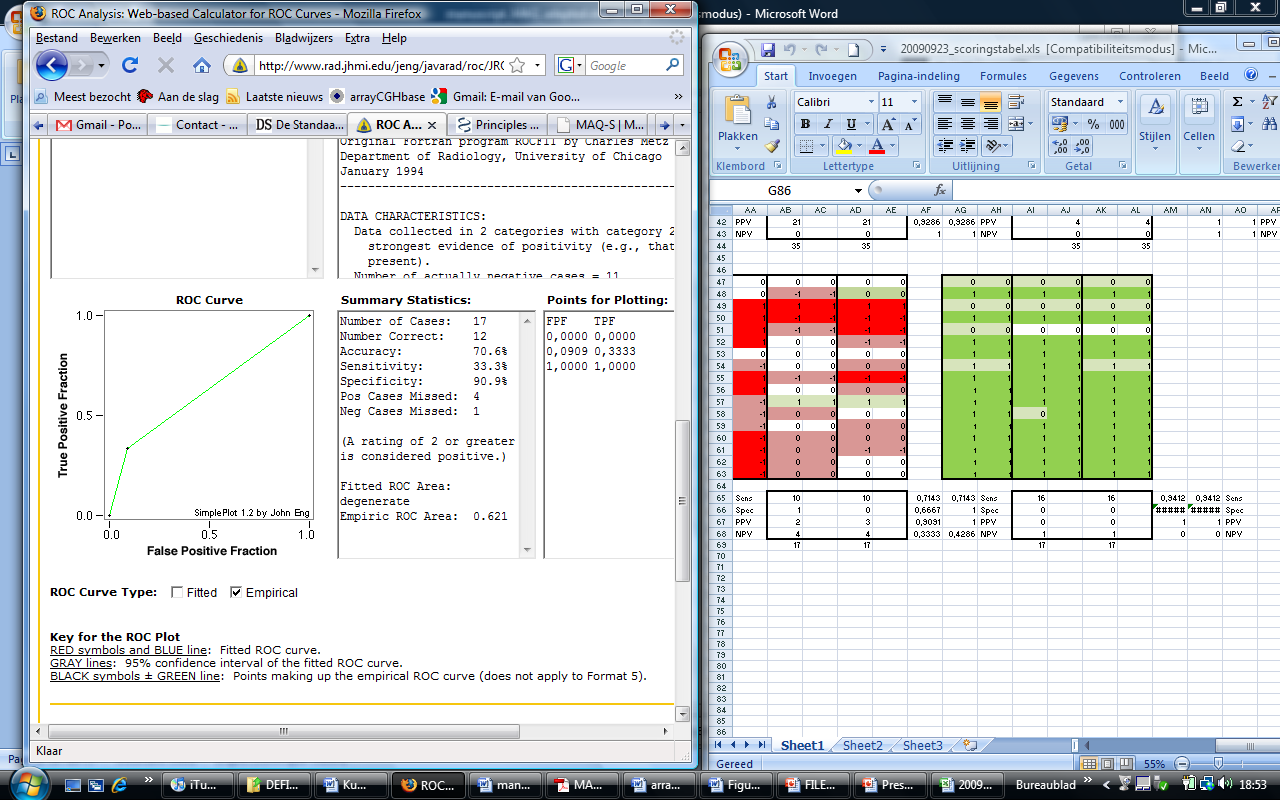

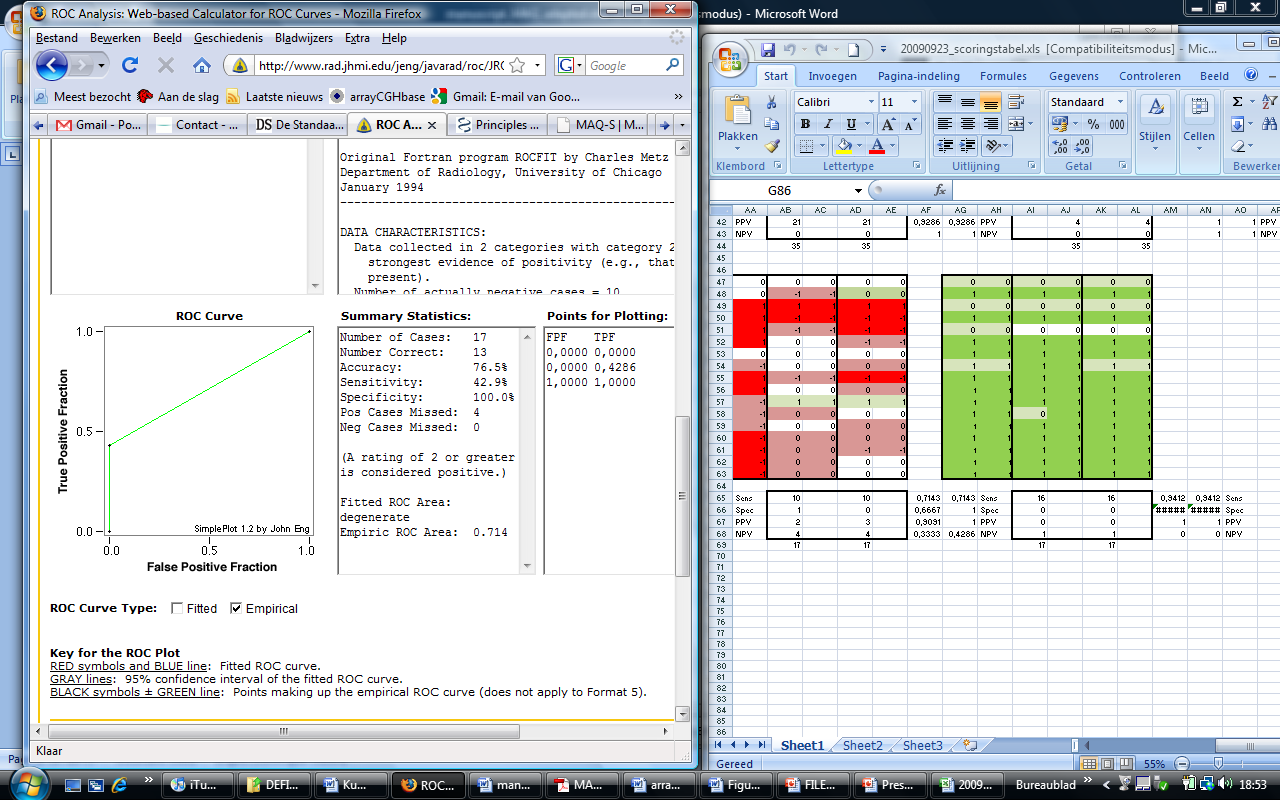


Chr1

Chr2

Chr3

Chr11

No ROC curves could be obtained for chromosome 17 in the tumors with only numerical aberrations, however the AUC is calculated as 94.1%. They all had whole chromosome 17 gain (of which one could not be detected) and hence no true negatives nor false positives could be detected.

Chr17
